# Antigenic drift and subtype interference shape A(H3N2) epidemic dynamics in the United States

**DOI:** 10.1101/2023.10.02.23296453

**Published:** 2023-10-03

**Authors:** Amanda C Perofsky, John Huddleston, Chelsea Hansen, John R Barnes, Thomas Rowe, Xiyan Xu, Rebecca Kondor, David E Wentworth, Nicola Lewis, Lynne Whittaker, Burcu Ermetal, Ruth Harvey, Monica Galiano, Rodney Stuart Daniels, John W McCauley, Seiichiro Fujisaki, Kazuya Nakamura, Noriko Kishida, Shinji Watanabe, Hideki Hasegawa, Sheena G Sullivan, Ian G Barr, Kanta Subbarao, Florian Krammer, Trevor Bedford, Cécile Viboud

**Affiliations:** 1 Fogarty International Center, National Institutes of Health, United States; 2 Brotman Baty Institute for Precision Medicine, University of Washington, United States; 3 Vaccine and Infectious Disease Division, Fred Hutchinson Cancer Center, United States; 4 Virology Surveillance and Diagnosis Branch, Influenza Division, National Center for Immunization and Respiratory Diseases (NCIRD), Centers for Disease Control and Prevention (CDC), United States; 5 WHO Collaborating Centre for Reference and Research on Influenza, Crick Worldwide Influenza Centre, The Francis Crick Institute, United Kingdom; 6 Influenza Virus Research Center, National Institute of Infectious Diseases, Japan; 7 WHO Collaborating Centre for Reference and Research on Influenza, The Peter Doherty Institute for Infection and Immunity, Department of Microbiology and Immunology, The University of Melbourne, The Peter Doherty Institute for Infection and Immunity, Australia; 8 Center for Vaccine Research and Pandemic Preparedness (C-VaRPP), Icahn School of Medicine at Mount Sinai, United States; 9 Department of Pathology, Molecular and Cell-Based Medicine, Icahn School of Medicine at Mount Sinai, United States; 10 Department of Genome Sciences, University of Washington, United States; 11 Howard Hughes Medical Institute, Seattle, United States

## Abstract

Influenza viruses continually evolve new antigenic variants, through mutations in epitopes of their major surface proteins, hemagglutinin (HA) and neuraminidase (NA). Antigenic drift potentiates the reinfection of previously infected individuals, but the contribution of this process to variability in annual epidemics is not well understood. Here we link influenza A(H3N2) virus evolution to regional epidemic dynamics in the United States during 1997—2019. We integrate phenotypic measures of HA antigenic drift and sequence-based measures of HA and NA fitness to infer antigenic and genetic distances between viruses circulating in successive seasons. We estimate the magnitude, severity, timing, transmission rate, age-specific patterns, and subtype dominance of each regional outbreak and find that genetic distance based on broad sets of epitope sites is the strongest evolutionary predictor of A(H3N2) virus epidemiology. Increased HA and NA epitope distance between seasons correlates with larger, more intense epidemics, higher transmission, greater A(H3N2) subtype dominance, and a greater proportion of cases in adults relative to children, consistent with increased population susceptibility. Based on random forest models, A(H1N1) incidence impacts A(H3N2) epidemics to a greater extent than viral evolution, suggesting that subtype interference is a major driver of influenza A virus infection dynamics, presumably via heterosubtypic cross-immunity.

## Introduction

Influenza viruses continually accumulate genetic changes in epitopes of two major surface proteins, hemagglutinin (HA) and neuraminidase (NA), in a process known as “antigenic drift.” Though individual hosts develop long-lasting immunity to specific influenza virus strains after infection, antigenic drift helps the virus to escape immune recognition, leaving previously exposed hosts susceptible to reinfection and necessitating the regular updates to the antigens included in the influenza vaccine [1]. While antigenic drift aids immune escape, prospective cohort studies and modeling of surveillance data also indicate that reinfection by antigenically homologous viruses occurs on average every 1–4 years, due to the waning of protection over time and antigenic drift [2,3].

Among the influenza virus types that routinely co-circulate in humans (A and B), type A viruses, particularly subtype A(H3N2), experience the fastest rates of antigenic evolution and cause the most substantial morbidity and mortality [4–7]. Seasonal influenza A viruses (IAV) cause annual winter epidemics in temperate zones of the Northern and Southern Hemispheres and circulate year-round in tropical regions [8]. Influenza A epidemic burden fluctuates substantially from year to year [9], and there is much scientific interest in disentangling the relative roles of viral evolution, prior immunity, human behavior, and climatic factors in driving this seasonal variability. Climatic factors, such as humidity and temperature, have been implicated in the seasonality and timing of winter outbreaks in temperate regions [10–14], while contact and mobility patterns contribute to the seeding of new outbreaks and geographic spread [10,15–19]. A principal requirement for the recurrence of epidemics is a sufficient and continuous source of susceptible individuals, which is determined by the degree of cross-immunity between the surface antigens of currently circulating viruses and functional antibodies elicited by prior infection or vaccination in a population.

Because mutations to the HA1 region of the HA protein are considered to drive the majority of antigenic drift [20,21], influenza virus genetic and antigenic surveillance have focused primarily on HA, and official influenza vaccine formulations prescribe the amount of HA [22]. Yet, evidence for the effect of HA drift on influenza epidemic dynamics remains conflicting. Theoretical and empirical studies have shown that HA drift between currently circulating viruses and the previous season’s viruses is expected to cause earlier, larger, more severe, or more synchronized epidemics; however, the majority of these studies were limited to the pre 2009 influenza pandemic period [6,17,23–28]. Information on HA evolution has been shown to improve forecasts of seasonal influenza dynamics in Israel and the United States [29,30], but recent research has also found that HA evolution is not predictive of epidemic size in Australia [31] or epidemic timing in the United States [16]. A caveat is that many of these studies used binary indicators to study seasonal antigenic change, defined as seasons in which circulating viruses were antigenically distinct from the vaccine reference strain [16,17,24,31,32]. This may obscure epidemiologically relevant patterns, as positive selection in HA and NA is both episodic and continuous [6,32–37]. Past research has also typically focused on serological and sequence-based measures of viral evolution in isolation, and the relative importance of these two approaches in predicting epidemic dynamics has not been systematically assessed. Further, to the best of our knowledge, the epidemiologic impact of NA evolution has not been explored.

There has been recent recognition of NA’s role in virus inhibiting antibodies and its potential as a vaccine target [38–40]. Though antibodies against NA do not prevent influenza infection, NA immunity attenuates the severity of infection by limiting viral replication [41–46], and NA-specific antibody titers are an independent correlate of protection in both field studies and human challenge trials [47–49]. Lastly, the phenomenon of interference between influenza A subtypes, modulated by immunity to conserved T-cell epitopes [50–52], has long been debated [53,54]. Interference effects are most pronounced during pandemic seasons, leading to troughs or even replacement of the resident subtype in some pandemics [55], but the contribution of heterosubtypic interference to annual dynamics is unclear [2,56–59].

Here, we link A(H3N2) virus evolutionary dynamics to epidemiologic surveillance data in the United States over the course of 22 influenza seasons prior to the coronavirus disease (COVID-19) pandemic, considering the full diversity of viruses circulating in this period. We analyze a variety of antigenic and genetic markers of HA and NA evolution against multiple indicators characterizing the epidemiology and disease burden of annual outbreaks. We find a signature of both HA and NA antigenic drift in surveillance data, with a more pronounced relationship in epitope change rather than the serology-based indicator, along with a major effect of subtype interference. Our study has implications for surveillance of evolutionary indicators that are most relevant for population impact and for prediction of influenza burden on inter-annual timeframes.

## Results

Our study focuses on the impact of A(H3N2) virus evolution on seasonal epidemics from seasons 1997–1998 to 2018–2019 in the US; whenever possible, we make use of regionally disaggregated indicators and analyses. We start by identifying multiple indicators of influenza evolution each season based on changes in HA and NA. Next, we compile influenza virus subtype-specific incidence time series for US Department of Health and Human Service (HHS) regions and estimate multiple indicators characterizing influenza A(H3N2) epidemic dynamics each season, including epidemic burden, severity, intensity, type/subtype dominance, timing, and the age distribution of cases. We then assess univariate relationships between indicators of evolution and epidemic characteristics. Lastly, we measure the relative importance of viral evolution, heterosubtypic interference, and prior immunity in predicting regional A(H3N2) epidemic dynamics, using multivariable regression models and random forest models.

### Indicators of influenza A(H3N2) evolution

**Figure 1.**
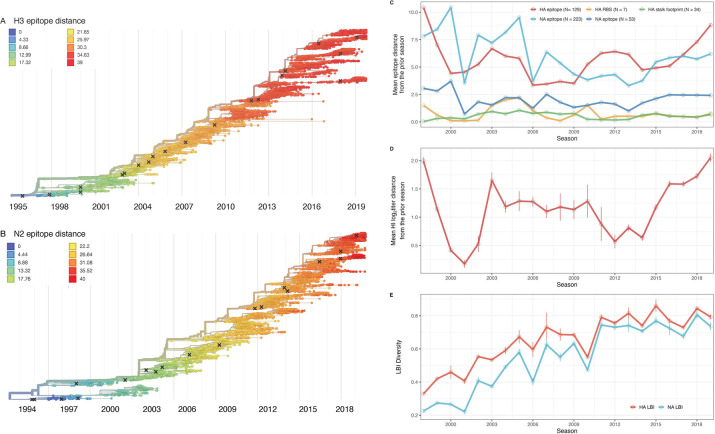
Antigenic and genetic evolution of seasonal influenza A(H3N2) viruses, 1997 – 2019. **A-B.** Temporal phylogenies of hemagglutinin (H3) and neuraminidase (N2) gene segments. Tip color denotes the Hamming distance from the root of the tree, based on the number of substitutions at epitope sites in H3 (N = 129 sites) and N2 (N = 223 sites). “X” marks indicate the phylogenetic positions of US recommended vaccine strains. **C-D.** Seasonal genetic and antigenic distances are the mean distance between A(H3N2) viruses circulating in the current season t versus the prior season (t – 1), measured by **C.** four sequence-based metrics (HA receptor binding site (RBS), HA stalk footprint, HA epitope, and NA epitope) and **D.** hemagglutination inhibition (HI) titer measurements. **E.** The Shannon entropy of H3 and N2 local branching index (LBI) values in each season. Vertical bars in **C, D,** and **E** and are 95% confidence intervals of seasonal estimates from five bootstrapped phylogenies.

We characterized seasonal patterns of genetic and antigenic evolution among A(H3N2) viruses circulating from 1997 to 2019, using HA and NA sequence data shared via the Global Initiative on Sharing Avian Influenza Data (GISAID) EpiFlu database [60] and ferret hemagglutination inhibition (HI) assay data shared by the WHO Global Influenza Surveillance and Response System (GISRS) Collaborating Centers in London, Melbourne, Atlanta, and Tokyo. Prior to constructing phylogenetic trees, we subsampled sequences to representative sets of 50 viruses per month, with preferential sampling for North American sequences. Although our study is US-focused, we used a global dataset because US-collected sequences and HI titers were sometimes sparse during the earlier seasons of the study. Time-resolved phylogenies of HA and NA genes are shown in Figure 1.

Our choice of evolutionary indicators builds on earlier studies that found hemagglutination inhibition (HI) phenotype or HA sequence data beneficial in forecasting seasonal influenza virus evolution [35,61–63] or annual epidemic dynamics [27,29,30] (Table 1). Historically, HI serological assays were considered the gold standard for measuring immune cross-reactivity between viruses, yet measurements are available for only a subset of viruses. To overcome this limitation, we used a computational approach that maps HI titer measurements onto the HA phylogenetic tree to infer antigenic phenotypes [35,63]. Importantly, this model infers the antigenicity of virus isolates that lack HI titer measurements, which comprise the majority of HA sequences in GISAID. Our sequence-based measures of drift counted substitutions at epitope sites in the globular head domains of HA and NA, identified through monoclonal antibody escape or protein crystal structure: 129 sites in HA epitope regions A to E [21,64–67], 7 sites adjacent to the HA receptor binding site (RBS) [68], and 223 or 53 sites in NA epitope regions A to C [34,69].

We included other indicators of viral fitness for HA and NA, including the number of substitutions at non-epitope sites (mutational load) [35,61] and the average rate of phylogenetic branching in a season (local branching index, LBI) [35,62]. We also calculated the Shannon entropy of LBI values, which considers the richness and relative abundances of viral clades with different growth rates. Lastly, we counted the number of substitutions at epitope sites in the HA stalk domain (stalk footprint distance) [70]. Although the majority of the antibody-mediated response to HA is directed to the immunodominant HA head, antibodies towards the highly conserved immunosubdominant stalk domain of HA are widely prevalent in older individuals, although at low levels [71–73]. We considered stalk footprint distance to be our “control” metric for drift, given the HA stalk evolves at a significantly slower rate than the HA head [70].

To measure antigenic distances between consecutive seasons, we calculated mean genetic distances at epitope sites or mean log_2_ titer distances from HI titer measurements (Figure 1), between viruses circulating in the current season *t* and the prior season *t-1* year (one season lag) or two prior seasons ago *t-2* years (two season lag). These time windows generated seasonal antigenic distances consistent with empirical and theoretical studies characterizing transitions between H3 or N2 antigenic clusters [6,32,35,55,62,74], with H3 epitope distance and HI log_2_ titer distance, at two-season lags, and N2 epitope distance, at one-season lags, capturing expected “jumps” in antigenic drift during key seasons that have been previously associated with major antigenic transitions [32], such as the seasons dominated by A/Sydney/5/1997-like strains (SY97) (1997–1998, 1998–1999, 1999–2000) and the 2003–2004 season dominated by A/Fujian/411/2002-like strains (FU02) (Figures S1–S2). Prior studies explicitly linking antigenic drift to epidemic size or severity also support a one-year [6] or two-year time window of drift [26,27]. Given that protective immunity wanes after 1–4 years, we would also expect these timeframes to return the greatest signal in epidemiological surveillance data.

We measured pairwise correlations between seasonal indicators of HA and NA evolution to assess their degree of concordance. As expected, we found moderate-to-strong associations between HA epitope distance and HI log_2_ titer distance and HA RBS distance and HI log_2_ titer distance (Figure S1–S3). Consistent with prior serological studies [39,75,76], epitope distances in HA and NA were not correlated (one-season lag: Spearman’s ρ = 0.25, P = 0.26; two-season lag: ρ = 0.15, P = 0.5; Figures S2–S4). Seasonal diversity of HA and NA LBI values was negatively correlated with NA epitope distance (Figure S3), suggesting that selective sweeps follow the emergence of drifted variants.

### Associations between A(H3N2) evolution and epidemic dynamics

We explored relationships between viral evolution and variation in A(H3N2) epidemic dynamics from seasons 1997–1998 to 2018–2019, excluding the 2009 A(H1N1) pandemic, using syndromic and virologic surveillance data collected by the US CDC and WHO.

**Figure 2.**
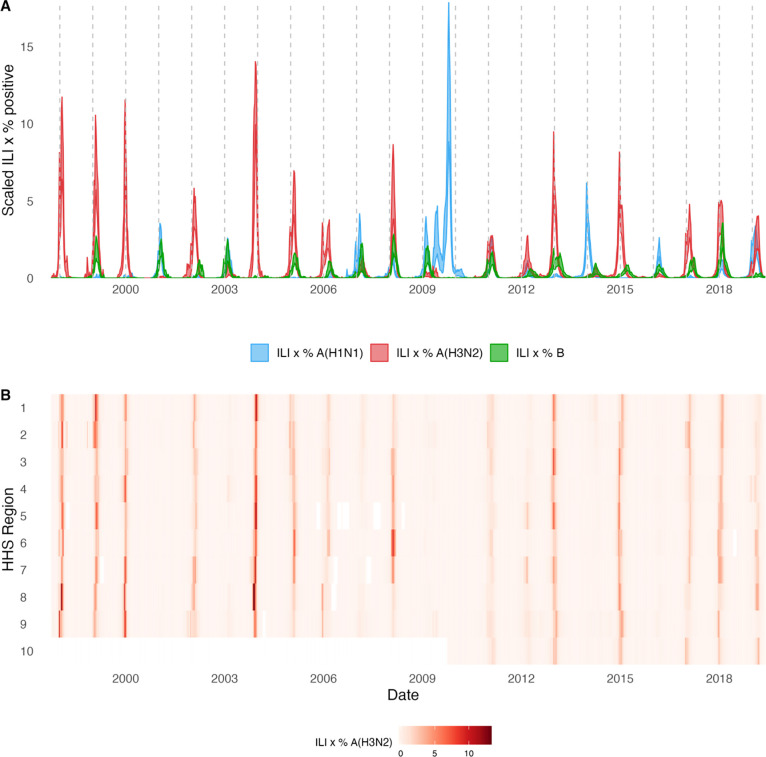
Annual influenza A(H3N2) epidemics in the United States, 1997 – 2019. **A.** Weekly incidence of influenza A(H3N2) (red), A(H1N1) (blue), and B (green) averaged across ten HHS regions (Region 1: Boston; Region 2: New York City; Region 3: Washington, DC; Region 4: Atlanta; Region 5: Chicago; Region 6: Dallas, Region 7: Kansas City; Region 8: Denver; Region 9: San Francisco; Region 10: Seattle). Time series are 95% confidence intervals of regional incidence estimates. Incidences are the proportion of influenza-like illness (ILI) visits among all outpatient visits, multiplied by the proportion of respiratory samples testing positive for each influenza type/subtype. Vertical dashed lines indicate January 1 of each year. **B.** Intensity of weekly influenza A(H3N2) incidence in ten HHS regions. White tiles indicate weeks when influenza-like-illness data or virological data were not reported. Weekly time series for A(H1N1) and B are in Figure S5.

We estimated weekly incidences of influenza A(H3N2), A(H1N1), and B in 10 HHS regions by multiplying the influenza-like illness (ILI) rate – the proportion of outpatient encounters for ILI, weighted by regional population size – by the regional proportion of respiratory samples testing positive for each influenza type/subtype (percent positive) [57,77]. We combined pre-2009 seasonal A(H1N1) viruses and A(H1N1)pdm09 viruses as A(H1N1) and the Victoria and Yamagata lineages of influenza B viruses as influenza B. Weekly incidences of influenza A(H3N2), A(H1N1), and type B, averaged across the 10 HHS regions, are shown in Figure 2. Weekly regional incidences, which show variability in the timing and intensity of annual epidemics, are shown in Figure 2 and Figure S5. Based on these incidence time series, we measured indicators of epidemic burden, intensity, severity, subtype dominance, timing, and age-specific patterns during each non-pandemic season and assessed their univariate relationships with each indicator of HA and NA evolution, which we describe in turn below. Seasonal characteristics of A(H3N2) epidemic dynamics were based on epidemic size, defined as the cumulative weekly incidence; peak incidence, defined as the maximum weekly incidence; excess mortality attributable to A(H3N2), an indicator of epidemic severity; transmissibility, defined as the maximum time-varying effective reproductive number, effective Rt; and epidemic intensity, defined as the inverse Shannon entropy of the weekly incidence distribution (i.e., the sharpness of the epidemic curve). See methods and Table 2 for details on all epidemic metrics and Figure S6 for pairwise correlations between metrics.

**Figure 3.**
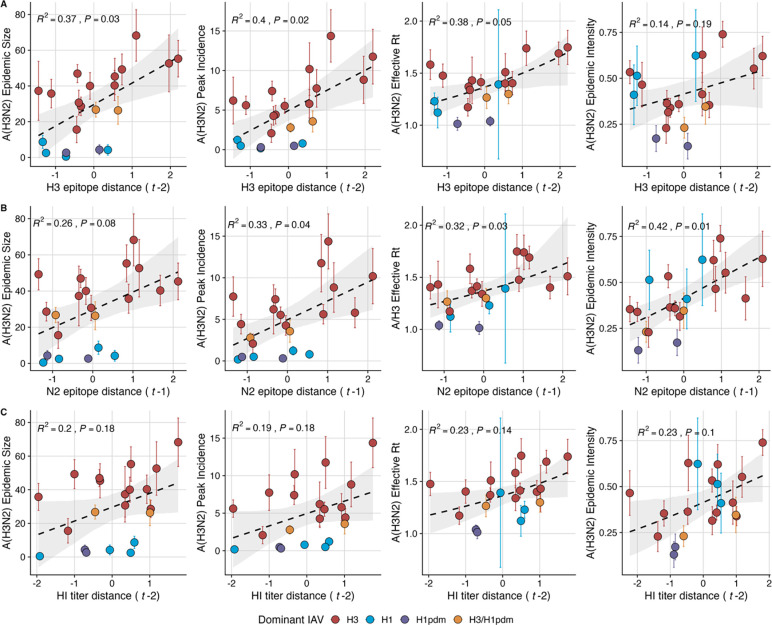
A(H3N2) antigenic drift correlates with larger, more intense annual epidemics. A(H3N2) epidemic size, peak incidence, epidemic intensity, and transmissibility (effective reproduction number, R_t_) increase with antigenic drift, measured by **A.** hemagglutinin (H3) epitope distance, and **B.** neuraminidase (N2) epitope distance, and **C.** hemagglutination inhibition (HI) log_2_ titer distance. Seasonal antigenic drift is the mean titer distance or epitope distance between viruses circulating in the current season t versus the prior season (t – 1) or two prior seasons (t – 2). Distances are scaled to aid in direct comparison of evolutionary indicators. Point color indicates the dominant influenza A virus (IAV) subtype based on CDC influenza season summary reports (red: A(H3N2), blue: A(H1N1), purple: A(H1N1)pdm09, orange: A(H3N2)/A(H1N1)pdm09 co-dominant), and vertical bands are 95% confidence intervals of regional estimates. Seasonal mean A(H3N2) epidemic metric values were fit as a function of antigenic or genetic distance using LMs (epidemic size, peak incidence), Gaussian GLMs (effective Rt: inverse link), or Beta GLMs (epidemic intensity) with 1000 bootstrap resamples.

Two sequence-based measures based on broad sets of epitope sites exhibited stronger relationships with seasonal epidemic burden and transmissibility than the serology-based measure, HI log_2_ titer distance. Both H3 epitope distance (*t* – 2) and N2 epitope distance (*t* – 1) correlated with increased epidemic size (linear models, LMs: H3, adjusted R^2^ = 0.37, P = 0.03; N2: R^2^ = 0.26, P = 0.08) and peak incidence (LMs, H3: R^2^ = 0.4, P = 0.02; N2: R^2^ = 0.33, P = 0.04) and higher effective Rt (generalized linear models, GLMs: H3, R^2^ = 0.38, P = 0.05; N2, R^2^ = 0.32, P = 0.03) (regression results: Figure 3, Spearman’s correlations: Figure S7). HI log_2_ titer distance (*t* – 2) exhibited positive but non-significant associations with different measures of epidemic impact (Figure 3, Figure S7). Seasonal diversity in the growth rates of circulating lineages in the current *t* or prior season (*t* – 1) had strong negative correlations with effective Rt (GLMs, H3 (*t* – 1): R^2^ = 0.49, P = 0.009; N2, *t*: R^2^ = 0.46, P = 0.006) and epidemic intensity (Beta GLMs, H3 (*t* – 1): R^2^ = 0.45, P = 0.003; N2, *t*: R^2^ = 0.51, P = 0.001) (Figures S7–S8). Seasonal mean LBI exhibited similar but slightly weaker correlations with effective Rt and epidemic intensity. Pneumonia and influenza excess mortality attributable to A(H3N2) also increased with H3 epitope distance, though this relationship was not statistically significant (Figure S9). The remaining indicators of viral evolution, including H3 and N2 non-epitope distance (mutational load), H3 RBS distance, and H3 stalk footprint distance had weak, non-significant correlations with the different measures of epidemic impact (Figure S7).

**Figure 4.**
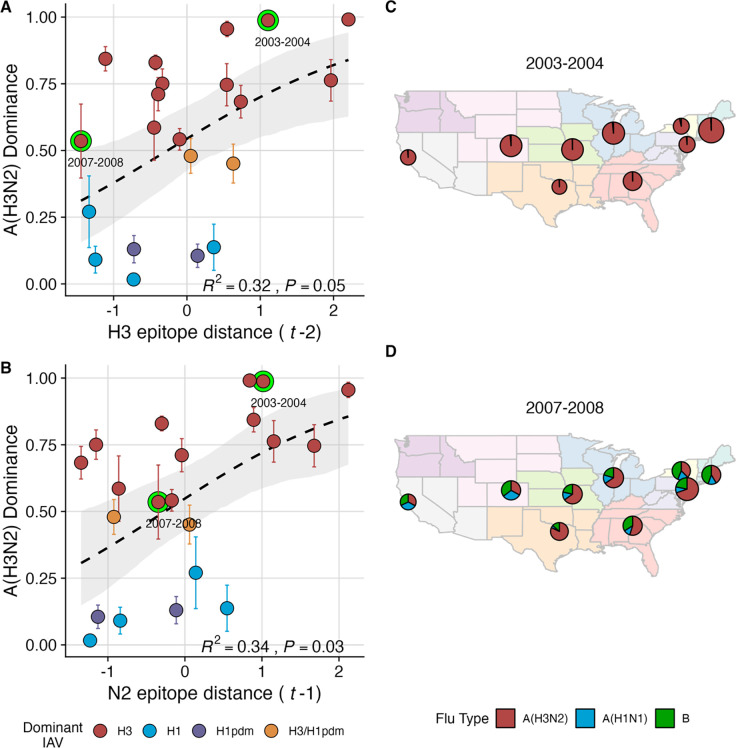
The proportion of influenza positive samples typed as A(H3N2) increases with antigenic drift. **A-B.** Seasonal A(H3N2) subtype dominance increases with H3 and N2 epitope distance. Seasonal epitope distance is the mean epitope distance between viruses circulating in the current season *t* versus the prior season (*t* – 1) or two prior seasons (*t* – 2). Distances were scaled to aid in direct comparison of evolutionary indicators. Point color indicates the dominant influenza A virus (IAV) subtype based on CDC influenza season summary reports (red: A(H3N2), blue: A(H1N1), purple: A(H1N1)pdm09, orange: A(H3N2)/A(H1N1)pdm09 co-dominant), and vertical bands are 95% confidence intervals of regional estimates. Seasonal mean A(H3N2) dominance was fit as a function of H3 or N2 epitope distance using Beta GLMs with 1000 bootstrap resamples. **C-D.** Regional patterns of influenza type and subtype incidence during two seasons when A(H3N2) was nationally dominant. **C.** Widespread A(H3N2) dominance during 2003–2004 after the emergence of a novel antigenic cluster, FU02 (A/Fujian/411/2002-like strains). **D.** Spatial heterogeneity in subtype circulation during 2007–2008, a season with low A(H3N2) antigenic novelty relative to the prior season. Pie charts represent the proportion of influenza positive samples typed as A(H3N2) (red), A(H1N1) (blue), or B (green) in each HHS region. Data for Region 10 (purple) were not available for seasons prior to 2009. The sizes of regional pie charts are proportional to the total number of influenza positive samples.

We explored whether evolutionary changes in A(H3N2) may predispose this subtype to dominate influenza virus circulation in a given season. A(H3N2) subtype dominance – the proportion of influenza positive samples typed as A(H3N2) – increased with H3 epitope distance (*t* – 2) and N2 epitope distance (*t* – 1) (Beta GLMs, H3: R^2^ = 0.32, P = 0.05; N2: R^2^ = 0.34, P = 0.03; Figure 4, Figure S7). Figure 4 illustrates this relationship at the regional level across two seasons in which A(H3N2) was nationally dominant, but where antigenic change differed. In 2003–2004, we observed widespread dominance of A(H3N2) viruses after the emergence of the novel antigenic cluster, FU02 (A/Fujian/411/2002-like strains). In contrast, there was substantial regional heterogeneity in subtype circulation during 2007–2008, a season in which A(H3N2) viruses were antigenically similar to those from the previous season. Patterns in type/subtype circulation across all influenza seasons in our study period are shown in Figure S10. As observed for the 2003–2004 season, widespread A(H3N2) dominance tends to coincide with major antigenic transitions (e.g., A/Sydney/5/1997 (SY97) seasons, 1997–1998 to 1999–2000; A/California/7/2004 (CA04) season, 2004–2005), though this was not universally the case (e.g., A/Perth/16/2009 (PE09) season, 2010–2011).

Next, we tested for associations between A(H3N2) evolution and epidemic timing, including onset week, defined as the winter changepoint in incidence [16], and peak week, defined as the first week of maximum incidence; spatiotemporal synchrony, measured as the variation (standard deviation, s.d.) in regional onset and peak timing; and epidemic speed, including seasonal duration and the number of weeks from onset to peak (Table 2, Figure S11). Seasonal duration increased with H3 or N2 LBI diversity in the current *t* or prior season (*t* – 1) (Gamma GLMs, H3, *t*: R^2^ = 0.6; P = 0.001; N2, *t*: R^2^ = 0.6; P = 0.002; Figures S11–S12), while the number of days from epidemic onset to peak shortened with increasing N2 epitope distance (*t* – 1) (Gamma GLM, R^2^ = 0.31, P = 0.04; Figure S11, Figure S13). Onset and peak timing tended to be earlier in seasons with increased H3 and N2 antigenic novelty, but correlations were not statistically significant (Figure S14). A(H3N2) evolution did not correlate with the degree of spatiotemporal synchrony across HHS regions.

Lastly, we considered the effects of antigenic change on the age distribution of outpatient ILI cases, with the expectation that the proportion of cases in children would decrease in seasons with greater antigenic novelty, due to drifted variants’ increased ability to infect more immunologically experienced adults [7,78]. Consistent with this hypothesis, N2 epitope distance from prior seasons was negatively correlated with the fraction of cases in children aged < 5 years (LMs, one-season lag: R^2^ = 0.29, P = 0.1; two-season lag: R^2^ = 0.59, P = 0.003) and individuals aged 5–24 years (one-season lag: R^2^ = 0.38, P = 0.04; two-season lag: R^2^ = 0.17, P = 0.18) and negatively correlated with the fraction of cases in adults aged 25–64 years (one-season lag: R^2^ = 0.36, P = 0.05; two-season lag: R^2^ = 0.49, P = 0.01) and ≥65 years (one-season lag: R^2^ = 0.39, P = 0.01; two-season lag: R^2^ = 0.33, P = 0.05) (Figures S15–S16). As observed in Gostic et al. [78], H3 epitope distance (*t* – 2) had negative but non-significant associations with the fraction of cases in children and positive but non-significant associations with the fraction of cases in adult age groups (Figures S15–S16).

### Effects of heterosubtypic viral interference on A(H3N2) epidemic burden and timing

**Figure 5.**
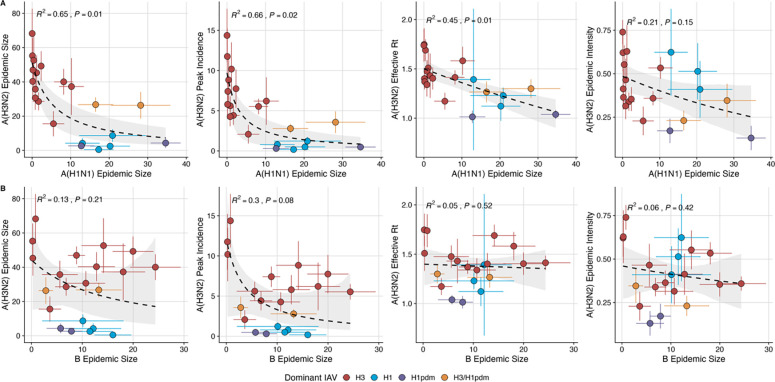
The effects of influenza A(H1N1) and B epidemic size on A(H3N2) epidemic burden. **A.** Influenza A(H1N1) epidemic size negatively correlates with A(H3N2) epidemic size, peak incidence, transmissibility (effective reproduction number, R_t_), and epidemic intensity. **B.** Influenza B epidemic size does not significantly correlate with A(H3N2) epidemic metrics. Point color indicates the dominant influenza A virus (IAV) subtype based on CDC influenza season summary reports (red: A(H3N2), blue: A(H1N1), purple: A(H1N1)pdm09, orange: A(H3N2)/A(H1N1)pdm09 co-dominant), and vertical and horizontal bands are 95% confidence intervals of regional estimates. Seasonal mean A(H3N2) epidemic metrics were fit as a function of mean A(H1N1) or B epidemic size using Gaussian GLMs (inverse link: epidemic size, peak incidence; log link: effective Rt) or Beta GLMs (epidemic intensity) with 1000 bootstrap resamples.

We investigated the effects of influenza type/subtype interference – proxied by influenza A(H1N1) and B epidemic size – on A(H3N2) incidence during annual outbreaks. Across the entire study period, we observed moderate-to-strong, non-linear relationships between A(H1N1) epidemic size and A(H3N2) epidemic size (GLM, R^2^ = 0.65, P = 0.01), peak incidence (R^2^ = 0.66, P = 0.02), and excess mortality (all age groups and ≥ 65 years, R^2^ = 0.57, P = 0.01) (Figure 5, Figure S17), wherein A(H3N2) epidemic burden and excess mortality decreased as A(H1N1) incidence increased. A(H1N1) epidemic size was also significantly correlated with A(H3N2) effective Rt, exhibiting a negative, approximately linear relationship (GLM, R^2^ = 0.45, P = 0.01) (Figure 5). A(H3N2) epidemic intensity was negatively associated with A(H1N1) epidemic size, but this relationship was not statistically significant (Beta GLM, R^2^ = 0.21, P = 0.15). Influenza B epidemic size was not significantly correlated with any A(H3N2) epidemic metrics (Figure 5, Figure S17).

The internal gene segments NS, M, NP, PA, and PB2 of A(H3N2) viruses and pre-2009 seasonal A(H1N1) viruses share a common ancestor [79] whereas A(H1N1)pdm09 viruses have a combination of gene segments derived from swine and avian reservoirs that were not reported prior to the 2009 pandemic [80,81]. Because pre-2009 seasonal A(H1N1) viruses and A(H3N2) are more closely related, seasonal A(H1N1) viruses may limit the circulation of A(H3N2) viruses to a greater extent than A(H1N1)pdm09 viruses. As a sensitivity analysis, we measured correlations between A(H1N1) incidence and A(H3N2) epidemic metrics separately for pre- and post-2009 pandemic time periods. Relationships between different A(H3N2) epidemic metrics and A(H1N1) epidemic size were broadly similar for both periods, with slightly stronger correlations observed during the pre-2009 period (Figure S18).

We compared A(H3N2) epidemic timing across A(H3N2) and A(H1N1) dominant seasons, which we defined as when ≥70% of influenza A positive samples are typed as A(H3N2) or A(H1N1)/A(H1N1)pdm09, respectively. We applied a strict threshold for subtype dominance because seasons with < 70% samples of one IAV subtype tended to have greater geographic heterogeneity in circulation, resulting in regions with dominant subtypes that were not nationally dominant. A(H3N2) epidemic onsets and peaks occurred, on average, three weeks earlier in A(H3N2) dominant seasons (Wilcoxon test, P < 0.0001). In A(H1N1) dominant seasons, regional A(H3N2) epidemics exhibited greater heterogeneity in epidemic timing (onset s.d.: H3 dominant seasons, 12.4 weeks versus H1 dominant seasons, 16.3 weeks; peak s.d., H3 dominant seasons, 13.3 weeks versus H1 dominant seasons, 22.6 weeks; Wilcoxon tests, P < 0.0001) and were significantly shorter in duration compared to A(H3N2) dominant seasons (median duration: H3 dominant seasons, 29 weeks versus H1 dominant seasons, 21 weeks; Wilcoxon test, P < 0.0001).

We applied a wavelet approach [82] to weekly time series of type/subtype-specific incidences to measure more fine-scale differences in the relative timing of type/subtype circulation (Figure S19). A(H3N2) incidence preceded A(H1N1) incidence during most seasons prior to 2009 and during the two seasons in which A(H1N1)pdm09 was dominant, potentially because A(H3N2) viruses are more globally prevalent and migrate between regions more frequently than A(H1N1) viruses [7]. There was not a clear relationship between the direction of seasonal phase lags and A(H1N1) epidemic size (LM, R^2^ = 0.23, P = 0.1; Figure S19). A(H3N2) incidence led influenza B incidence in all influenza seasons (positive phase lag), irrespective of influenza B epidemic size (LM, R^2^ = 0.05, P = 0.5; Figure S19).

### The relative impacts of viral evolution, heterosubtypic interference, and prior immunity on A(H3N2) epidemic dynamics

**Figure 6.**
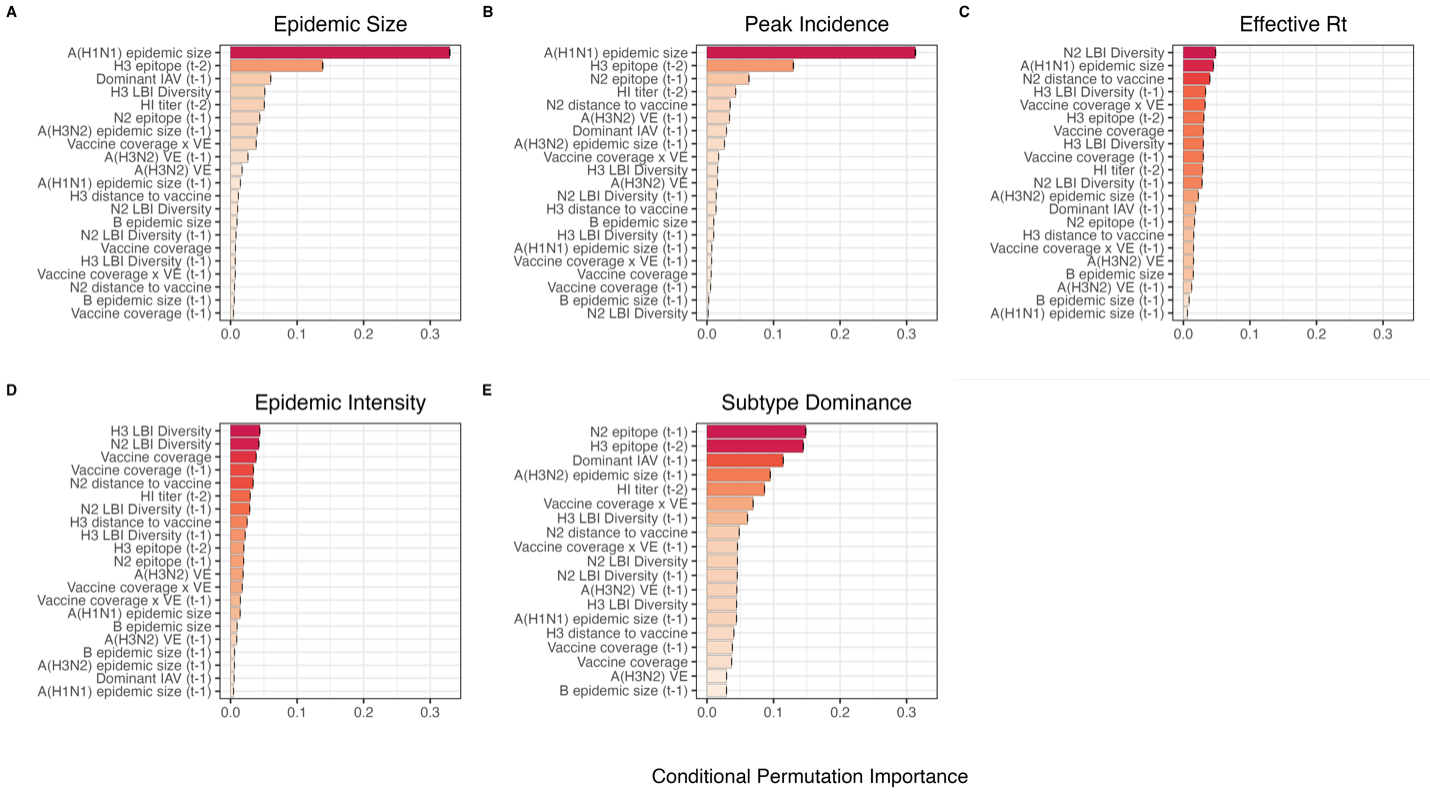
Variable importance rankings from conditional inference random forest models predicting A(H3N2) epidemic dynamics. Ranking of variables in predicting regional A(H3N2) **A.** epidemic size, **B.** peak incidence, **C.** effective reproduction number, Rt, **D.** epidemic intensity, and **E.** subtype dominance. Each forest was created by generating 3,000 regression trees from a repeated leave-one-season-out cross-validated sample of the data. Variables are ranked by their conditional permutation importance, with differences in prediction accuracy scaled by the total (null model) error. Black error bars are 95% confidence intervals of conditional permutation scores. Abbreviations: HI titer = hemagglutination inhibition log_2_ titer distance, t - 1 = one-season lag, t - 2 = two-season lag, LBI = local branching index, peak = peak incidence, distance to vaccine = epitope distance between currently circulating strains and the recommended vaccine strain, VE = vaccine effectiveness.

We implemented conditional inference random forest models to assess the relative importance of viral evolution, type/subtype co-circulation, prior population immunity, and vaccine-related parameters in predicting regional A(H3N2) epidemic metrics (Figure 6). We limited viral evolutionary indicators to H3 epitope distance (*t* – 2), N2 epitope distance (*t* – 1), HI log_2_ titer distance (*t* – 2), and H3 and N2 LBI diversity in the current and prior season, due to weaker or non-significant correlations between the other evolutionary metrics and epidemic burden (Figure S7). To account for potential type or subtype interference, we included A(H1N1) epidemic size (A(H1N1) or A(H1N1)pdm09) and B epidemic size in the current and prior season and the dominant IAV subtype in the prior season. We included A(H3N2) epidemic size in the prior season as a proxy of natural prior immunity to A(H3N2). To account for vaccine-induced immunity, we considered four categories of predictors and included estimates for the current and prior seasons: seasonal vaccination coverage among adults (18–49 years coverage × ≥ 65 years coverage), adjusted A(H3N2) vaccine effectiveness (VE), a combined metric of vaccination coverage and A(H3N2) VE (18–49 years coverage × ≥ 65 years coverage × VE), and H3 and N2 epitope distance between currently circulating strains and the US vaccine reference strain. We could not include a predictor for vaccination coverage in children or consider clade-specific VE estimates, because data were not available for most seasons in our study. We did not predict excess mortality attributable to A(H3N2), due to data limitation (one national estimate per season) and omitted models predicting epidemic timing, due to weak or non-significant correlations between timing-related measures and most indicators of viral evolution (Figure S11). Lastly, we could not separate our analysis into pre- and post-2009 pandemic periods due to small sample sizes.

Based on variable importance scores, A(H1N1) epidemic size in the current season was the most informative predictor of A(H3N2) epidemic size and peak incidence, followed by H3 epitope distance, and the dominant IAV subtype in the previous season or N2 epitope distance (Figure 6). For A(H3N2) subtype dominance, the highest ranked predictors were H3 epitope distance, N2 epitope distance, and the dominant IAV subtype in the previous season (Figure 6). We note that we did not include A(H1N1) epidemic size as a predictor in this model, due to its confounding with the target variable. For models of A(H3N2) effective Rt and epidemic intensity, we observed less discernable differences in variable importance scores across the set of candidate predictors (Figure 6). For the model of effective Rt, N2 LBI diversity in the current season, A(H1N1) epidemic size in the current season, and N2 epitope distance between circulating strains and the vaccine strain were the highest ranked variables, while the most important predictors of epidemic intensity were H3 and N2 LBI diversity in the current season and adult vaccination coverage in the current and prior season. Variable importance rankings from LASSO (least absolute shrinkage and selection operator) regression models were qualitatively similar to those from random forest models, with A(H1N1) epidemic size in the current season, H3 and N2 epitope distance, and the dominant IAV subtype in the prior season consistently retained across the best-tuned models of epidemic size, peak incidence, and subtype dominance (Figure S20). Vaccine-related parameters and H3 antigenic drift (either H3 epitope distance or HI log_2_ titer distance) were retained in the best-tuned LASSO models of effective Rt and epidemic intensity (Figure S20).

**Figure 7.**
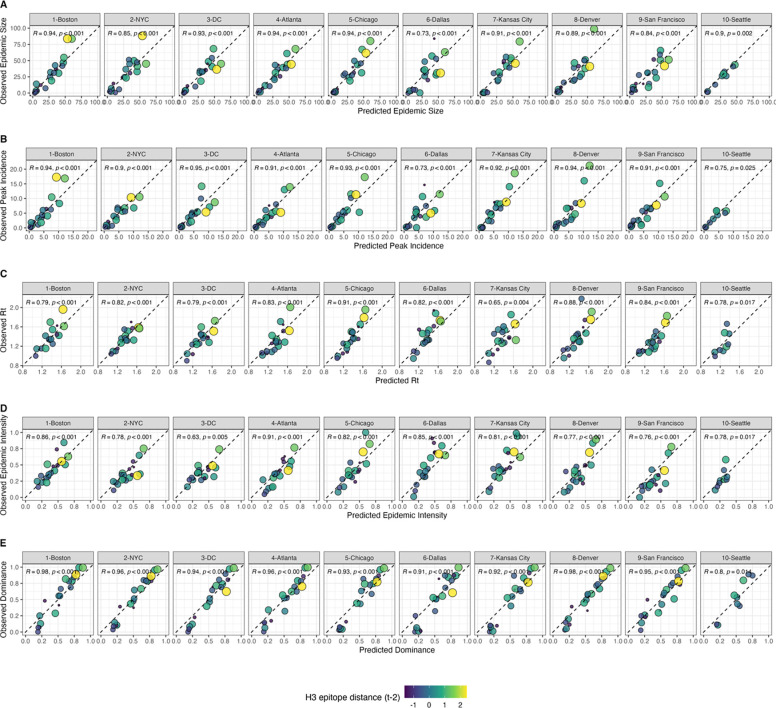
Observed versus predicted values of seasonal region-specific A(H3N2) A. epidemic size, B. peak incidence, C. effective reproduction number, Rt, D. epidemic intensity, and E. subtype dominance from conditional random forest models. Results are facetted by HHS region and epidemic metric. Point color and size corresponds to the degree of hemagglutinin (H3) epitope distance in viruses circulating in season *t* versus viruses circulating two seasons ago (*t* – 2). Large, yellow points indicate seasons with high antigenic novelty, and small blue points indicate seasons with low antigenic novelty. Regional Spearman’s correlation coefficients and associated P-values are in the top left section of each facet.

We measured correlations between observed values and model-predicted values at the HHS region level. Among our various epidemic metrics, random forest models produced the most accurate predictions of A(H3N2) subtype dominance (*ρ* = 0.94, regional range = 0.8 – 0.98), peak incidence (Spearman’s *ρ* = 0.91, regional range = 0.73 – 0.95), and epidemic size (*ρ* = 0.9, regional range = 0.73 – 0.94), while predictions of effective Rt and epidemic intensity were less accurate (*ρ* = 0.8, regional range = 0.65 – 0.91; *ρ* = 0.78, regional range = 0.63 – 0.91, respectively) (Figure 7). Random forest models tended to underpredict most epidemic targets in seasons with substantial H3 antigenic transitions, in particular the SY97 cluster seasons (1998–1999, 1999–2000) and the FU02 cluster season (2003–2004) (Figure 7).

For epidemic size and peak incidence, seasonal predictive error – root-mean-square error (RMSE) across all regional predictions in a season – increased with H3 epitope distance (size, Spearman’s *ρ* = 0.51, P = 0.02; peak, *ρ* = 0.61, P = 0.007) and N2 epitope distance (size, *ρ* = 0.43, P = 0.06; peak, *ρ* = 0.46, P = 0.04). For models of epidemic intensity, seasonal RMSE increased with N2 epitope distance (*ρ* = 0.62, P = 0.006) but not H3 epitope distance (*ρ* = 0.07, P = 0.8) (Figures S21–S22). The RMSE of effective Rt and subtype dominance predictions were not significantly correlated with H3 or N2 epitope distance (Figures S21–22).

To further refine our set of informative predictors, we performed multivariable regression with the top 10 ranked predictors from each random forest model and used Bayesian Information Criterion (BIC) to select the best fit model for each epidemic metric, allowing each metric’s regression model to include up to three independent variables. This additional step of variable selection demonstrated that models with few predictors fit the observed data relatively well (epidemic size, adjusted R^2^ = 0.69; peak incidence, adj. R^2^ = 0.63; effective Rt, adj. R^2^ = 0.65; epidemic intensity, adj. R^2^ = 0.75), except for subtype dominance (adj. R^2^ = 0.48) (Table 3). The set of variables retained after model selection were similar to those with high importance rankings in random forest models and LASSO regression models, with the exception that HI log_2_ titer distance, rather than H3 epitope distance, was included in the minimal models of effective Rt and epidemic intensity.

## Discussion

Antigenic drift between currently circulating influenza viruses and the previous season’s viruses is expected to confer increased viral fitness, leading to earlier, larger, or more severe epidemics. However, prior evidence for the impact of antigenic drift on seasonal influenza outbreaks is mixed. Here, we systematically compare experimental and sequence-based measures of A(H3N2) evolution in predicting regional epidemic dynamics in the United States across 22 seasons, from 1997 to 2019. We also consider the effects of other co-circulating influenza viruses, prior immunity, and vaccine-related parameters, such as coverage and effectiveness, on A(H3N2) incidence. Our findings indicate that evolution in both major surface proteins – hemagglutinin (HA) and neuraminidase (NA) – contributes to variability in epidemic magnitude across seasons, though viral fitness appears to be secondary to subtype interference in shaping annual outbreaks.

The first question of this study sought to determine which metrics of viral fitness have the strongest relationships with A(H3N2) epidemic burden and timing. Among our set of candidate evolutionary predictors, genetic distances based on broad sets of epitope sites (HA = 129 sites; NA = 223 epitope sites) had the strongest, most consistent associations with A(H3N2) epidemic size, transmission rate, severity, subtype dominance, and age-specific patterns. Increased epitope distance in both H3 and N2 correlated with larger epidemics and increased transmissibility, with univariate analyses finding H3 distance more strongly correlated with epidemic size, peak incidence, transmissibility, and excess mortality, and N2 distance more strongly correlated with epidemic intensity (i.e., the “sharpness” of the epidemic curve) and subtype dominance patterns. However, we note that minor differences in correlative strength between H3 and N2 epitope distance are not necessarily biologically relevant and could be attributed to noise in epidemiological or virological data or the limited number of influenza seasons in our study. The fraction of ILI cases in children relative to adults was negatively correlated with N2 epitope distance, consistent with the expectation that cases are more restricted to immunologically naïve children in seasons with low antigenic novelty [7,78]. Regarding epidemic timing, the number of days from epidemic onset to peak (a proxy for epidemic speed) decreased with N2 epitope distance, but other measures of epidemic timing, such as peak week, onset week, and spatiotemporal synchrony across HHS regions, were not significantly correlated with H3 or N2 antigenic change.

The local branching index (LBI) is traditionally used to predict the success of individual clades, with a high LBI value indicating high viral fitness [35,62]. In our epidemiological analysis, low diversity of H3 or N2 LBI values, in the current or prior season, correlated with greater epidemic intensity, higher transmission rates, and shorter seasonal duration. This outcome suggests that low LBI diversity is indicative of a rapid selective sweep by one successful clade and that high LBI diversity is indicative of multiple co-circulating clades with variable seeding times over the course of an epidemic. A caveat is that LBI estimation is more sensitive to sequence sub-sampling schemes than strain-level measures. If an epidemic is very short and intense (e.g., 1–2 months), a phylogenetic tree with our sub-sampling scheme (50 sequences per month) may not incorporate enough sequences to capture the true diversity of LBI values in that season.

Positive associations between H3 antigenic drift and population-level epidemic burden are consistent with previous observations from theoretical models [25,26,83]. For example, phylodynamic models of punctuated antigenic evolution have reproduced key features of A(H3N2) phylogenetic patterns and case dynamics, such as the sequential replacement of antigenic clusters, the limited standing diversity in HA after a cluster transition, and higher incidence and attack rates in cluster transition years [25,26,83]. Our results also corroborate empirical analyses of surveillance data [6,27,28,66] and forecasting models of annual epidemics [29,30] that found direct, quantitative links between HA antigenic novelty and the number of influenza cases or deaths in a season. Moving beyond the paradigm of antigenic clusters, Wolf et al., 2010 and Bedford et al., 2014 demonstrated that smaller, year-to-year changes in H3 antigenic drift also correlate with seasonal severity and incidence [6,27]. A more recent study did not detect an association between antigenic drift and city-level epidemic size in Australia [31], though the authors used a binary indicator to signify seasons with major HA antigenic transitions and did not consider smaller, more gradual changes in antigenicity. While Lam and colleagues did not observe a consistent effect of antigenic change on epidemic magnitude, they found a negative relationship between the cumulative prior incidence of an antigenic variant and its probability of successful epidemic initiation in a city [31].

We did not observe a clear relationship between H3 receptor binding site (RBS) distance and epidemic burden, even though single substitutions at these seven amino acid positions are implicated in major antigenic transitions [68,84]. The outperformance of the RBS distance metric by a broader set of epitope sites could be attributed to the tempo of antigenic cluster changes. A(H3N2) viruses are characterized by both continuous and punctuated antigenic evolution, with transitions between antigenic clusters occurring every 2 to 8 years [6,26,32,33,36,37,67,68,85]. Counting substitutions at only a few sites may fail to capture more modest, gradual changes in antigenicity that are on a time scale congruent with annual outbreaks. Further, a broader set of epitope sites may better capture the epistatic interactions that underpin antigenic change in HA [86]. Although the 7 RBS sites were responsible for the majority of antigenic phenotype in Koel et al.’s experimental study [68], their findings do not necessarily contradict studies that found broader sets of sites associated with antigenic change. Mutations at other epitope sites may collectively add to the decreased recognition of antibodies or affect viral fitness through alternate mechanisms (e.g., compensatory or permissive mutations) [26,32,36,62,68,86–88].

A key result from our study is the direct link between NA antigenic drift and A(H3N2) incidence patterns. Although HA and NA both contribute to antigenicity [20,89] and undergo similar rates of positive selection [34], we expected antigenic change in HA to exhibit stronger associations with seasonal incidence, given its immunodominance relative to NA [90]. H3 and N2 epitope distance were both moderately correlated with epidemic size, peak incidence, and subtype dominance patterns, but, except for subtype dominance, H3 epitope distance had higher variable importance rankings in random forest models and N2 epitope distance was not retained after post-hoc model selection of top ranked random forest features. However, N2 epitope distance but not H3 epitope distance was associated with faster epidemic speed and a greater fraction of ILI cases in adults relative to children. Antigenic changes in H3 and N2 were independent across the 22 seasons of our study, consistent with previous research [34,74,76]. Thus, the similar predictive performance of HA and NA epitope distance for some epidemic metrics does not necessarily stem from the coevolution of HA and NA.

HI log_2_ titer distance was positively correlated with different measures of epidemic impact yet underperformed in comparison to H3 and N2 epitope distances. This outcome was surprising given that we expected our method for generating titer distances to produce more realistic estimates of immune cross-protection between viruses than epitope-based measures. Our computational approach for inferring HI phenotype dynamically incorporates newer titer measurements and assigns antigenic weight to phylogenetic branches rather than fixed sequence positions [35,63], while our method for calculating epitope distance assumes that the contributions of specific sites to antigenic drift are constant through time, even though beneficial mutations previously observed at these sites are contingent on historical patterns of viral fitness and host immunity [26,35,62]. HI titer measurements have been more useful than epitope substitutions in predicting future A(H3N2) viral populations [35] and vaccine effectiveness [91], with the caveat that these targets are more proximate to viral evolution than epidemic dynamics.

HI titer measurements may be more immunologically relevant than epitope-based measures, yet several factors could explain why substitutions at epitope sites outperformed HI titer distances in epidemiological predictions. First, epitope distances may capture properties that affect viral fitness (and in turn outbreak intensity) but are unrelated to immune escape, such as intrinsic transmissibility, ability to replicate, or epistatic interactions. A second set of factors concern methodological issues associated with HI assays. The reference anti-sera for HI assays are routinely produced in ferrets recovering from their first influenza virus infection. Most humans are infected by different influenza virus strains over the course of their lifetimes, and one’s immune history influences the specificity of antibodies generated against drifted influenza virus strains [92–95]. Thus, human influenza virus antibodies, especially those of adults, have more heterogeneous specificities than anti-sera from immunologically naïve ferrets [92].

A related methodological issue is that HI assays disproportionately measure anti-HA antibodies that bind near the receptor binding site and, similar to the RBS distance metric, may capture only a partial view of the antigenic change occurring in the HA protein [31,78,96,97]. A recent study of longitudinal serological data found that HI titers are a good correlate of protective immunity for children, while time since infection is a better predictor of protection for adults [97]. This outcome is consistent with the concept of antigenic seniority, in which an individual’s first exposure to influenza virus during childhood leaves an immunological “imprint”, and exposure to new strains “back boosts” one’s antibody response to strains of the same subtype encountered earlier in life [78,98,99]. Ranjeva et al.’s study and others suggest that human influenza virus antibodies shift focus from the HA head to other more conserved epitopes as individuals age [78,96]. Given that HI assays primarily target epitopes adjacent to the RBS, HI assays using ferret or human serological data are not necessarily suitable for detecting the broader immune responses of adults. A third explanation for the underperformance of HI titers concerns measurement error. Recent A(H3N2) viruses have reduced binding efficiency in HI assays, which can skew estimates of immune cross-reactivity between viruses [100]. These combined factors could obfuscate the relationship between the antigenic phenotypes inferred from HI assays and population-level estimates of A(H3N2) incidence.

Novel antigenic variants are expected to have higher infectivity in immune populations, leading to earlier epidemics and more rapid geographic spread [19], but few studies have quantitatively tied antigenic drift to epidemic timing or geographic synchrony. Previous studies of pneumonia and influenza-associated mortality observed greater severity or geographic synchrony in seasons with major antigenic transitions [21,24]. A more recent Australian study of lab-confirmed cases also noted greater spatiotemporal synchrony during seasons in which novel H3 antigenic variants emerged, although their assessment was based on virus typing alone (i.e., influenza A or B) [17]. A subsequent Australian study with finer-resolution data on subtype incidence and variant circulation determined that more synchronous epidemics were not associated with drifted A(H3N2) strains [31], and a US-based analysis of ILI data also failed to detect a relationship between HA antigenic cluster transitions and geographic synchrony [16]. In our study, the earliest epidemics tended to occur in seasons with transitions between H3 antigenic clusters (e.g., the emergence of the FU02 cluster in 2003–2004) or vaccine mismatches (e.g., N2 mismatch in 1999–2000, H3 mismatch in 2014–2015) [32,74,101], but there was not a statistically significant correlation between antigenic change and earlier epidemic onsets or peaks. Regarding epidemic speed, the length of time from epidemic onset to peak decreased with N2 epitope distance but not H3 epitope distance. The relationship between antigenic drift and epidemic timing may be ambiguous because external seeding events or climatic factors, such as temperature and absolute humidity, are more important in driving influenza seasonality and the onsets of winter epidemics [7,10–14,16]. Alternatively, the resolution of our epidemiological surveillance data (HHS regions) may not be granular enough to detect a signature of antigenic drift in epidemic timing, though studies of city-level influenza dynamics were also unable to identify a clear relationship [16,31].

After exploring individual correlations between evolutionary indicators and annual epidemics, we considered the effects of influenza A(H1N1) incidence and B incidence on A(H3N2) virus circulation within a season. We detected strong negative associations between A(H1N1) incidence and A(H3N2) epidemic size, peak incidence, transmissibility, and excess mortality, consistent with previous animal, epidemiological, phylodynamic, and theoretical studies that found evidence for cross-immunity between IAV subtypes [53–55,57,59,102]. For example, individuals recently infected with seasonal influenza A viruses are less likely to become infected during subsequent pandemic waves [52,53,102–104], and the early circulation of one influenza virus type or subtype is associated with a reduced total incidence of the other type/subtypes within a season [31,57]. Due to the shared evolutionary history of their internal genes [79], pre-2009 seasonal A(H1N1) viruses may impact A(H3N2) virus circulation to a greater extent than A(H1N1)pdm09 viruses, which have a unique combination of genes that were not identified in animals or humans prior to 2009 [81,105]. We observed similar relationships between A(H3N2) epidemic metrics and A(H1N1) incidence during pre- and post-2009 pandemic seasons, with slightly stronger correlations observed during the pre-2009 period. However, given the small sample size (12 pre-2009 seasons and 9 post-2009 seasons), we cannot fully answer this question.

In our study, univariate correlations between A(H1N1) and A(H3N2) incidence were more pronounced than those observed between A(H3N2) incidence and evolutionary indicators, and A(H1N1) epidemic size was the highest ranked feature by random forest models predicting epidemic size and peak incidence. Consequently, interference between the two influenza A subtypes may be more impactful than viral evolution in determining the size of annual A(H3N2) outbreaks. Concerning epidemic timing, we did not detect a relationship between A(H3N2) antigenic change and the relative timing of A(H3N2) and A(H1N1) cases; specifically, A(H3N2) incidence did not consistently lead A(H1N1) incidence in seasons with greater H3 or N2 antigenic change. Overall, we did not find any indication that influenza B incidence affects A(H3N2) epidemic burden or timing, which is not unexpected, given that few T and B cell epitopes are shared between the two virus types [106].

Lastly, we used random forest models and multivariable linear regression models to assess the relative importance of viral evolution, prior population immunity, co-circulation of other influenza viruses, and vaccine-related parameters in predicting regional A(H3N2) epidemic dynamics. We chose conditional inference random forest models as our primary method of variable selection because several covariates were collinear, relationships between some predictors and target variables were nonlinear, and our goal was inferential rather than predictive. We performed leave-one-season-out cross-validation to tune each model, but, due to the limited number of seasons in our dataset, we were not able to test predictive performance on an independent test set. With the caveat that models were likely overfit to historical data, random forest models produced accurate predictions of regional epidemic size, peak incidence, and subtype dominance patterns, while predictions of epidemic intensity and transmission rates were less exact. The latter two measures could be more closely tied to climatic factors, the timing of influenza case importations from abroad, or mobility patterns [7,13,14,16] or they may be inherently more difficult to predict because their values are more constrained. Random forest models tended to underpredict epidemic burden in seasons with major antigenic transitions, particularly the SY97 seasons (1998–1999, 1999–2000) and the FU02 season (2003–2004), potentially because antigenic jumps of these magnitudes were infrequent during our 22-season study period. An additional step of post-hoc model selection demonstrated that models with only three covariates could also produce accurate fits to observed epidemiological data.

Our study is subject to several limitations, specifically regarding geographic resolution and data availability. First, our analysis is limited to one country with a temperate climate and its findings concerning interactions between A(H3N2), A(H1N1), and type B viruses may not be applicable to tropical or subtropical countries, which experience sporadic epidemics of all three viruses throughout the year [107]. Second, our measure of population-level influenza incidence is derived from regional CDC outpatient data because those data are publicly available starting with the 1997–1998 season. State level outpatient data are not available until after the 2009 A(H1N1) pandemic, and finer resolution data from electronic health records are accessible in theory but not in the public domain. Access to ILI cases aggregated at the state or city level, collected over the course of decades, would increase statistical power and enable us to add more location-specific variables to our analysis, such as climatic and environmental factors. A third limitation is that we measured influenza incidence by multiplying the rate of influenza-like illness by the percentage of tests positive for influenza, which does not completely eliminate the possibility of capturing the activity of other co-circulating respiratory pathogens [11]. Surveillance data based on more specific diagnosis codes would ensure the exclusion of patients with non-influenza respiratory conditions. Fourth, our data on the age distribution of influenza cases were derived from ILI encounters across four broad age groups and did not include test positivity status, virus type/subtype, or denominator information. Despite the coarseness of these data, we found statistically significant correlations in the expected directions between N2 antigenic change and the fraction of cases in children relative to adults. Lastly, a serological assay exists for NA, but NA titer measurements are not widely available because the assay is labor-intensive and inter-lab variability is high. Thus, we could not test the performance of NA antigenic phenotype in predicting epidemic dynamics.

Beginning in early 2020, non-pharmaceutical interventions (NPIs), including lockdowns, school closures, physical distancing, and masking, were implemented in the United States and globally to slow the spread of severe acute respiratory syndrome coronavirus 2 (SARS-CoV-2), the virus responsible for the COVID-19 pandemic. These mitigation measures disrupted the transmission of seasonal influenza viruses and other directly-transmitted respiratory viruses throughout 2020 and 2021 [108–113], and population immunity to influenza is expected to have decreased substantially during this period of low circulation [114,115]. COVID-19 NPIs relaxed during 2021 and 2022, and co-circulation of A(H3N2) and A(H1N1)pdm09 viruses in the United States resumed during the 2022–2023 influenza season. Our study concludes with the 2018–2019 season, and thus it is unclear whether our modeling approach would be useful in projecting seasonal burden during the post-pandemic period, without an additional component to account for COVID-19-related perturbations to influenza transmission. Further studies will need to determine whether ecological interactions between influenza viruses have changed or if the effects of viral evolution and subtype interference on seasonal outbreaks are different in the post-pandemic period.

In conclusion, relationships between A(H3N2) antigenic drift, epidemic impact, and age dynamics are moderate, with genetic distances based on broad sets of H3 and N2 epitope sites having greater predictive power than serology-based antigenic distances for the timeframe analyzed. Influenza epidemiological patterns are consistent with increased population susceptibility in seasons with high antigenic novelty, and our study is the first to link NA antigenic drift to epidemic burden, timing, and the age distribution of cases. It is well established that anti-HA and anti-NA antibodies are independent correlates of immunity [45,47–49,116–118], and the influenza research community has advocated for NA-based vaccines [39,119]. The connection between NA drift and seasonal incidence further highlights the importance of monitoring evolution in both HA and NA to inform vaccine strain selection and epidemic forecasting efforts. Although antigenic change in both HA and NA was correlated with epidemic dynamics, ecological interactions between influenza A subtypes appear to be more influential than viral evolution in determining the intensity of annual A(H3N2) epidemics. The aim of our study was to retrospectively assess the potential drivers of annual A(H3N2) epidemics, yet we cautiously suggest that one could project the size or intensity of future epidemics based on sequence data and A(H1N1)pdm09 incidence alone [27,57].

## Methods

Unless otherwise noted, data processing and statistical analyses were performed using R version 4.3.0.

### Influenza epidemic timing and burden

#### Influenza-like illness and virological surveillance data

We obtained weekly epidemiological and virological data for influenza seasons 1997–1998 to 2018–2019, at the U.S. HHS region level [120]. We defined influenza seasons as calendar week 40 in a given year to calendar week 20 in the following year, with the exception of the 2008–2009 season, which ended in 2009 week 16 due to the emergence of the A(H1N1)pdm09 virus [57].

We extracted syndromic surveillance data for the ten HHS regions from the U.S. Outpatient Influenza-like Illness Surveillance Network (ILINet) [120]. ILINet consists of approximately 3,200 sentinel outpatient healthcare providers throughout the United States that report the total number of consultations for any reason and the number of consultations for influenza-like illness (ILI) every week. ILI is defined as fever (temperature of 100°F [37.8°C] or greater) and a cough and/or a sore throat. The indicator is based on the weekly proportion of outpatient consultations for influenza-like illness and is available weighted or unweighted by regional population size. The number of ILI encounters by age group are also provided (0–4, 5–24, 25–64, and ≥65), but these data are not weighted by total encounters or population size.

Data on weekly influenza virus type and subtype circulation were obtained from the US CDC’s World Health Organization (WHO) Collaborating Center for Surveillance, Epidemiology and Control of Influenza [121]. We estimated the weekly number of respiratory samples testing positive for influenza A(H1N1), A(H1N1)pdm09, A(H3N2), or B at the HHS region level (see Supplementary Methods for details on data processing). We defined influenza A subtype dominance in each season based on the proportion of influenza A virus (IAV) positive samples typed as A(H3N2). We defined seasons as A(H3N2) or A(H1N1) or A(H1N1)pdm09 dominant when ≥70% of IAV positive samples were typed as one IAV subtype and co-dominant when one IAV subtype comprised 50–69% of IAV positive samples.

For each HHS region, we estimated weekly incidences of influenza A(H3N2), A(H1N1), and B by multiplying the percentage of influenza-like illness among outpatient visits, weighted by regional population, with the percentage of respiratory samples testing positive for a particular type/subtype [57,77]. We combined pre-2009 seasonal A(H1N1) and A(H1N1)pdm09 viruses as A(H1N1) and the Victoria and Yamagata lineages of influenza B as influenza B. ILI × percent positive (ILI^+^) is considered a robust estimate of influenza activity and has been used in multiple prior modeling studies [6,18,57,77,122]. We used linear interpolation to estimate missing values for time spans of up to four consecutive weeks.

The emergence of A(H1N1)pdm09 in 2009 altered influenza testing and reporting patterns. We adjusted weekly incidences for differences in reporting rates between the pre-2009 pandemic period – defined as 1997 week 40 to 2009 week 17 – and the post-pandemic period – defined as the weeks after 2010 week 33. For each region, we scaled pre-pandemic incidences by the ratio of mean weekly ILI^+^ (for all influenza type/subtypes combined) in the post-pandemic period to that of the pre-pandemic period. Incidences for HHS Region 10 were not adjusted for pre- and post-pandemic reporting because surveillance data for this region were not available before 2009. To account for differences in reporting rates across HHS regions, we next scaled each region’s type/subtype incidences by its mean weekly ILI^+^ for the entire study period. Scaled incidences were used in all downstream analyses of epidemic burden and timing.

#### Epidemic burden and timing

##### Epidemic burden:

We considered three complementary indicators of epidemic burden, separately for each influenza type/subtype, HHS region, and season. We defined *peak incidence* as the maximum weekly scaled incidence and *epidemic size* as the cumulative weekly scaled incidence. We also estimated *epidemic intensity* based on a method previously developed to study variation in the shape (i.e., sharpness) of influenza epidemics across US cities [123]. Epidemic intensity was based on the inverse Shannon entropy of the weekly incidence distribution. Epidemic intensity increases when incidence is more concentrated in particular weeks and decreases when incidence is more evenly spread across weeks.

Specifically, we defined the incidence distribution $p_{ij}$ as the fraction of influenza incidence in season j that occurred during week i in a given region, and epidemic intensity $v_{j}$ as the inverse of the Shannon entropy of the incidence distribution:

$$v_{j}={\left(-\sum_{i}\;\;p_{ij}ln\;p_{ij}\right)}^{-1}$$

Epidemic intensity values were normalized to fall between 0 and 1.

##### Transmission intensity:

For each region, we used the Epidemia R package to model annual A(H3N2) epidemics and to estimate time-varying (instantaneous) reproduction numbers, effective Rt [124,125](see Supplementary Methods for model details). Epidemia implements a semi-mechanistic Bayesian approach using the probabilistic programming language Stan [126].

To generate seasonal indicators of transmission intensity, we extracted posterior draws of daily Rt estimates for each region and season, calculated the median value for each day, and averaged daily median values by epidemic week. For each region and season, we averaged Rt estimates from the weeks spanning epidemic onset to epidemic peak (*initial Rt*) and averaged the two highest Rt estimates (*maximum Rt*). Initial Rt and maximum Rt produced qualitatively similar results in downstream analyses; we opted to report results for maximum Rt.

##### Excess pneumonia and influenza deaths attributable to A(H3N2):

To measure the epidemic severity each season, we obtained estimates of seasonal excess mortality attributable to influenza A(H3N2) from Hansen et al., 2022 [127]. Excess mortality is a measure of the mortality burden of a given pathogen in excess of a seasonally adjusted baseline, obtained by regressing weekly deaths from broad disease categories against indicators of influenza virus circulation. Hansen et al. used pneumonia and influenza (P&I) excess deaths, which is considered the most specific indicator of influenza burden [128]. Deaths with a mention of P&I (ICD 10: J00-J18) were aggregated by week and age group (<1, 1–4, 5–49, 50–64, and ≥65) for 1998–2018. Age-specific generalized linear models were fit to observed weekly P&I death rates, while accounting for influenza and respiratory syncytial virus (RSV) activity and seasonal and temporal trends. Hansen et al. estimated the weekly national number of excess A(H3N2)-associated deaths by subtracting the baseline death rate expected in the absence of A(H3N2) circulation (A(H3N2) model terms set to zero) from the observed P&I death rate. We summed the number of excess A(H3N2) deaths per 100,000 people from October to May to obtain seasonal age-specific estimates.

##### Epidemic onset and peak timing:

We estimated the regional onsets of A(H1N1), A(H1N1)pdm09, A(H3N2), and B epidemics each season by fitting piecewise linear models to subtype-specific incidence curves from week 30 to the first week of maximum incidence. We did not estimate epidemic onsets for regions with insufficient signal, which we defined as fewer than three weeks of consecutive incidence and/or greater than 30% of weeks with missing data in a particular season. The timing of the changepoint in incidence represents epidemic establishment (i.e., sustained transmission) rather the timing of influenza introduction or arrival [16]. We were able to estimate A(H3N2) onset timing for most seasons, except for three A(H1N1) dominant seasons: 2000–2001 (0 regions), 2002–2003 (3 regions), and 2009–2010 (0 regions). We defined epidemic peak timing as the first week of maximum incidence. To measure spatiotemporal synchrony, we calculated seasonal variation (standard deviation, s.d.) in regional onset and peak timing [19,27]. To measure the speed of viral spread, we calculated the number of days between onset and peak and seasonal duration (the number of weeks with non-zero incidence) for each region. As a sensitivity analysis, we used wavelets to estimate timing differences between A(H3N2), A(H1N1), A(H1N1)pdm09, and B epidemics (see Supplementary Methods).

##### Age patterns:

We calculated the seasonal proportion of ILI encounters in each age group (0–4 years, 5–24 years, 25–64 years, and ≥65 years). Data for more narrow age groups are available after 2009, but we chose these four categories to increase the number of seasons in our analysis.

#### Influenza vaccination coverage and A(H3N2) vaccine effectiveness

Influenza vaccination coverage and effectiveness vary between years and would be expected to affect the population impact of seasonal outbreaks, and in turn our epidemiologic indicators. We obtained seasonal estimates of national vaccination coverage for adults 18–49 years and adults ≥65 years from studies utilizing vaccination questionnaire data collected by the National Health Interview Survey [129–135]. We did not consider the effects of vaccination coverage in children, due to our inability to find published estimates for most influenza seasons in our study.

We obtained seasonal estimates of adjusted A(H3N2) vaccine effectiveness (VE) from 32 observational studies [136–167]. Most of these studies had case-control test-negative designs (N = 30) and took place in North America (N = 25) or Europe (N = 6). When possible, we limited VE estimates to those for healthy adults or general populations. When multiple VE studies were available for a given season, we calculated mean VE as the weighted average of m different VE point estimates:

$$\frac{\sum_{i=1}^{m}\;\;\delta_{VE_{i}}^{-1/2}VE_{i}}{\sum_{i=1}^{m}\;\;\delta_{VE_{i}}^{-1/2}}$$

Wherein $\delta_{VE}$ denotes the width of the 95% confidence interval (CI) for $VE_{i}$ [91].

The 95% CI for the weighted mean VE was calculated as:

$$\frac{1}{m}\sqrt{\sum_{i=1}^{m}\;\;{\left(\delta_{VE_{i}}\right)}^{2}}$$

### Correlations among epidemic metrics

We used Spearman’s correlation coefficients to measure pairwise relationships between A(H3N2) epidemiological indictors. We adjusted P-values for multiple testing using the Benjamini and Hochberg method [168].

### Indicators of influenza A(H3N2) evolution

We considered multiple indicators of influenza evolution based on genetic and phenotypic (serologic) data, separately for HA and NA.

#### HA and NA sequence data

We downloaded all H3 sequences and associated metadata from the Global Initiative on Sharing Avian Influenza Data (GISAID) EpiFlu database [60]. We focused our analysis on complete H3 sequences that were sampled between January 1, 1997, and October 1, 2019. We prioritized viruses with corresponding HI titer measurements provided by the WHO Global Influenza Surveillance and Response System (GISRS) Collaborating Centers and excluded all egg-passaged viruses and sequences with ambiguous year, month, and day annotations. To account for variation in sequence availability across global regions, we subsampled the selected sequences five times to representative sets of 50 viruses per month, with preferential sampling for North America. Each month 25 viruses (when available) were selected from North America, with even sampling across nine other global regions (Africa, Europe, China, South Asia, Japan and Korea, Oceania, South America, Southeast Asia, and West Asia) for the remaining 25 viruses. To ensure proper topology early in the phylogeny, we included reference strains that had been collected no earlier than 5 years prior to January 1, 1997. The resultant sets of H3 sequences included 10,088 to 10,090 sequences spanning December 25, 1995 – October 1, 2019.

As with the H3 analysis, we downloaded all N2 sequences and associated metadata from GISAID and selected complete N2 sequences that were sampled between January 1, 1997, and October 1, 2019. We excluded all sequences with ambiguous year, month, and day annotations, forced the inclusion of reference strains collected no earlier than 5 years prior to January 1, 1997, and compiled five replicate subsampled datasets with preferential sampling for North America (9,007 to 9,009 sequences; June 8, 1995 – October 1, 2019).

#### HA serologic data

Hemagglutination inhibition (HI) measurements from ferret sera were provided by WHO GISRS Collaborating Centers in London, Melbourne, Atlanta, and Tokyo. We converted these raw two-fold dilution measurements to *log*_*2*_ titer drops normalized by the corresponding *log*_*2*_ autologous measurements [35,63].

Although a phenotypic assay exists for NA, NA inhibiting antibody titers are not routinely measured for influenza surveillance. Therefore, we could not include a phenotypic marker of NA evolution in our study.

#### Phylogenetic inference

For each set of H3 and N2 sequences, we aligned sequences with the augur align command [169] and MAFFT v7.407 [170]. We inferred initial phylogenies with IQ-TREE v1.6.10 [171]. To reconstruct time-resolved phylogenies, we applied TreeTime v0.5.6 [172] with the augur refine command [173].

#### Viral fitness metrics

Following Huddleston et al., 2020 [35], we defined the following fitness metrics for each influenza season:

##### Antigenic drift:

We estimated antigenic drift for each H3 strain using either genetic or serologic data. We implemented three sequence-based metrics based on substitutions at putative epitope sites: 129 sites in HA1 [21,64,66,67,174], 7 sites adjacent to the receptor-binding site (RBS) [68], and 34 sites in the HA stalk [70], hereon *HA epitope distance*, *HA RBS distance*, and *HA stalk footprint distance*. To estimate antigenic drift with hemagglutination inhibition (HI) titer data, hereon *HI log*_*2*_ *titer distance*, we applied the phylogenetic tree model from Neher et al., 2016 [63] to the H3 phylogeny and available HI data for its sequences. The tree model estimates the antigenic drift per branch in units of log_2_ titer change.

To estimate N2 antigenic drift, we implemented two sequence-based metrics that count substitutions at putative epitope sites in the NA head: 223 sites [34] or 53 sites [69], hereon *NA epitope distance.*

##### Mutational load:

To estimate mutational load for each H3 and N2 strain, an inverse proxy of viral fitness [61], we implemented metrics that count substitutions at putative non-epitope sites in HA (N = 200) and NA (N = 246), hereon *HA non-epitope distance* and *NA non-epitope distance*. Mutational load metrics produce higher values for strains that are less fit compared to previously circulating strains.

##### Clade growth:

We estimated the seasonal growth of H3 clades and N2 clades with the local branching index (LBI) [62]. To calculate LBI for each H3 and N2 strain, we applied the LBI heuristic algorithm as originally described by Neher et al., 2014 [62] to H3 and N2 phylogenetic trees, respectively. We set the neighborhood parameter, τ, to 0.4 and only considered viruses sampled between the current season t and the previous season t - 1 as contributing to recent clade growth in the current season t. To estimate the diversity of clade growth rates in each season, we binned LBI values by units of 2 into 10 categories ((0–2],(2–4], (4–6], (6–8], (8–10], (10–12],(12–14], (14–16],(16–18], (18–20]) and estimated the Shannon entropy of LBI categories. Here, the Shannon entropy [175] considers both the richness and relative abundance of viral clades with different growth rates and is calculated as follows:

$$H^{\prime }=-\sum_{i}\;p_{i}ln\;p_{i}$$

wherein $p_{i}$ is the proportion of LBI values belonging to the *i*th bin.

#### Antigenic and genetic distance relative to prior seasons

We estimated genetic and antigenic distances between influenza viruses circulating in consecutive seasons by calculating the mean distance between viruses circulating in the current season *t* and viruses circulating during the prior season (t-1 year; one season lag) or two prior seasons ago (t-2 years; two season lag). Seasonal genetic and antigenic distances are greater when currently circulating strains are more antigenically distinct from previously circulating strains. We used Spearman’s correlation coefficients to measure pairwise relationships between scaled H3 and N2 evolutionary indictors. We adjusted P-values for multiple testing using the Benjamini and Hochberg method [168].

### Univariate relationships between viral fitness, (sub)type interference and A(H3N2) epidemic impact

We measured univariate associations between national indicators of A(H3N2) viral fitness and regional A(H3N2) epidemic parameters – peak incidence, epidemic size, effective Rt, epidemic intensity, subtype dominance, excess P&I deaths, onset timing, peak timing, spatiotemporal synchrony, the number of weeks from onset to peak, and seasonal duration. We first measured Spearman correlation coefficients between pairs of scaled fitness indicators and epidemic metrics using 1000 bootstrap replicates of the original dataset (1000 samples with replacement).

Next, we fit regression models with different distribution families (Gaussian or Gamma) and link functions (identity, log, or inverse) to observed data and used Bayesian information criterion (BIC) to select the best fit model, with lower BIC values indicating a better fit to the data. For subtype dominance, epidemic intensity, and age-specific proportions of ILI cases, we fit Beta regression models with logit links. For each epidemic metric, we fit the best-performing regression model to the resampled dataset. To measure the effects of sub(type) interference on A(H3N2) epidemics, the same approach was applied to measure the univariate relationships between A(H1N1) or B epidemic size and A(H3N2) peak incidence, epidemic size, effective Rt, epidemic intensity, and excess mortality. As a sensitivity analysis, we tested univariate relationships between A(H3N2) epidemic metrics and A(H1N1) epidemic size during pre-2009 seasons (seasonal A(H1N1) viruses) and post-2009 seasons (A(H1N1)pdm09 viruses) separately.

All predictors were centered and scaled prior to measuring Spearman’s correlations or fitting regression models.

### Selecting relevant predictors of A(H3N2) epidemic impact

Next, we explored multivariable approaches that would shed light on the potential mechanisms driving annual epidemic impact. Considering that we had many predictors and relatively few observations (22 seasons × 9–10 HHS regions), several covariates were collinear, and our goal was explicative rather than predictive, we settled on methods that tend to select few covariates.

We first used conditional inference random forest models to select relevant predictors of A(H3N2) epidemic size, peak incidence, effective Rt, epidemic intensity, and subtype dominance (party and caret R packages) [176–179]. Candidate predictors included viral fitness indicators: genetic and antigenic distance from previously circulating strains and the Shannon entropy of H3 and N2 LBI values in the current and prior season; proxies for prior natural immunity: A(H3N2) epidemic size in the prior season, influenza A(H1N1) epidemic size and B epidemic size in the current and prior seasons, and the dominant sub(type) in the prior season [12]; and vaccine-related parameters: national adult vaccination coverage in the current and previous season, A(H3N2) vaccine effectiveness in the current and previous season, and H3 and N2 epitope distances between circulating A(H3N2) viruses in the United States and the A(H3N2) vaccine strain in the same season. We did not conduct variable selection analysis for excess A(H3N2) mortality due to data limitations (one national estimate per season). Metrics related to epidemic timing were also excluded from this analysis because we found weak or non-statistically significant associations with most of the candidate evolutionary predictors in univariate analyses.

We created each forest by generating 3,000 regression trees from 10 repeats of a leave-one-season-out (jackknife) cross-validated sample of the data. Due to the small size of our dataset, evaluating the predictive accuracy of random forest models on a quasi-independent test set produced unstable estimates. Consequently, we included all data in the training set and report root mean squared error (RMSE) and R^2^ values from the best tuned model. We used permutation importance (N = 50 permutations) to estimate the relative importance of each predictor in determining model outcomes. Permutation importance is the decrease in prediction accuracy when a single feature (predictor) is randomly permuted, with larger values indicating more important variables. Because our features were collinear, we used conditional permutation importance to compute feature importance scores, rather than the standard marginal procedure [177,178,180,181].

As an alternative method for variable selection, we performed LASSO regression on the same cross-validated dataset and report RMSE and R^2^ values from the best tuned model (glmnet and caret R packages)[179,182]. Unlike random forest models, this approach assumes linear relationships between predictors and the target variable. LASSO models (L1 penalty) are more restrictive than ridge models (L2 penalty) and elastic net models (combination of L1 and L2 penalties) and will arbitrarily select one variable from a set of collinear variables.

To further reduce the set of predictors for each epidemic metric, we performed model selection with linear regression models that considered all combinations of the top 10 ranked predictors from conditional inference random forest models. Candidate models were limited to three independent variables, and models were compared using BIC. We did not include HHS region or season as fixed or random effects in these models because these variables either did not improve model fit (region) or caused convergence issues (season).

All predictors were centered and scaled prior to fitting random forest or regression models.

## Supplementary Material

Supplement 1

Supplement 2

Supplement 3

## Figures and Tables

**Table 1.** **Evolutionary indicators of seasonal viral fitness.** Evolutionary indicators are labeled by the influenza gene for which data are available (hemagglutinin, HA or neuraminidase, NA), the type of data they are based on, and the component of influenza fitness they represent. Table format is adapted from Huddleston et al., 2020 [35].

Evolutionary indicator	Influenza gene	Data type	Fitness category	Citations
Mean HI titer log_2_ distance from the prior season	HA	Hemagglutinin inhibition assays using ferret sera	Antigenic drift	Huddleston et al., 2020; Neher et al., 2016
Mean epitope distance from the prior season	HA and NA	Sequences	Antigenic drift	Bhatt et al., 2011; Bush et al., 1999; Krammer, unpublished; Webster and Laver, 1980; Wiley et al., 1981; Wilson and Cox, 1990; Wolf et al., 2010
Mean receptor binding site distance from the prior season	HA	Sequences	Antigenic drift	Koel et al., 2013
Mutational load (mean non-epitope distance from the prior season)	HA and NA	Sequences	Functional constraint	Łuksza and Lässig, 2014
Mean stalk “footprint” distance from the prior season	HA	Sequences	Negative control	Kirkpatrick et al., 2018
Mean local branching index	HA and NA	Sequences	Clade growth	Huddleston et al., 2020; Łuksza and Lässig, 2014
Shannon entropy of local branching index	HA and NA	Sequences	Diversity of clade growth rates	Huddleston et al., 2020; Neher et al., 2014

**Table 2.** **Seasonal metrics of A(H3N2) epidemic dynamics.** Epidemic metrics are defined and labeled by which outcome category they represent.

Epidemic Outcome	Definition	Outcome category	Citations
Epidemic size	Cumulative weekly incidence	Burden	
Peak incidence	Maximum weekly incidence	Burden	
Maximum time-varying effective reproduction number, Rt	The number of secondary cases arising from a symptomatic index case, assuming conditions remain the same	Transmissibility	Cori et al., 2013; Scott et al., 2021
Epidemic intensity	Inverse Shannon entropy of the weekly incidence distribution (i.e., the spread of incidence across the season)	Sharpness of the epidemic curve	Dalziel et al., 2018
Subtype dominance	The proportion of influenza positive samples typed as A(H3N2)	Viral activity	
Excess pneumonia and influenza mortality attributable to A(H3N2) virus	Mortality burden in excess of a seasonally adjusted baseline	Severity	Hansen et al., 2022; Simonsen and Viboud, 2012
Onset week	Winter changepoint in incidence	Timing	Charu et al., 2017
Peak week	First week of maximum incidence	Timing	
Spatiotemporal synchrony	Variation (s.d.) in regional onset or peak timing	Speed	Viboud et al., 2006
Onset to peak	Number of days between onset week and peak week	Speed	
Seasonal duration	Number of weeks with non-zero incidence	Speed	

**Table 3.** **Predictors of seasonal A(H3N2) epidemic burden, transmissibility, intensity, and subtype dominance.** Variables retained in the best fit model for each epidemic outcome were determined by BIC.

Outcome	Best Minimal Model^1^	R^2^	Adj. R^2^	RMSE
Epidemic Size	H3 epitope distance (t-2) + H1 epidemic size + H3 epidemic size (t-1)	0.74	0.69	9.88
Peak Incidence	H3 epitope distance (t-2) + H1 epidemic size + Dominant IAV Subtype (t-1)	0.69	0.63	2.09
Effective Rt	HI titer distance (t-2) + H1 epidemic size + H3 LBI Diversity (t-1)	0.71	0.65	0.1
Epidemic Intensity	HI titer distance (t-2) + N2 distance to vaccine strain + vaccination coverage (t-1)	0.79	0.75	0.07
Subtype Dominance	H3 epitope distance (t-2) + N2 epitope distance (t-1) + Dominant IAV Subtype (t-1)	0.56	0.48	0.2

1 Candidate models were limited to 3 independent variables and considered all combinations of the top 10 ranked predictors from conditional inference random forest models (Figure 6).

## Data Availability

Sequence data are available from GISAID using accession ids provided in Supplementary file 1. Source code for phylogenetic analyses, inferred HI titers from serological measurements, and evolutionary fitness measurements are available in the GitHub repository https://github.com/blab/perofsky-ili-antigenicity. The five replicate trees for HA and NA can be found at https://nextstrain.org/groups/blab/ under the keyword “perofsky-ili-antigenicity”. Epidemiological data, datasets combining seasonal evolutionary fitness measurements and epidemic metrics, and source code for calculating epidemic metrics and performing statistical analyses are available in the GitHub repository https://github.com/aperofsky/H3N2_Antigenic_Epi. Raw serological measurements are restricted from public distribution by previous data sharing agreements.
