# Supplementary material for: Antigenic drift and subtype interference shape A(H3N2) epidemic dynamics in the United States
Source: medRxiv. 2023 Oct 3:2023.10.02.23296453. Preprint. [Version 1] doi: 10.1101/2023.10.02.23296453 (PMC10593063; doi:10.1101/2023.10.02.23296453)
Supplement: Supplementary file 1 [file NIHPP2023.10.02.23296453v1-supplement-3.pdf]

medRxiv preprint doi: https://doi.org/10.1101/2023.10.02.23296453; this version posted October 3, 2023. The copyright holder for this preprint (which was not certified by peer review) is the author/funder, who has granted medRxiv a license to display the preprint in perpetuity. This article is a US Government work. It is not subject to copyright under 17 USC 105 and is also made available for use under a CC0 license.

Supplementary Methods

Influenza virological surveillance data

Data on weekly influenza type and subtype circulation were obtained from the US CDC's World Health Organization (WHO) Collaborating Center for Surveillance, Epidemiology and Control of Influenza [121]. Approximately 100 public health laboratories and 300 clinical laboratories located throughout the United States report influenza test results to the US CDC, through either the US WHO Collaborating Laboratories Systems or the National Respiratory and Enteric Virus Surveillance System (NREVSS). Clinical laboratories test respiratory specimens for diagnostic purposes whereas public health laboratories primarily test specimens to characterize influenza virus type, subtype, and lineage circulation. Public health laboratories often receive samples that have already tested positive for influenza at a clinical laboratory.

We estimated the weekly number of respiratory samples testing positive for influenza A(H1N1), A(H3N2), or B at the HHS region level. Beginning in the 2015/2016 season, reports from public health and clinical laboratories are presented separately in the CDC's weekly influenza updates. From 2015 week 40 onwards, we used clinical laboratory data to estimate the proportion of respiratory samples testing positive for any influenza type/subtype and the proportion of samples testing positive for influenza A or B. We used public health laboratory data to estimate the proportion of influenza A isolates typed as A(H3N2) or A(H1N1)pdm09 in each week. Untyped influenza A-positive isolates were assigned to either A(H3N2) or A(H1N1) according to their proportions among typed isolates. We combined seasonal and pandemic A(H1N1) as seasonal A(H1N1) influenza and the Victoria and Yamagata lineages of influenza B as influenza B. We defined influenza A subtype dominance in each season based on the proportion of influenza A positive samples typed as A(H1N1) or A(H3N2).

A(H3N2) epidemiological model

Prior to R_t estimation, we computed daily case counts by disaggregating weekly A(H3N2) incidence rates to daily rates (tempdisagg package) [183] and rounding the resultant values to integers. Observed cases were modelled as a function of latent infections in the population, assuming a negative binomial distribution. We assumed an infection ascertainment rate of 0.45 [184], a lognormal-distributed infection-to-symptom-onset time period with mean 1.4 days and standard deviation 1.5 days [185], and a lognormal-distributed onset-to-case-observation time period with mean 2 days and standard deviation 1.5 days [186]. Thus, the time distribution for infection-to-case-observation was

$$\pi \sim \text{lognormal}(1.4, 1.5) + \text{lognormal}(2, 1.5)$$

Instead of using the renewal equation to propagate infections, we treated infections as latent parameters in the model, because the additional variance around infections leads to a posterior distribution that is easier to sample [125]. For the generation time, we assumed a discretized Weibull distribution with mean 3.6 days and standard deviation 1.6 days [187]. To control for temporal autocorrelation, we modelled R_t as a daily random walk. We assigned the intercept a normal prior with mean $\log 2$ and variance 0.2, which gives the initial reproduction number R_0 a prior mean of approximately 2.

Epidemic trajectories for each region and season were fit independently using Stan's Hamiltonian Monte Carlo sampler [188]. For each model, we ran 4 chains, each for 10,000 iterations (including a burn-in period of 2,000 iterations that was discarded), producing a total posterior sample size of 32,000. We verified convergence by confirming that all parameters had sufficiently low R hat values (all R hat < 1.1) and sufficiently large effective sample sizes (>15% of the total sample size).

medRxiv preprint doi: https://doi.org/10.1101/2023.10.02.23296453; this version posted October 3, 2023. The copyright holder for this preprint (which was not certified by peer review) is the author/funder, who has granted medRxiv a license to display the preprint in perpetuity.
This article is a US Government work. It is not subject to copyright under 17 USC 105 and is also made available for use under a CC0 license.

Wavelet analysis

We applied a wavelet approach to quantify the relative timing of influenza A(H3N2), A(H1N1), and B epidemics in each HHS region. Incidence time series were square root transformed and normalized and then padded with zeros to reduce edge effects. Wavelet coherence was used to determine the degree of synchrony between A(H3N2) versus A(H1N1) incidence and A(H3N2) versus B incidence within each region at multi-year time scales. Statistical significance was assessed using 10,000 Monte Carlo simulations. Coherence measures time- and frequency-specific associations between two wavelet transforms, with high coherence indicating that two non-stationary signals (time series) are associated at a particular time and frequency [82].

Following methodology developed for influenza and other viruses [19,82,189-191], we used continuous wavelet transformations (Morlet) to calculate the phase of seasonal A(H3N2), A(H1N1), and B epidemics. We reconstructed weekly time series of phase angles using wavelet reconstruction [19,192] and extracted the major one-year seasonal component (period 0.8 to 1.2 years) of the Morlet decomposition of A(H3N2), A(H1N1), and B time series. To estimate the relative timing of A(H3N2) and A(H1N1) incidence or A(H3N2) and B incidence in each region, phase angle differences were calculated as phase in A(H3N2) minus phase in A(H1N1) (or B), with a positive value indicating that A(H1N1) (or B) lags A(H3N2).

medRxiv preprint doi: https://doi.org/10.1101/2023.10.02.23296453; this version posted October 3, 2023. The copyright holder for this preprint (which was not certified by peer review) is the author/funder, who has granted medRxiv a license to display the preprint in perpetuity.
This article is a US Government work. It is not subject to copyright under 17 USC 105 and is also made available for use under a CC0 license.

Supplementary Figures

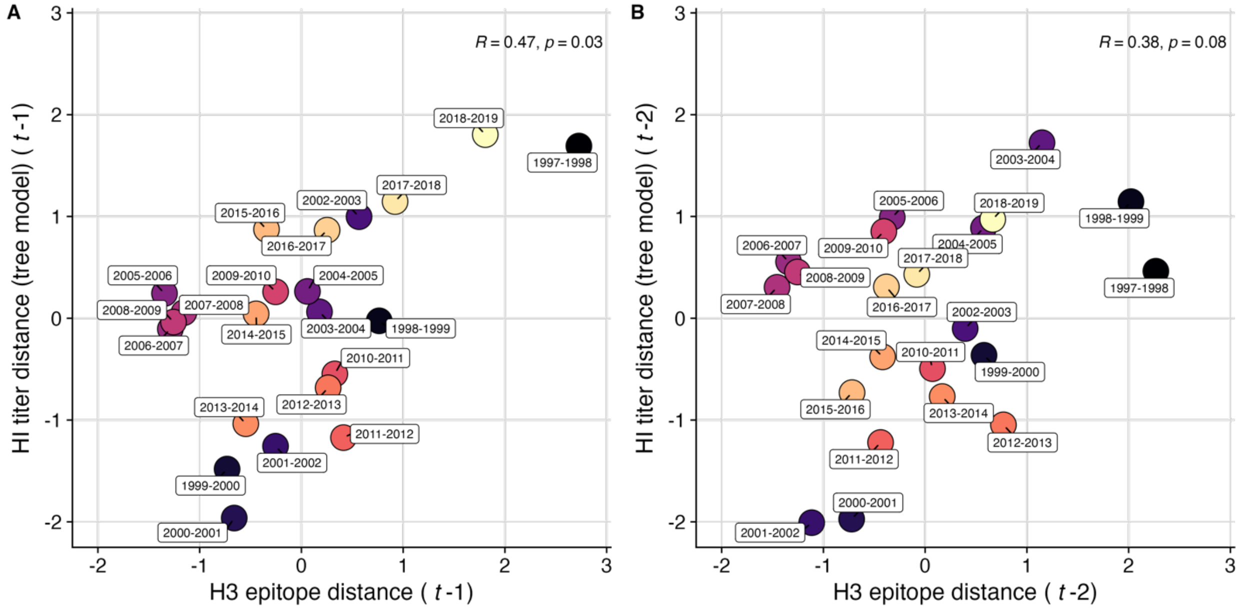

Figure S1. Comparison of seasonal antigenic drift measured by substitutions at hemagglutinin (H3) epitope sites and HI titer measurements, from 1997-1998 to 2018-2019. We used Spearman correlation tests to measure associations between H3 epitope distance and HI titer distance at **A.** one-season lags and **B.** two-season lags. Seasonal antigenic distance is the mean distance between strains circulating in season t and strains circulating in the prior season $t - 1$ year (one season lag) or two seasons ago $t - 2$ years (two season lag). Seasonal distances are scaled because epitope distance and HI titer distance use different units of measurement. Point labels indicate the current influenza season, and point color denotes the relative timing of influenza seasons, with earlier seasons shaded dark purple (e.g., 1997-1998) and later seasons shaded light yellow (e.g., 2018-2019). H3 epitope distance and HI titer (tree model) distance at two-season lags capture expected “jumps” in antigenic drift during key seasons previously associated with major antigenic transitions [32], such as the SY97 cluster seasons (1997-1998, 1998-1999, 1999-2000) and the FU02 cluster season (2003-2004).

medRxiv preprint doi: https://doi.org/10.1101/2023.10.02.23296453; this version posted October 3, 2023. The copyright holder for this preprint (which was not certified by peer review) is the author/funder, who has granted medRxiv a license to display the preprint in perpetuity.
This article is a US Government work. It is not subject to copyright under 17 USC 105 and is also made available for use under a CC0 license.

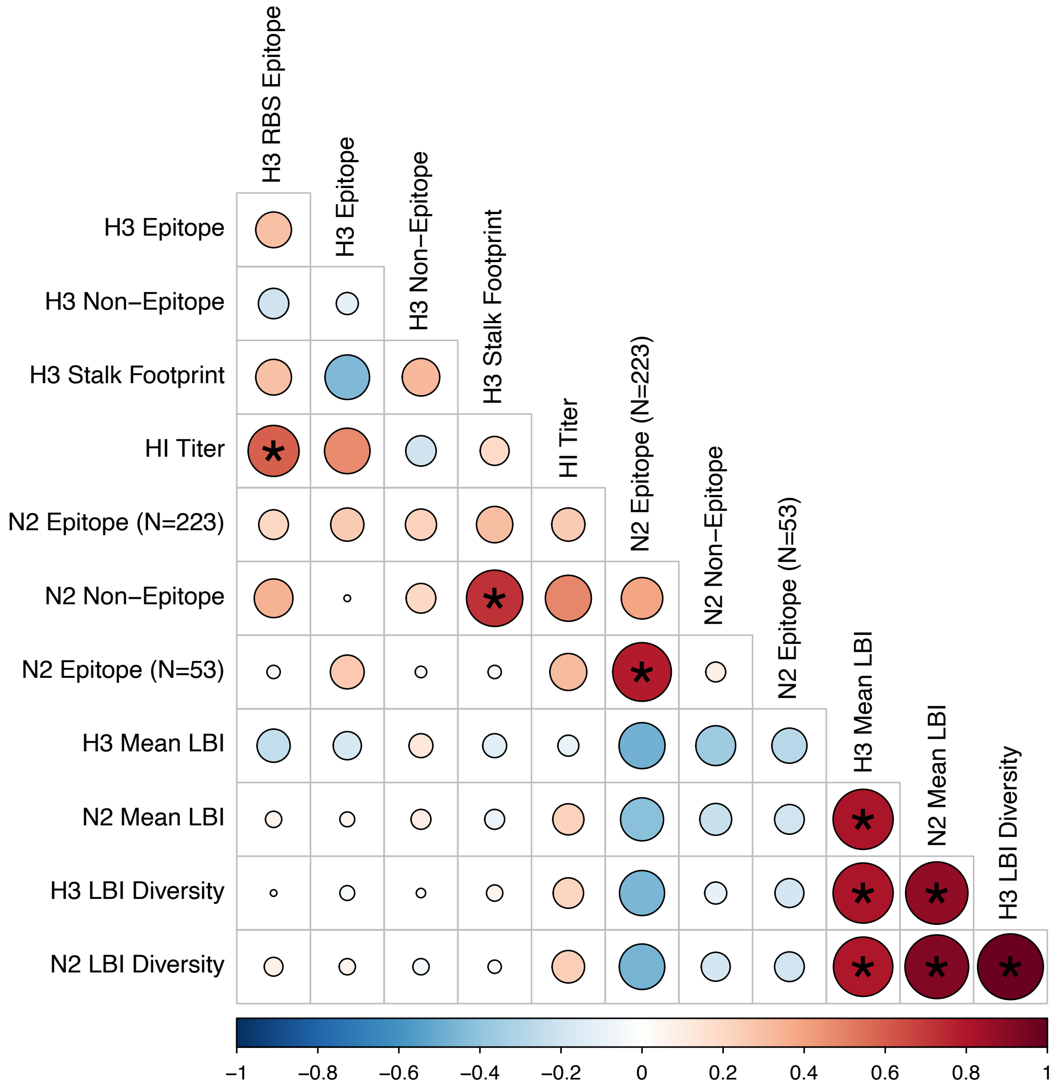

Figure S2. Pairwise correlations between H3 and N2 evolutionary indicators (one season lags). We measured Spearman's correlations between seasonal measures of H3 and N2 evolution, including H3 RBS distance, H3 epitope distance, H3 non-epitope distance, H3 stalk footprint distance, HI titer distance, N2 epitope distance based on 223 or 53 epitope sites, N2 non-epitope distance, mean clade growth of H3 and N2 (local branching index, LBI), and the Shannon entropy of H3 and N2 LBI values. Seasonal distances were estimated as the mean distance between strains circulating in the current season *t* and those circulating in the prior season ($t-1$). The Benjamini and Hochberg method was used to adjust P-values for multiple testing. The color of each circle indicates the strength and direction of the association, from dark red (strong positive correlation) to dark blue (strong negative correlation). Stars within circles indicate statistical significance (adjusted $P < 0.05$).

medRxiv preprint doi: https://doi.org/10.1101/2023.10.02.23296453; this version posted October 3, 2023. The copyright holder for this preprint (which was not certified by peer review) is the author/funder, who has granted medRxiv a license to display the preprint in perpetuity.
This article is a US Government work. It is not subject to copyright under 17 USC 105 and is also made available for use under a CC0 license.

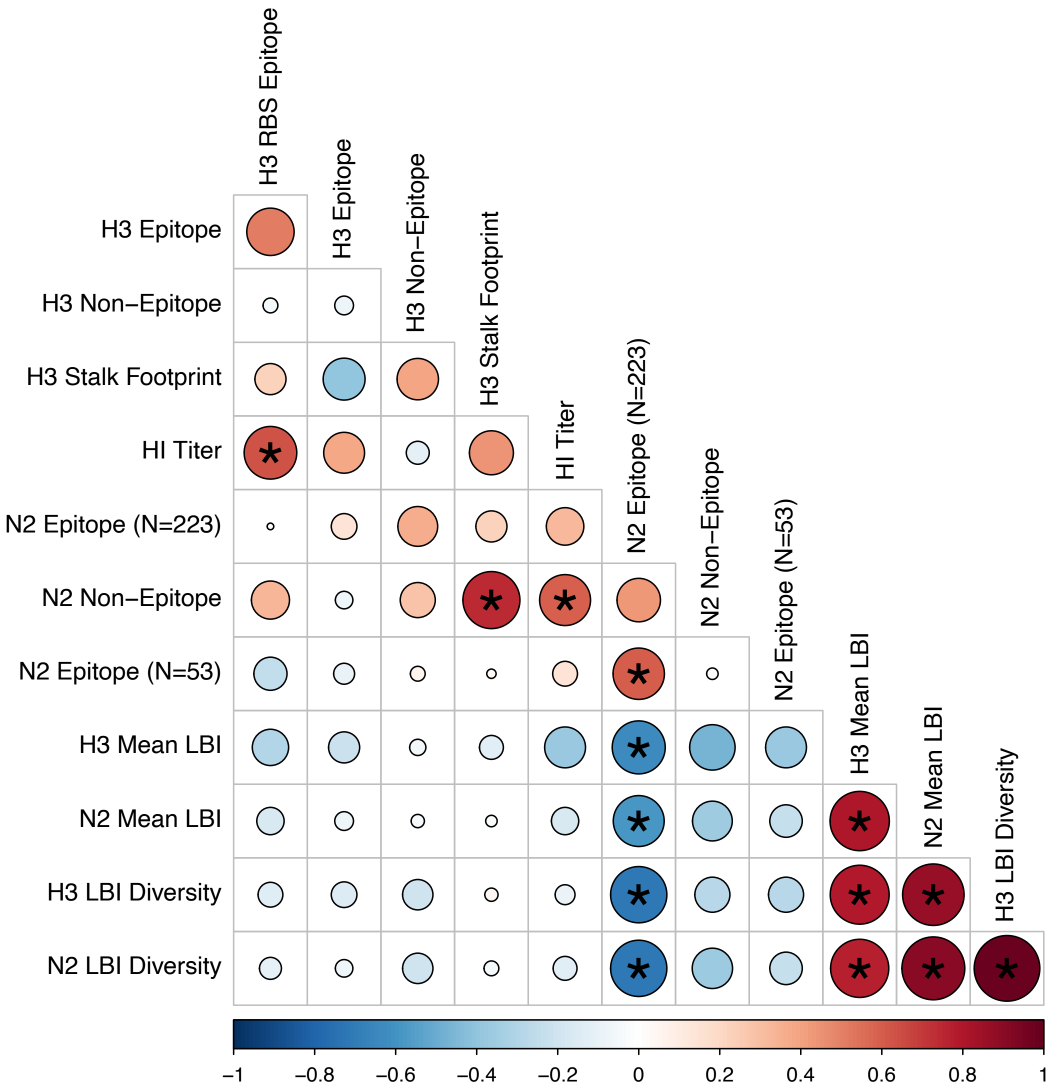

Figure S3. Pairwise correlations between H3 and N2 evolutionary indicators (two season lags). We measured Spearman's correlations between seasonal measures of H3 and N2 evolution, including H3 RBS distance, H3 epitope distance, H3 non-epitope distance, H3 stalk footprint distance, HI titer distance (tree model), N2 epitope distance based on 223 or 53 epitope sites, N2 non-epitope distance, mean clade growth of H3 and N2 (local branching index, LBI), and the Shannon entropy of H3 and N2 LBI values. Seasonal distances were estimated as the mean distance between strains circulating in the current season t and those circulating in the prior season ($t-1$). The Benjamini and Hochberg method was used to adjust P-values for multiple testing. The color of each circle indicates the strength and direction of the association, from dark red (strong positive correlation) to dark blue (strong negative correlation). Stars within circles indicate statistical significance (adjusted $P < 0.05$).

medRxiv preprint doi: https://doi.org/10.1101/2023.10.02.23296453; this version posted October 3, 2023. The copyright holder for this preprint (which was not certified by peer review) is the author/funder, who has granted medRxiv a license to display the preprint in perpetuity.
This article is a US Government work. It is not subject to copyright under 17 USC 105 and is also made available for use under a CC0 license.

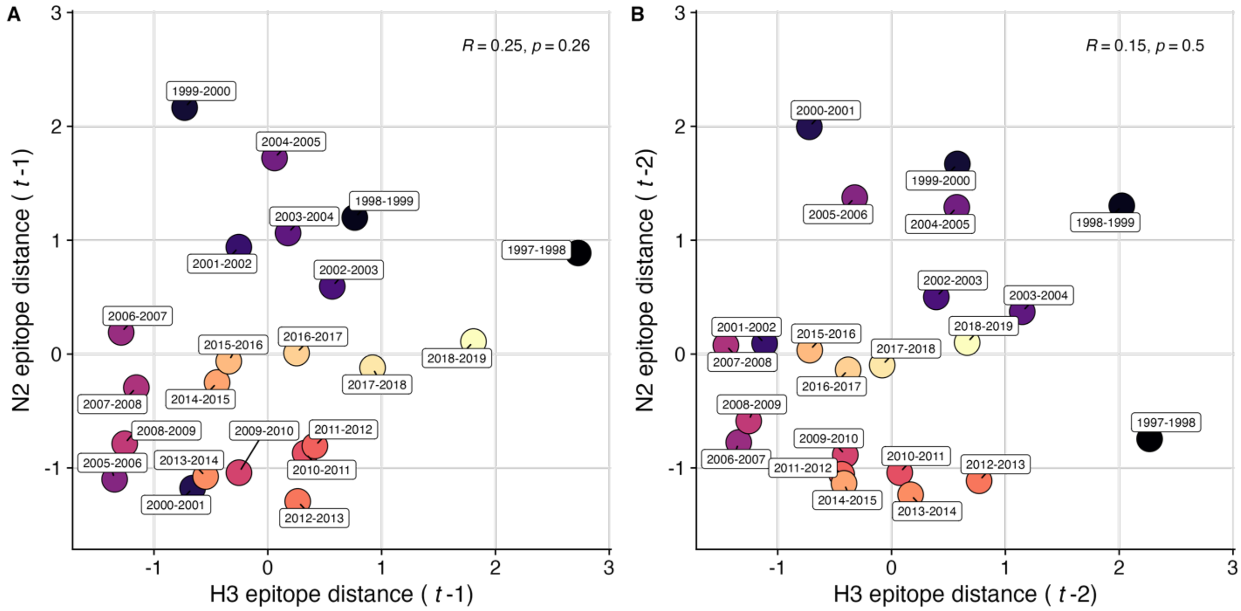

Figure S4. Comparison of seasonal antigenic drift measured by substitutions at hemagglutinin (H3) and neuraminidase (N2) epitope sites, from 1997-1998 to 2018-2019. We used Spearman correlation tests to measure associations between H3 epitope distance and N2 epitope distance at **A.** one-season lags and **B.** two-season lags. Seasonal epitope distance is the mean distance between strains circulating in season t and strains circulating in the prior season $t-1$ (one season lag) or two seasons ago $t-2$ (two season lag). Point labels indicate the current influenza season, and point color denotes the relative timing of influenza seasons, with earlier seasons shaded dark purple (e.g., 1997-1998) and later seasons shaded light yellow (e.g., 2018-2019). N2 epitope distance at one-season lags captures expected “jumps” in antigenic drift during key seasons previously associated with major antigenic transitions [32], such as the SY97 cluster seasons (1997-1998, 1998-1999, 1999-2000) the FU02 cluster season (2003-2004), and the CA04 cluster season (2004-2005).

medRxiv preprint doi: https://doi.org/10.1101/2023.10.02.23296453; this version posted October 3, 2023. The copyright holder for this preprint (which was not certified by peer review) is the author/funder, who has granted medRxiv a license to display the preprint in perpetuity.
This article is a US Government work. It is not subject to copyright under 17 USC 105 and is also made available for use under a CC0 license.

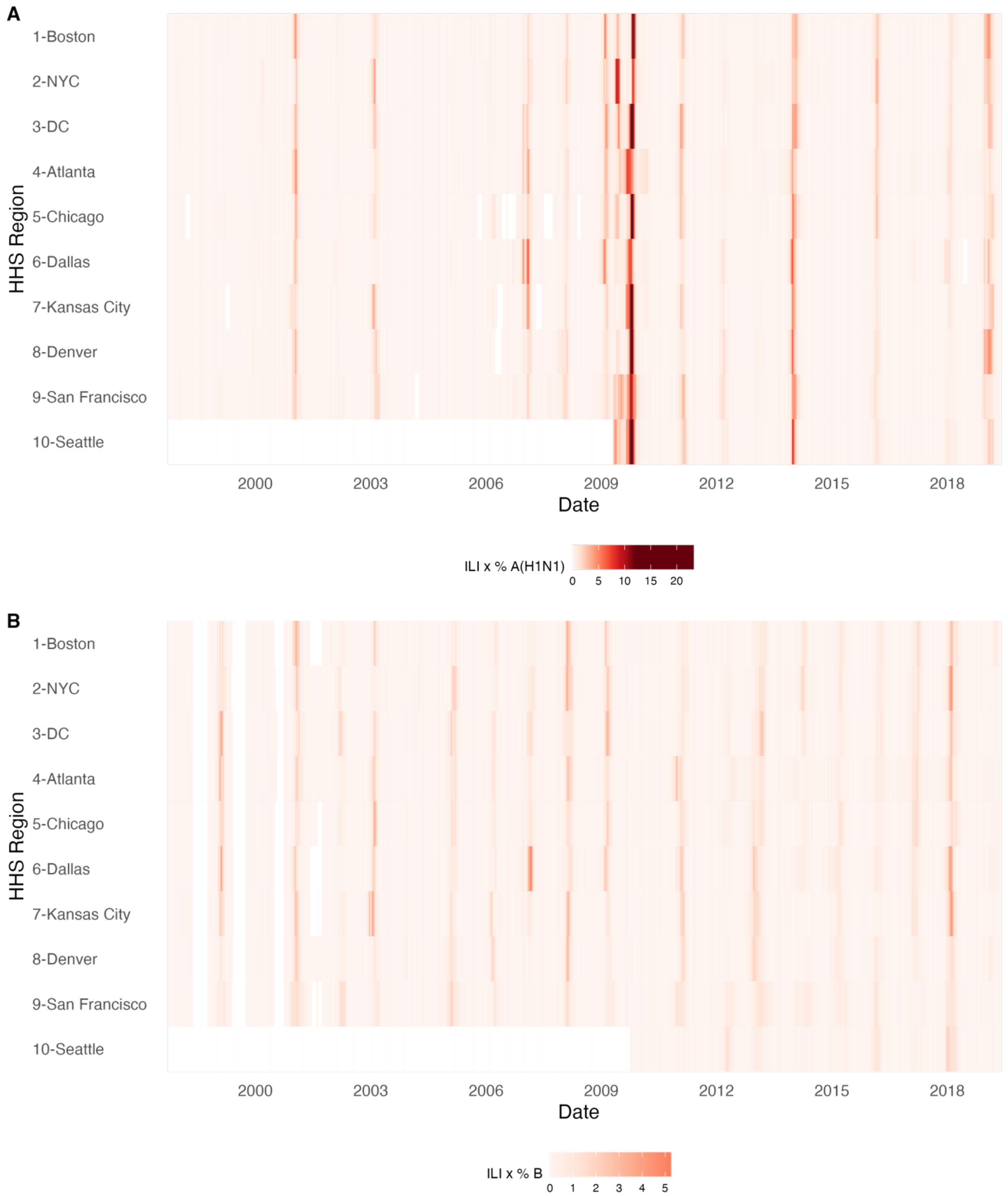

Figure S5. Intensity of weekly incidence of A. influenza A(H1N1) and B. influenza B in ten HHS regions, 1997 - 2019. Seasonal and pandemic A(H1N1) were combined as A(H1N1), and the Victoria and Yamagata lineages of influenza B were combined as influenza B. White tiles indicate weeks when either influenza-like-illness cases or virological data were not reported. Data for Region 10 were not available in seasons prior to 2009.

medRxiv preprint doi: https://doi.org/10.1101/2023.10.02.23296453; this version posted October 3, 2023. The copyright holder for this preprint (which was not certified by peer review) is the author/funder, who has granted medRxiv a license to display the preprint in perpetuity.
This article is a US Government work. It is not subject to copyright under 17 USC 105 and is also made available for use under a CC0 license.

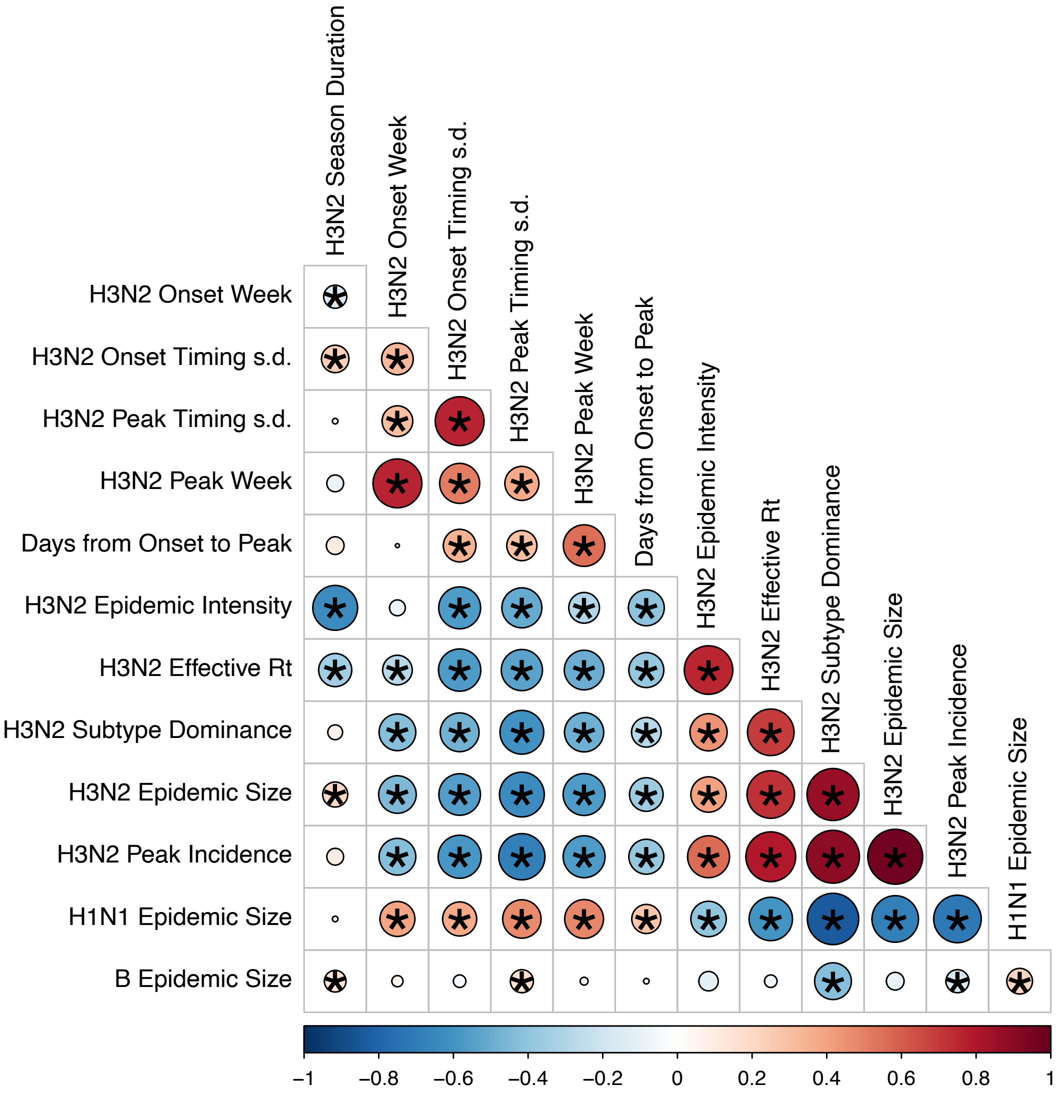

Figure S6. Pairwise correlations between seasonal A(H3N2), A(H1N1), and B epidemic metrics. We measured Spearman's correlations among indicators of A(H3N2) epidemic timing, including onset week, peak week, regional variation (s.d.) in onset and peak timing, and the number of days from onset to peak, indicators of A(H3N2) epidemic magnitude, including epidemic intensity (i.e., the "sharpness" of the epidemic curve), transmissibility (maximum effective reproduction number, Rt), subtype dominance patterns, epidemic size, and peak incidence. We also considered relationships between the circulation of other types/subtypes and A(H3N2) epidemic burden and timing. The Benjamini and Hochberg method was used to adjust P-values for multiple testing. The color of each circle indicates the strength and direction of the association, from dark red (strong positive correlation) to dark blue (strong negative correlation). Stars within circles indicate statistical significance (adjusted $P < 0.05$).

medRxiv preprint doi: https://doi.org/10.1101/2023.10.02.23296453; this version posted October 3, 2023. The copyright holder for this preprint (which was not certified by peer review) is the author/funder, who has granted medRxiv a license to display the preprint in perpetuity.
This article is a US Government work. It is not subject to copyright under 17 USC 105 and is also made available for use under a CC0 license.

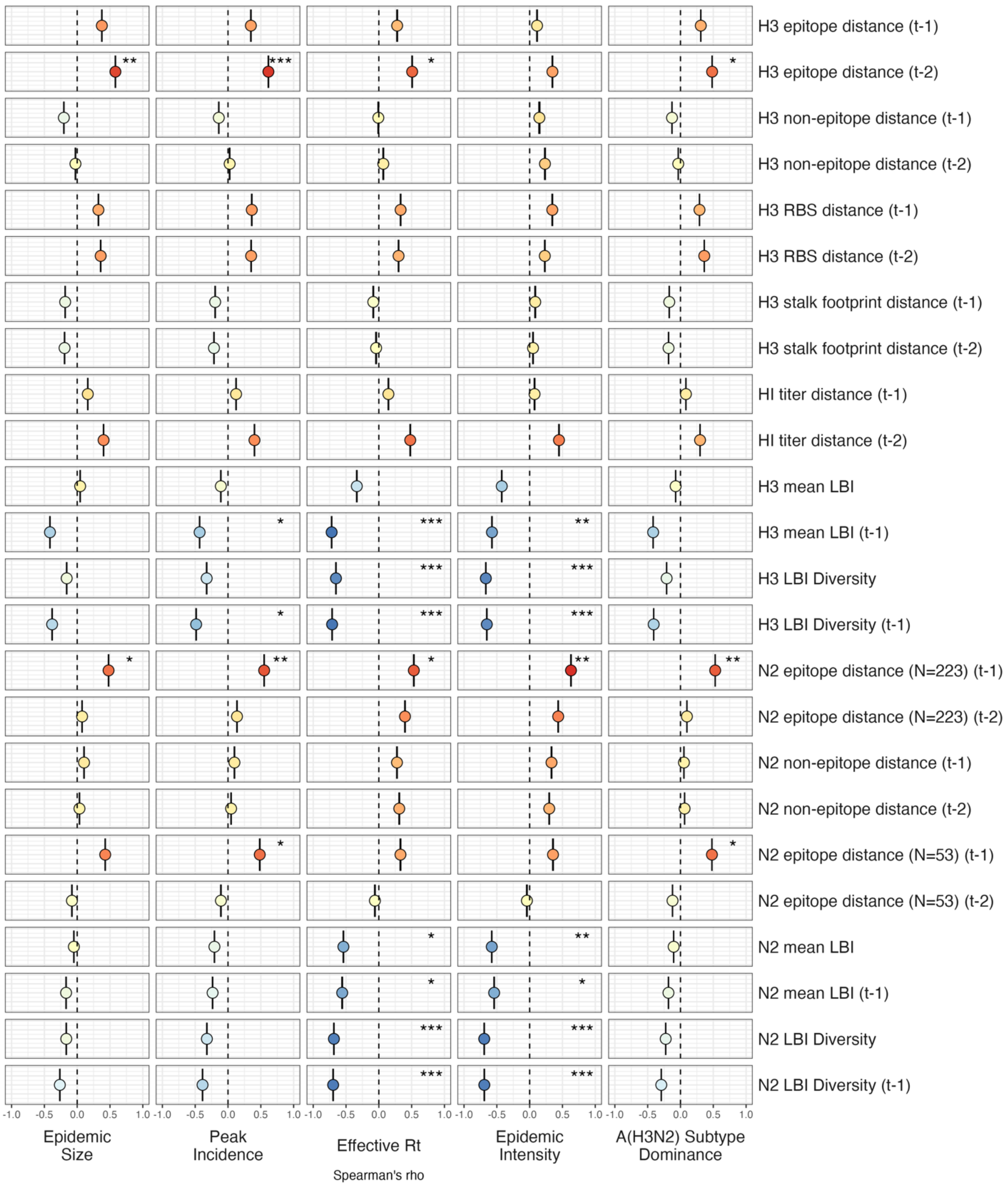

Figure S7. Univariate correlations between A(H3N2) viral fitness and epidemic impact. Mean Spearman correlation coefficients, 95% confidence intervals of correlation coefficients, and corresponding p-values of bootstrapped (N = 1000) viral fitness indicators (rows) and epidemic metrics (columns). Point color indicates the strength and direction of the association, from dark red (strong positive correlation) to dark blue (strong negative correlation), and stars indicate statistical significance (* $P < 0.05$, ** $P < 0.01$, *** $P < 0.001$). Abbreviations: HI = hemagglutination inhibition, RBS: receptor binding site, t - 1 = one-season lag, t - 2 = two-season lag, LBI = local branching index.

medRxiv preprint doi: https://doi.org/10.1101/2023.10.02.23296453; this version posted October 3, 2023. The copyright holder for this preprint (which was not certified by peer review) is the author/funder, who has granted medRxiv a license to display the preprint in perpetuity.
This article is a US Government work. It is not subject to copyright under 17 USC 105 and is also made available for use under a CC0 license.

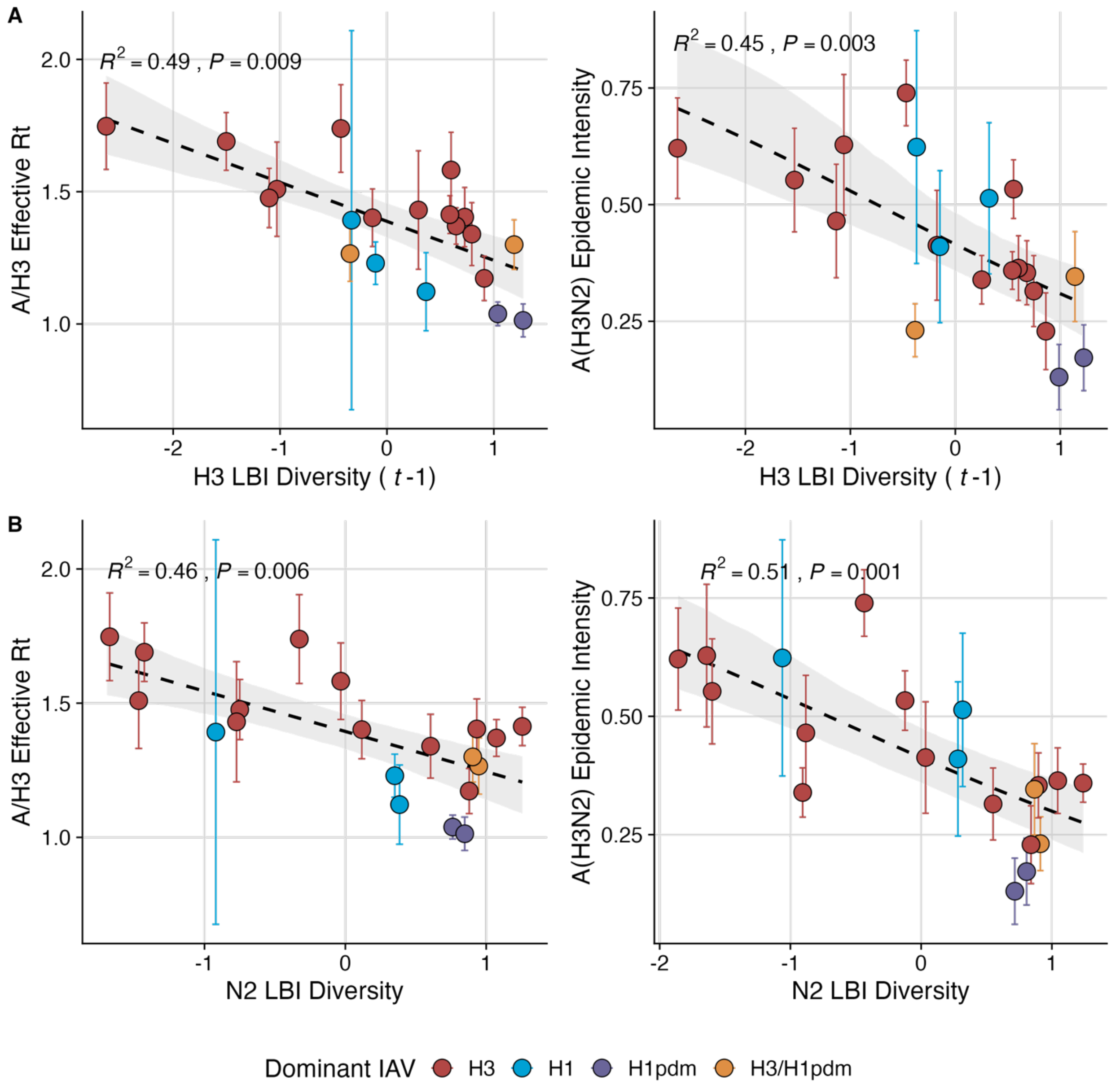

Figure S8. Low diversity in the growth rates of circulating A(H3N2) clades is associated with more intense epidemics and higher transmissibility. A(H3N2) effective Rt and epidemic intensity negatively correlate with the diversity of LBI values among circulating A(H3N2) lineages in the current or prior season, measured by the Shannon entropy of **A.** H3 local branching index (LBI) values in the prior season ($t-1$), and **B.** the Shannon entropy of N2 LBI values in the current season t. LBI values are scaled to aid in direct comparisons of H3 and N2 LBI diversity. Point color indicates the dominant influenza A subtype based on CDC influenza season summary reports (red: A(H3N2), blue: A(H1N1), purple: A(H1N1)pdm09, orange: A(H3N2)/A(H1N1)pdm09 co-dominant), and vertical bands are 95% confidence intervals of regional estimates. Mean A(H3N2) epidemic metric values were fit as a function of seasonal LBI diversity using Gaussian GLMs (effective Rt: inverse link) or Beta GLMs (epidemic intensity: logit link) with 1000 bootstrap resamples.

medRxiv preprint doi: https://doi.org/10.1101/2023.10.02.23296453; this version posted October 3, 2023. The copyright holder for this preprint (which was not certified by peer review) is the author/funder, who has granted medRxiv a license to display the preprint in perpetuity.
This article is a US Government work. It is not subject to copyright under 17 USC 105 and is also made available for use under a CC0 license.

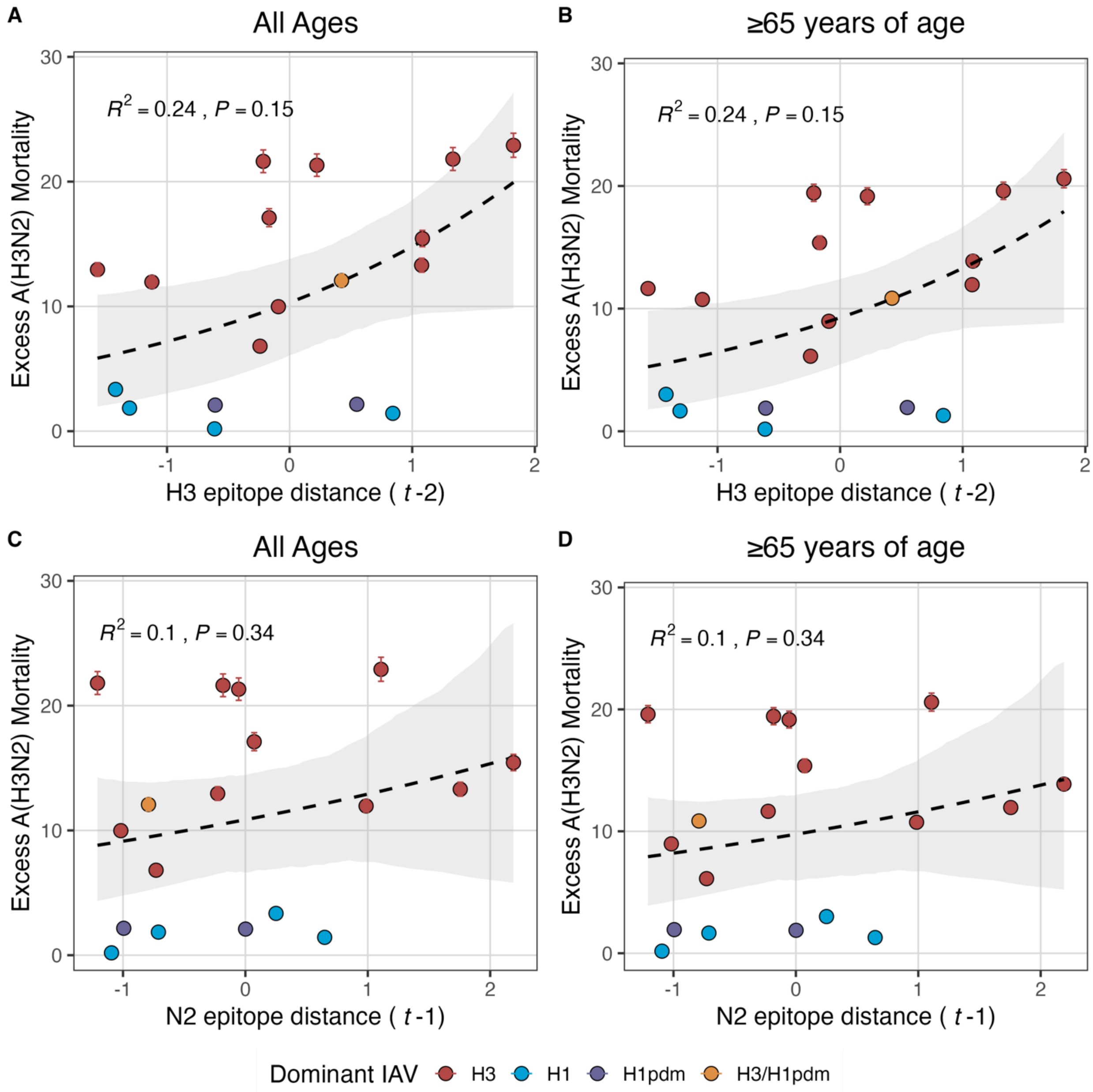

Figure S9. Excess influenza A(H3N2) mortality increases with H3 and N2 antigenic drift, but correlations are not statistically significant. The number of excess influenza deaths attributable to A(H3N2) (per 100,000 people) were estimated from a seasonal regression model fit to weekly pneumonia and influenza-coded deaths [127]. Seasonal epitope distance is the mean distance between strains circulating in season t and those circulating in the prior season ($t - 1$) or two seasons ago ($t - 2$). Distances are scaled to aid in direct comparison of evolutionary indicators. Point color indicates the dominant influenza A subtype based on CDC influenza season summary reports (red: A(H3N2), blue: A(H1N1), purple: A(H1N1)pdm09, orange: A(H3N2)/A(H1N1)pdm09 co-dominant), and vertical bars are 95% confidence intervals of excess mortality estimates. National excess mortality estimates were fit as a function of seasonal H3 or N2 epitope distance using Gaussian GLMs (log link) with 1000 bootstrap resamples.

medRxiv preprint doi: https://doi.org/10.1101/2023.10.02.23296453; this version posted October 3, 2023. The copyright holder for this preprint (which was not certified by peer review) is the author/funder, who has granted medRxiv a license to display the preprint in perpetuity. This article is a US Government work. It is not subject to copyright under 17 USC 105 and is also made available for use under a CC0 license.

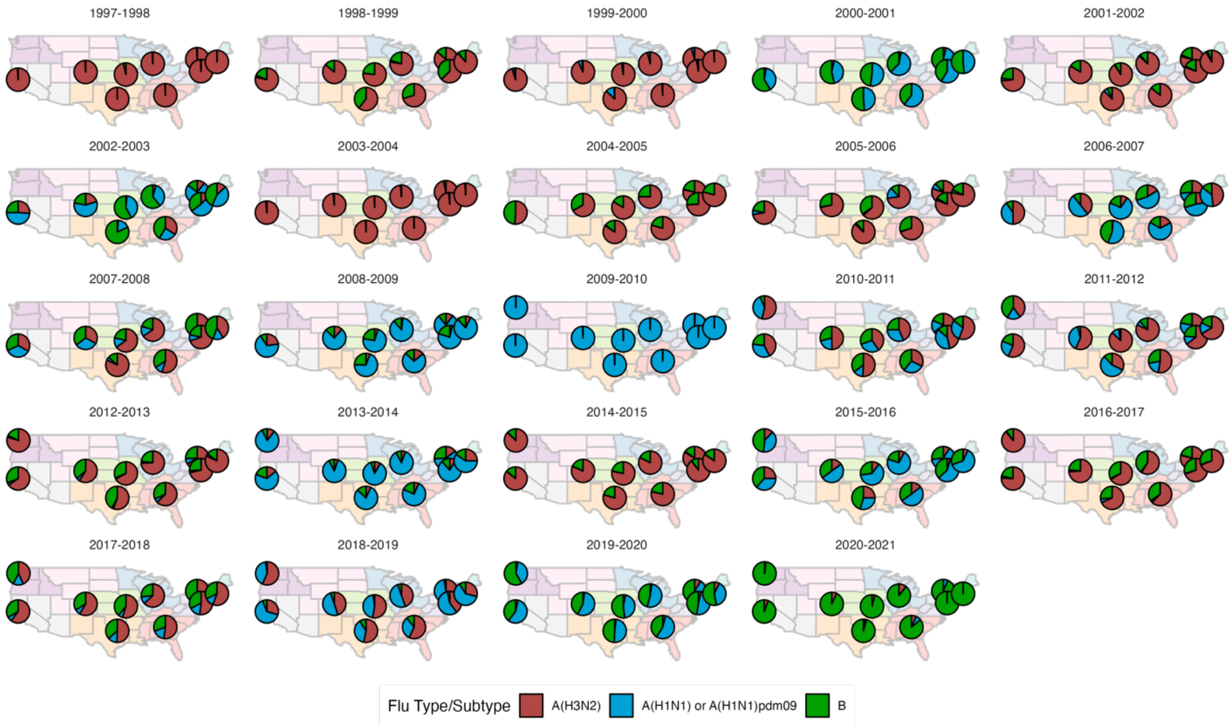

Figure S10. Regional patterns of influenza type and subtype incidence from seasons 1997-1998 to 2018-2019. Pie charts represent the proportion of influenza positive samples that were typed as A(H3N2), A(H1N1) or A(H1N1)pdm09, and B in each HHS region. Data for Region 10 (purple) were not available in seasons prior to the 2009 A(H1N1) pandemic.

medRxiv preprint doi: https://doi.org/10.1101/2023.10.02.23296453; this version posted October 3, 2023. The copyright holder for this preprint (which was not certified by peer review) is the author/funder, who has granted medRxiv a license to display the preprint in perpetuity.
This article is a US Government work. It is not subject to copyright under 17 USC 105 and is also made available for use under a CC0 license.

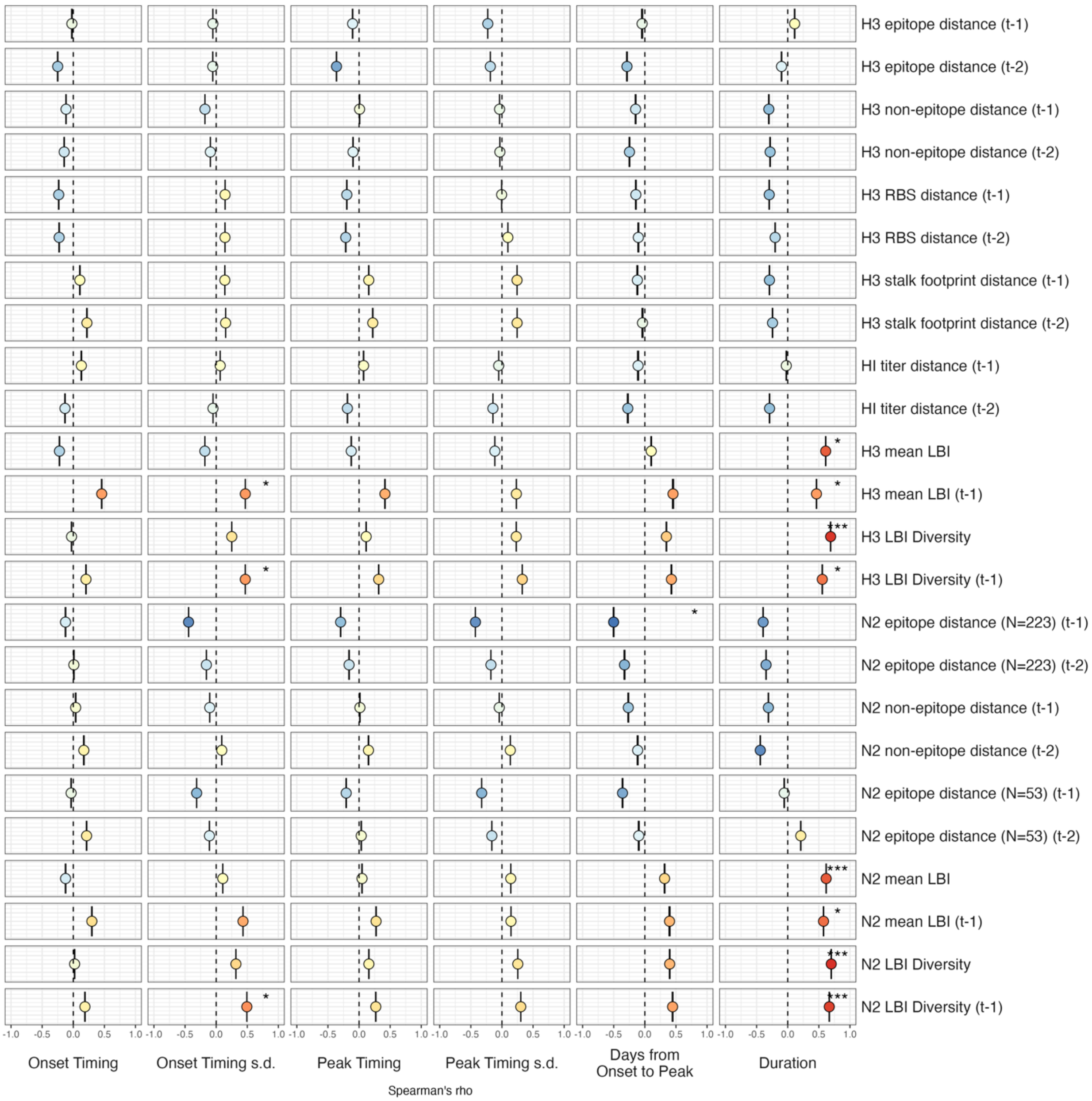

Figure S11. Univariate correlations between A(H3N2) viral fitness and epidemic timing. Mean Spearman correlation coefficients, 95% confidence intervals of correlation coefficients, and corresponding p-values of bootstrapped (N = 1000) viral fitness indicators (columns) and epidemic timing metrics (rows). Epidemic timing metrics are the week of epidemic onset, regional variation (s.d.) in onset timing, the week of epidemic peak, regional variation (s.d.) in peak timing, the number of days between epidemic onset and peak, and seasonal duration. Color indicates the strength and direction of the association, from dark red (strong positive correlation) to dark blue (strong negative correlation), and stars indicate statistical significance (* P < 0.05, ** P < 0.01, *** P < 0.001). Abbreviations: HI = hemagglutination inhibition, RBS: receptor binding site, t - 1 = one-season lag, t - 2 = two-season lag, LBI = local branching index.

medRxiv preprint doi: https://doi.org/10.1101/2023.10.02.23296453; this version posted October 3, 2023. The copyright holder for this preprint (which was not certified by peer review) is the author/funder, who has granted medRxiv a license to display the preprint in perpetuity. This article is a US Government work. It is not subject to copyright under 17 USC 105 and is also made available for use under a CC0 license.

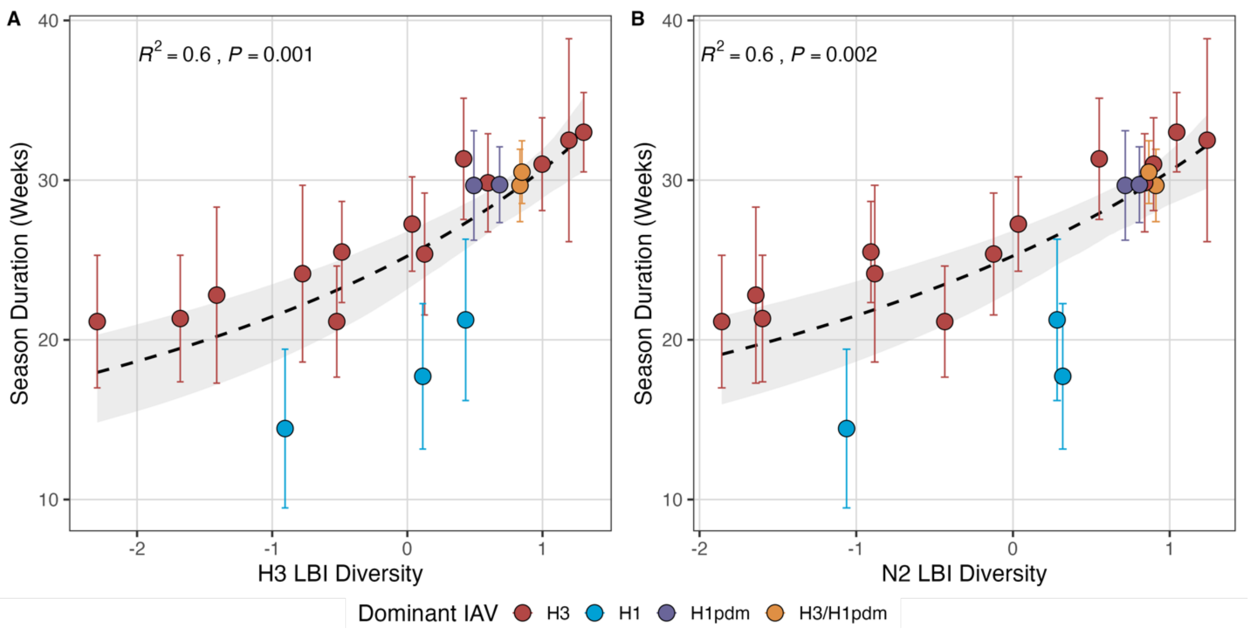

Figure S12. Seasonal duration increases with diversity in clade growth rates of circulating H3 and N2 lineages, measured as the Shannon entropy of local branching index (LBI) values. A. H3 LBI diversity and **B.** N2 LBI diversity during the current season positively correlate with seasonal duration. LBI values are scaled to aid in direct comparisons of H3 and N2 LBI diversity. Point color indicates the dominant influenza A subtype based on CDC influenza season summary reports (red: A(H3N2), blue: A(H1N1), purple: A(H1N1)pdm09, orange: A(H3N2)/A(H1N1)pdm09 co-dominant). Mean values of regional season duration were fit as a function of H3 LBI diversity or N2 LBI diversity using Gaussian GLMs (inverse link) with 1000 bootstrap resamples.

medRxiv preprint doi: https://doi.org/10.1101/2023.10.02.23296453; this version posted October 3, 2023. The copyright holder for this preprint (which was not certified by peer review) is the author/funder, who has granted medRxiv a license to display the preprint in perpetuity.
This article is a US Government work. It is not subject to copyright under 17 USC 105 and is also made available for use under a CC0 license.

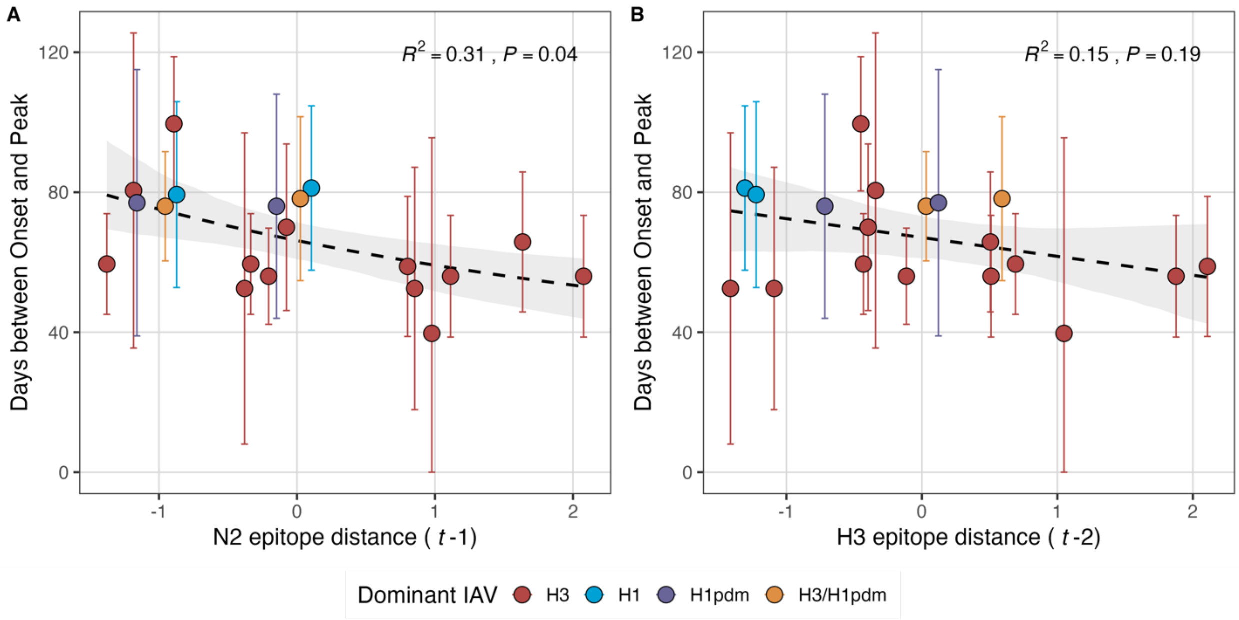

Figure S13. Epidemic speed increases with N2 antigenic drift. N2 epitope distance correlates with fewer days from epidemic onset to peak (**A**), while the relationship between H3 epitope distance and epidemic speed is less apparent (**B**). Seasonal epitope distance is the mean distance between strains circulating in season *t* and those circulating in the prior season ($t - 1$) or two seasons ago ($t - 2$). Distances are scaled to aid in direct comparison of evolutionary indicators. Point color indicates the dominant influenza A subtype based on CDC influenza season summary reports (red: A(H3N2), blue: A(H1N1), purple: A(H1N1)pdm09, orange: A(H3N2)/A(H1N1)pdm09 co-dominant). Mean values of regional days from onset to peak were fit as a function of H3 or N2 epitope distance using Gamma GLMs (inverse link) with 1000 bootstrap resamples.

medRxiv preprint doi: https://doi.org/10.1101/2023.10.02.23296453; this version posted October 3, 2023. The copyright holder for this preprint (which was not certified by peer review) is the author/funder, who has granted medRxiv a license to display the preprint in perpetuity.
This article is a US Government work. It is not subject to copyright under 17 USC 105 and is also made available for use under a CC0 license.

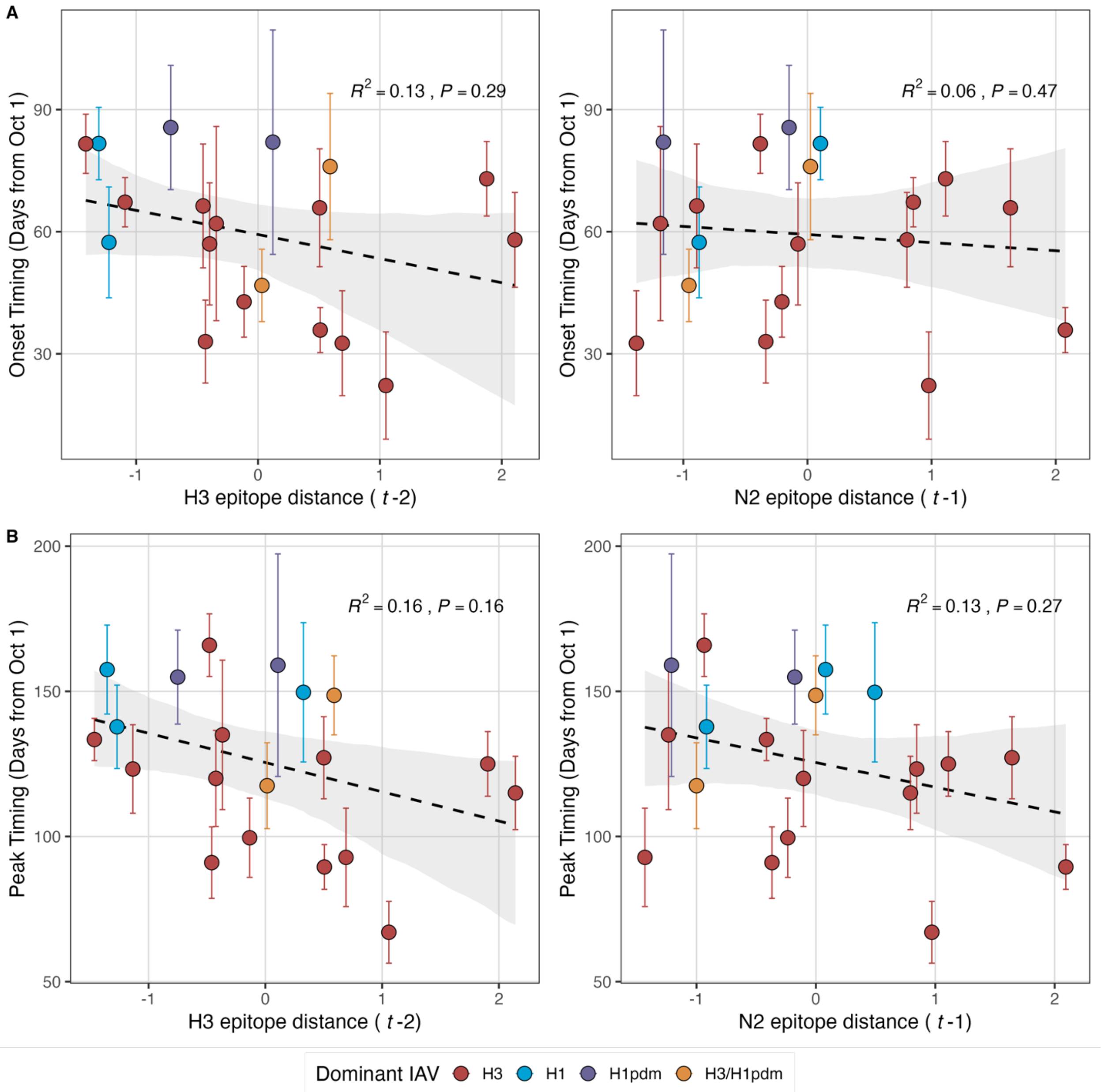

Figure S14. The timing of epidemic onsets and peaks are weakly correlated with H3 and N2 antigenic change. A. Epidemic onsets are earlier in seasons with increased H3 epitope distance ($t-2$), but the correlation is not statistically significant. **B.** Epidemic peaks are earlier in seasons with increased H3 epitope distance ($t-2$) or increased N2 epitope distance ($t-1$), but correlations are not statistically significant. Seasonal epitope distance is the mean distance between strains circulating in season t and those circulating in the prior season ($t-1$) or two seasons ago ($t-2$). Distances are scaled to aid in direct comparison of evolutionary indicators. Point color indicates the dominant influenza A subtype based on CDC influenza season summary reports (red: A(H3N2), blue: A(H1N1), purple: A(H1N1)pdm09, orange: A(H3N2)/A(H1N1)pdm09 co-dominant). Mean values of regional epidemic onsets and peaks were fit as a function of H3 or N2 epitope distance using LMs with 1000 bootstrap resamples.

medRxiv preprint doi: https://doi.org/10.1101/2023.10.02.23296453; this version posted October 3, 2023. The copyright holder for this preprint (which was not certified by peer review) is the author/funder, who has granted medRxiv a license to display the preprint in perpetuity.
This article is a US Government work. It is not subject to copyright under 17 USC 105 and is also made available for use under a CC0 license.

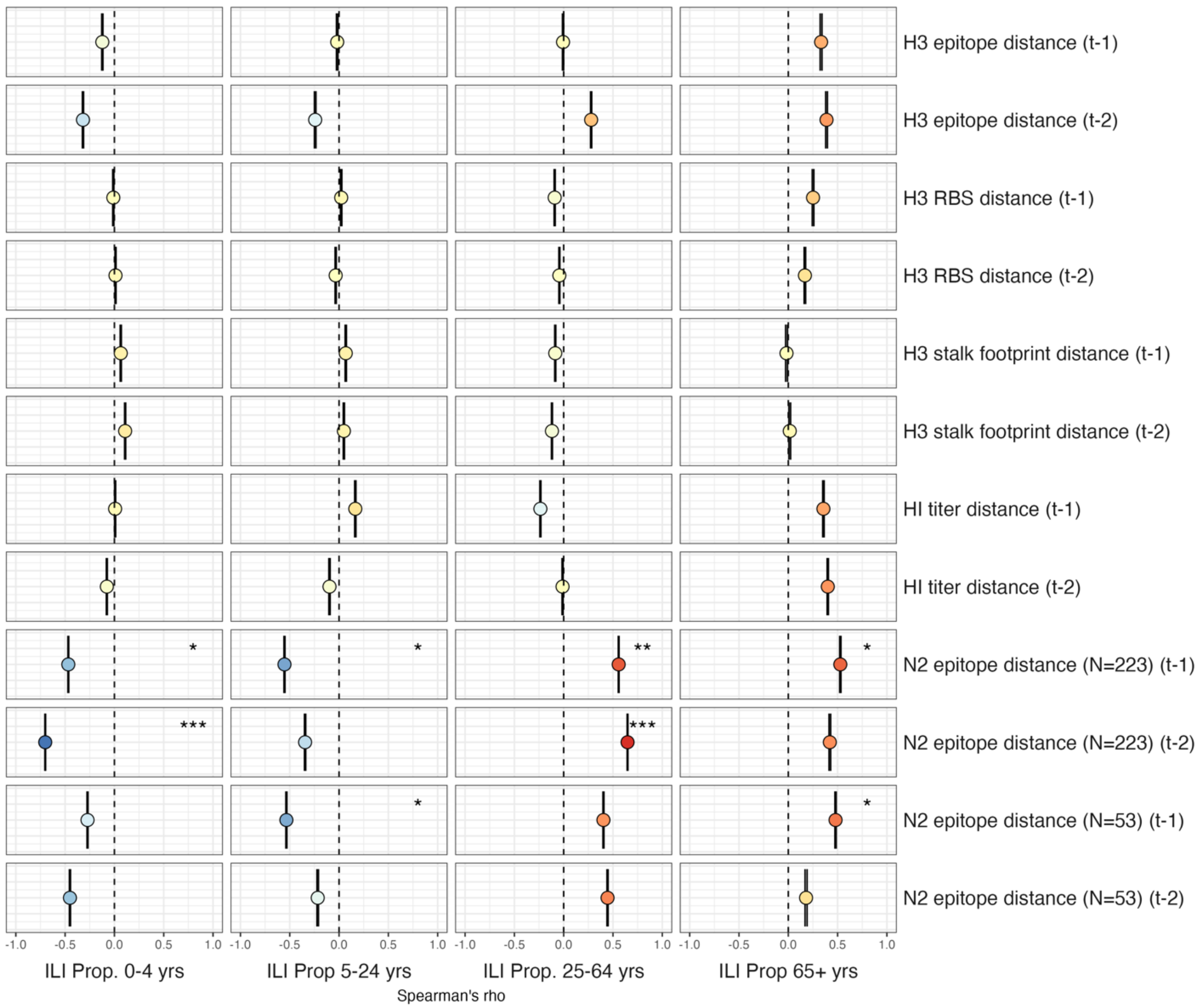

Figure S15. Univariate correlations between A(H3N2) antigenic change and the age distribution of outpatient influenza-like illness (ILI) cases. Mean Spearman correlation coefficients, 95% confidence intervals of correlation coefficients, and corresponding p-values of bootstrapped (N = 1000) evolutionary indicators (rows) and the proportion of ILI cases in individuals aged < 5 years, 5-24 years, 25-64 years, and ≥ 65 years (columns). Color indicates the strength and direction of the association, from dark red (strong positive correlation) to dark blue (strong negative correlation), and stars indicate statistical significance (* $P < 0.05$, ** $P < 0.01$, *** $P < 0.001$). Abbreviations: HI = hemagglutination inhibition, RBS: receptor binding site, t - 1 = one-season lag, t - 2 = two-season lag.

medRxiv preprint doi: https://doi.org/10.1101/2023.10.02.23296453; this version posted October 3, 2023. The copyright holder for this preprint (which was not certified by peer review) is the author/funder, who has granted medRxiv a license to display the preprint in perpetuity.
This article is a US Government work. It is not subject to copyright under 17 USC 105 and is also made available for use under a CC0 license.

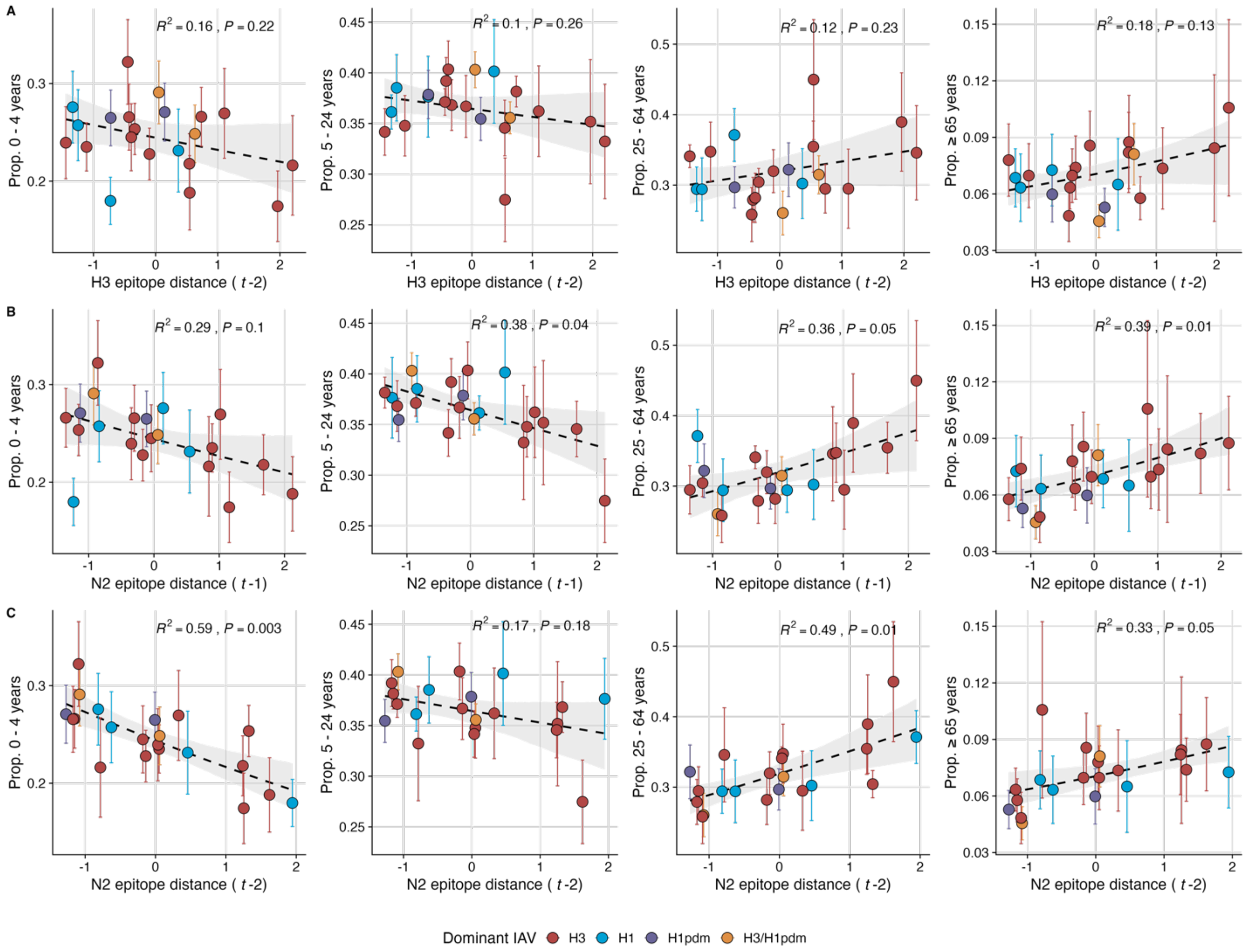

Figure S16. N2 epitope distance correlates with the age distribution of outpatient influenza-like illness (ILI) cases. Seasonal epitope distance is the mean distance between strains circulating in season t and those circulating in the prior season ($t-1$) or two seasons ago ($t-2$). Distances are scaled to aid in direct comparison of evolutionary indicators. Point color indicates the dominant influenza A subtype based on CDC influenza season summary reports (red: A(H3N2), blue: A(H1N1), purple: A(H1N1)pdm09, orange: A(H3N2)/A(H1N1)pdm09 co-dominant), and vertical bars are 95% confidence intervals of regional age distribution estimates. The fraction of cases in each age group were fit as a function of seasonal H3 or N2 epitope distance using Beta GLMs (logit link) with 1000 bootstrap resamples.

medRxiv preprint doi: https://doi.org/10.1101/2023.10.02.23296453; this version posted October 3, 2023. The copyright holder for this preprint (which was not certified by peer review) is the author/funder, who has granted medRxiv a license to display the preprint in perpetuity. This article is a US Government work. It is not subject to copyright under 17 USC 105 and is also made available for use under a CC0 license.

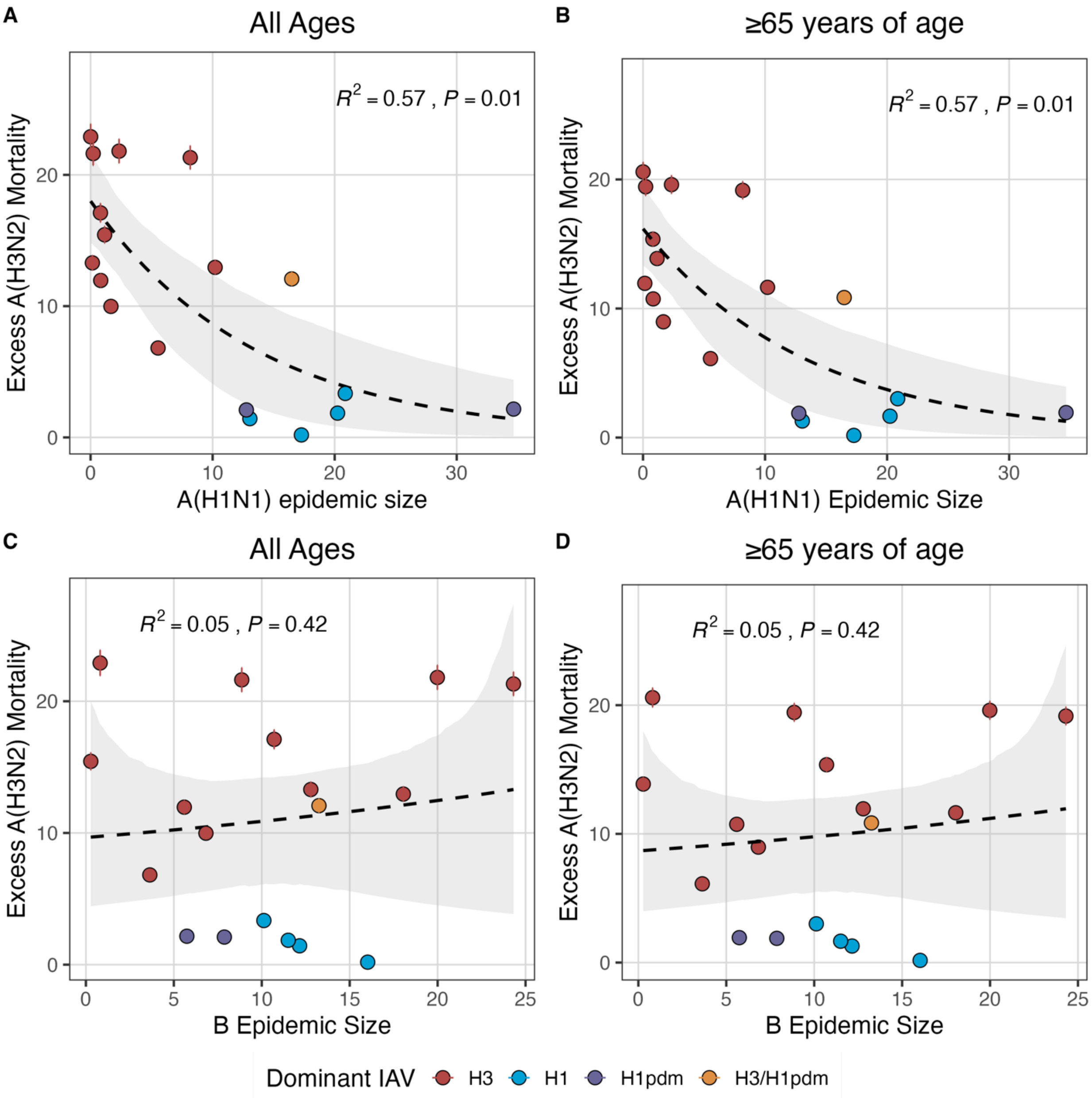

Figure S17. National excess influenza A(H3N2) mortality decreases with A(H1N1) epidemic size but not B epidemic size. Excess influenza deaths attributable to A(H3N2) (per 100,000 people) were estimated from a seasonal regression model fit to weekly pneumonia and influenza-coded deaths . Point color indicates the dominant influenza A subtype based on CDC influenza season summary reports (red: A(H3N2), blue: A(H1N1), purple: A(H1N1)pdm09, orange: A(H3N2)/A(H1N1)pdm09 co-dominant), and vertical bands are 95% confidence intervals of model estimates. National excess mortality estimates were fit as a function of seasonal A(H1N1) or B epidemic size using Gaussian GLMs (log link) with 1000 bootstrap resamples.

medRxiv preprint doi: https://doi.org/10.1101/2023.10.02.23296453; this version posted October 3, 2023. The copyright holder for this preprint (which was not certified by peer review) is the author/funder, who has granted medRxiv a license to display the preprint in perpetuity.
This article is a US Government work. It is not subject to copyright under 17 USC 105 and is also made available for use under a CC0 license.

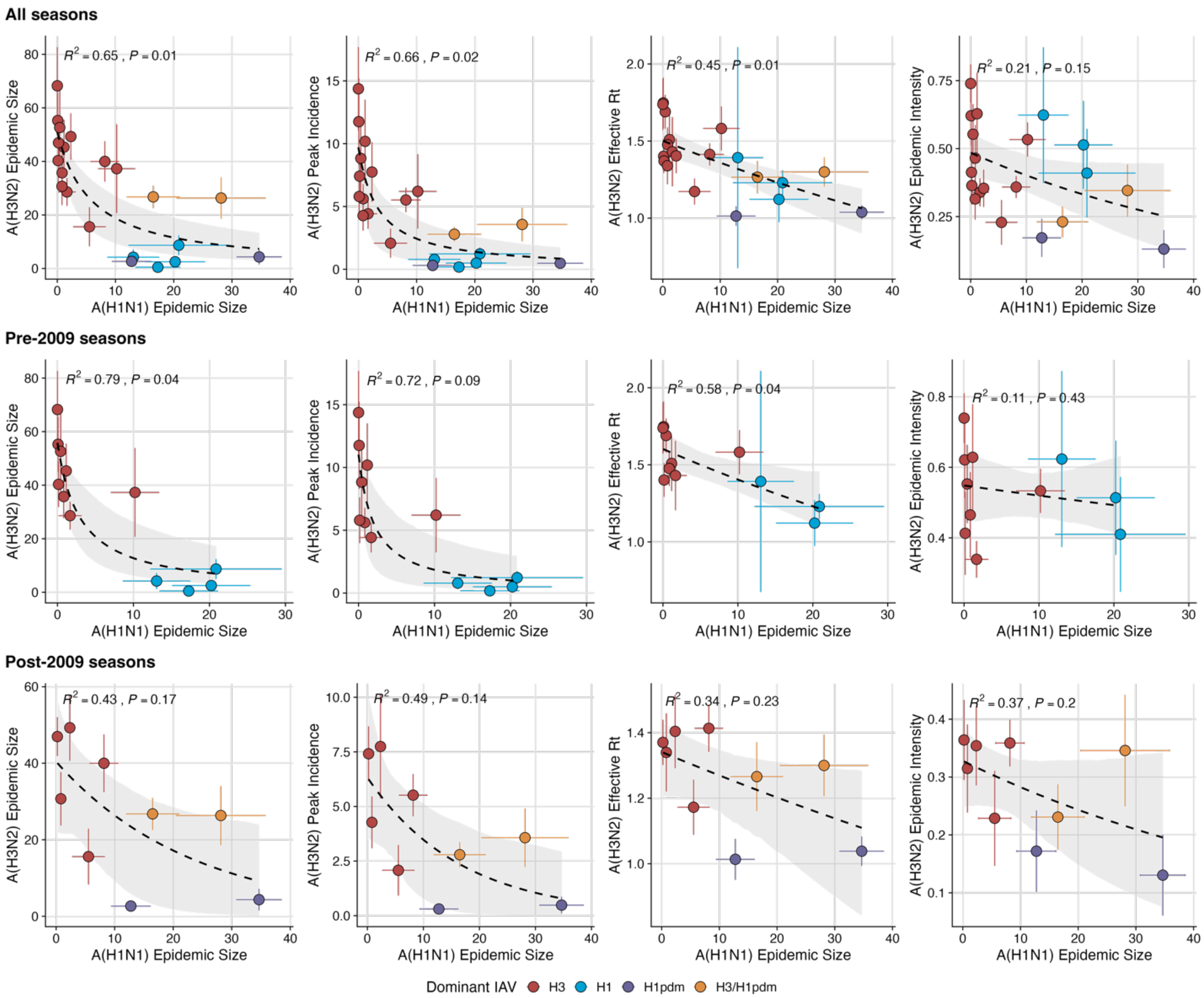

Figure S18. The effect of influenza A(H1N1) epidemic size on A(H3N2) epidemic burden during the entire study period (1997-2019) (top), pre-2009 seasons (middle), and post-2009 seasons (bottom). Influenza A(H1N1) epidemic size inversely correlates with A(H3N2) epidemic size, peak incidence, transmissibility (maximum effective reproduction number, Rt), and epidemic intensity. Point color indicates the dominant influenza A virus (IAV) subtype based on CDC influenza season summary reports (red: A(H3N2), blue: A(H1N1), purple: A(H1N1)pdm09, orange: A(H3N2)/A(H1N1)pdm09 co-dominant), and vertical and horizontal bands are 95% confidence intervals of regional estimates. Seasonal mean A(H3N2) epidemic metrics were fit as a function of mean A(H1N1) epidemic size using Gaussian GLMs (epidemic size, peak incidence: inverse link; effective Rt: log link) or Beta GLMs (epidemic intensity: logit link) with 1000 bootstrap resamples.

medRxiv preprint doi: https://doi.org/10.1101/2023.10.02.23296453; this version posted October 3, 2023. The copyright holder for this preprint
(which was not certified by peer review) is the author/funder, who has granted medRxiv a license to display the preprint in perpetuity.
This article is a US Government work. It is not subject to copyright under 17 USC 105 and is also made available
for use under a CC0 license.

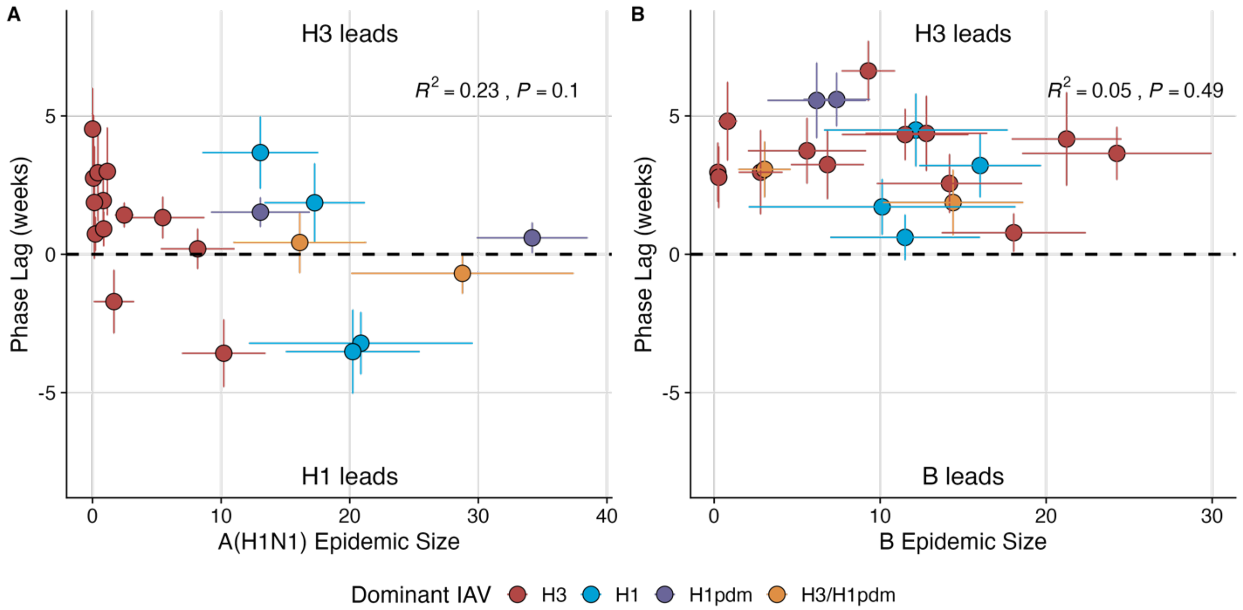

Figure S19. Wavelet analysis of influenza A and B epidemic timing. A. A(H3N2) incidence precedes A(H1N1) incidence in most seasons. Although A(H1N1) incidence sometimes leads or is in phase with A(H3N2) incidence (negative or zero phase lag), the direction of seasonal phase lags is not clearly associated with A(H1N1) epidemic size. **B.** A(H3N2) incidence leads B incidence (positive phase lag) during each season, irrespective of B epidemic size. Point color indicates the dominant influenza A subtype based on CDC influenza season summary reports (red: A(H3N2), blue: A(H1N1), purple: A(H1N1)pdm09, orange: A(H3N2)/A(H1N1)pdm09 co-dominant), and vertical bars are 95% confidence intervals of regional estimates. To estimate the relative timing of influenza subtype incidences, phase angle differences were calculated as phase in A(H3N2) minus phase in A(H1N1) (or B), with a positive value indicating that A(H1N1) (or B) incidence lags A(H3N2) incidence. To calculate seasonal phase lags, we averaged pairwise phase angle differences from epidemic week 40 to epidemic week 20. Seasonal phase lags were fit as a function of seasonal A(H1N1) or B epidemic size using LMs with 1000 bootstrap resamples.

medRxiv preprint doi: https://doi.org/10.1101/2023.10.02.23296453; this version posted October 3, 2023. The copyright holder for this preprint (which was not certified by peer review) is the author/funder, who has granted medRxiv a license to display the preprint in perpetuity.
This article is a US Government work. It is not subject to copyright under 17 USC 105 and is also made available for use under a CC0 license.

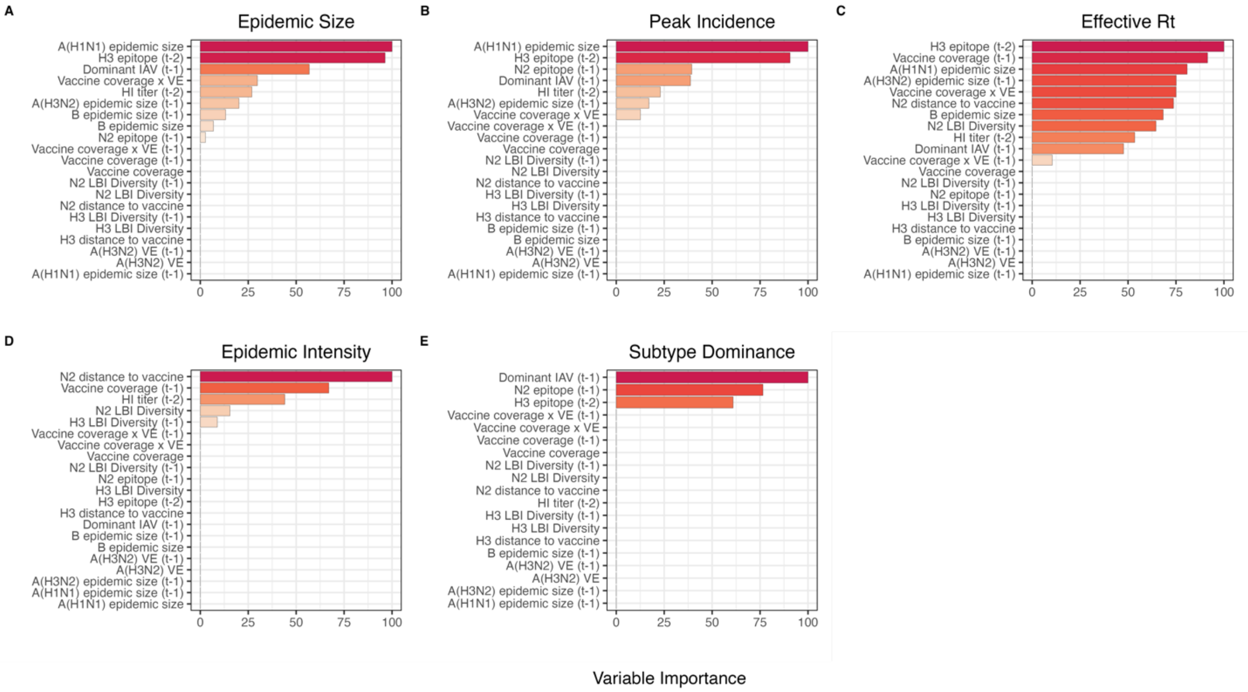

Figure S20. Variable importance rankings from LASSO models predicting A(H3N2) epidemic dynamics. Ranking of variables in predicting seasonal A(H3N2) **A.** epidemic size, **B.** peak incidence, **C.** transmissibility (effective reproduction number, Rt), **D.** epidemic intensity (inverse Shannon entropy), and **E.** subtype dominance. Models were tuned using a repeated leave-one-season-out cross-validated sample of the data. Variables are ranked by their coefficient estimates, with differences in prediction accuracy scaled by the total (null model) error. Abbreviations: HI titer = hemagglutination inhibition $\log_2$ titer distance, t - 1 = one-season lag, t - 2 = two-season lag, LBI = local branching index, peak = peak incidence, distance to vaccine = epitope distance between currently circulating strains and the recommended vaccine strain, VE = vaccine effectiveness.

medRxiv preprint doi: https://doi.org/10.1101/2023.10.02.23296453; this version posted October 3, 2023. The copyright holder for this preprint (which was not certified by peer review) is the author/funder, who has granted medRxiv a license to display the preprint in perpetuity. This article is a US Government work. It is not subject to copyright under 17 USC 105 and is also made available for use under a CC0 license.

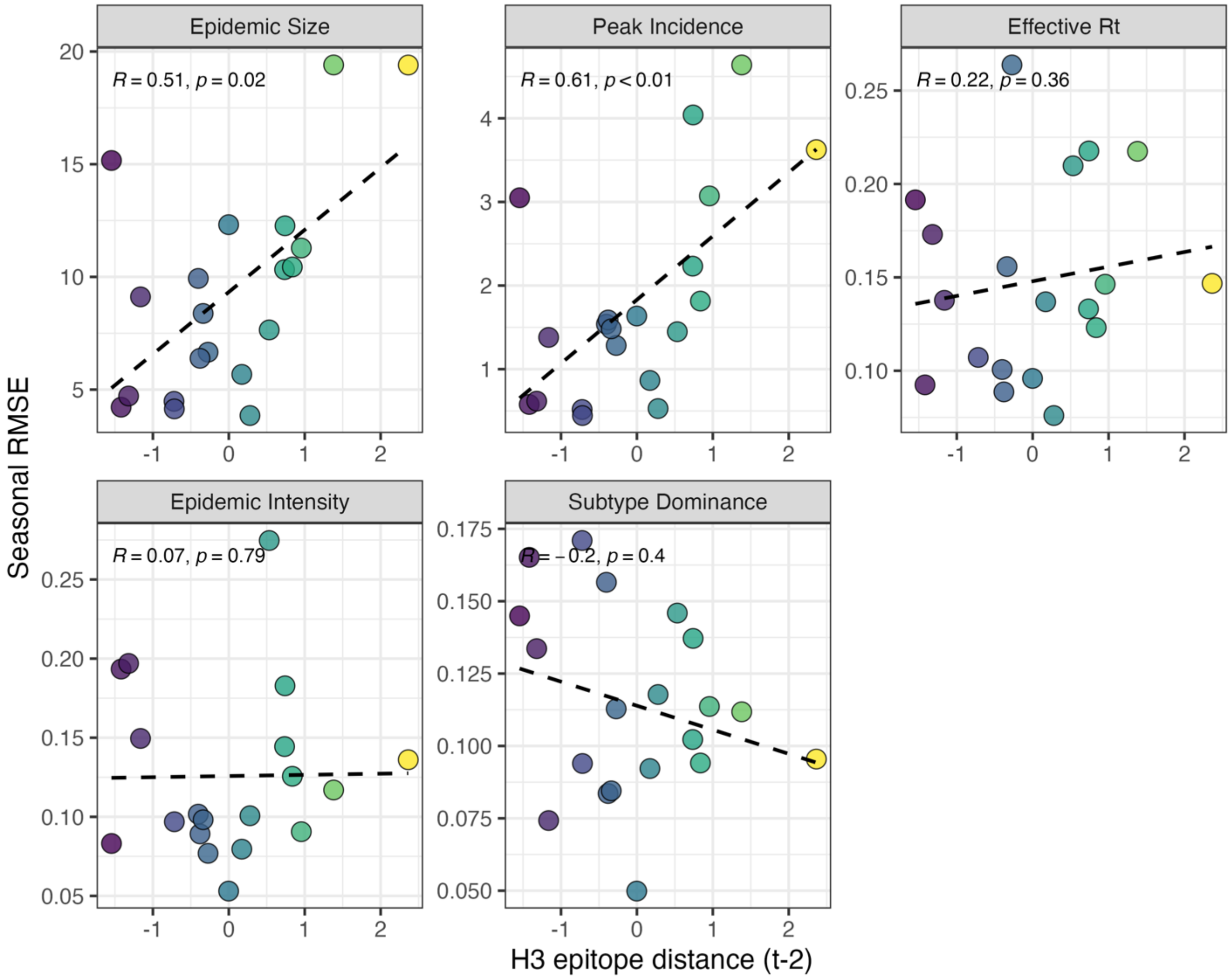

Figures S21. Relationships between the predictive accuracy of random forest models and H3 epitope distance. Root mean squared errors between observed and model-predicted values were averaged across regions for each season, and results are facetted according to epidemic metric. Point color corresponds to the degree of H3 epitope distance in viruses circulating in season *t* relative to those circulating two seasons ago (*t* – *2*), with bright yellow points indicating seasons with greater antigenic novelty. Spearman correlation coefficients and associated P-values are provided in the top left section of each facet.

medRxiv preprint doi: https://doi.org/10.1101/2023.10.02.23296453; this version posted October 3, 2023. The copyright holder for this preprint (which was not certified by peer review) is the author/funder, who has granted medRxiv a license to display the preprint in perpetuity.
This article is a US Government work. It is not subject to copyright under 17 USC 105 and is also made available for use under a CC0 license.

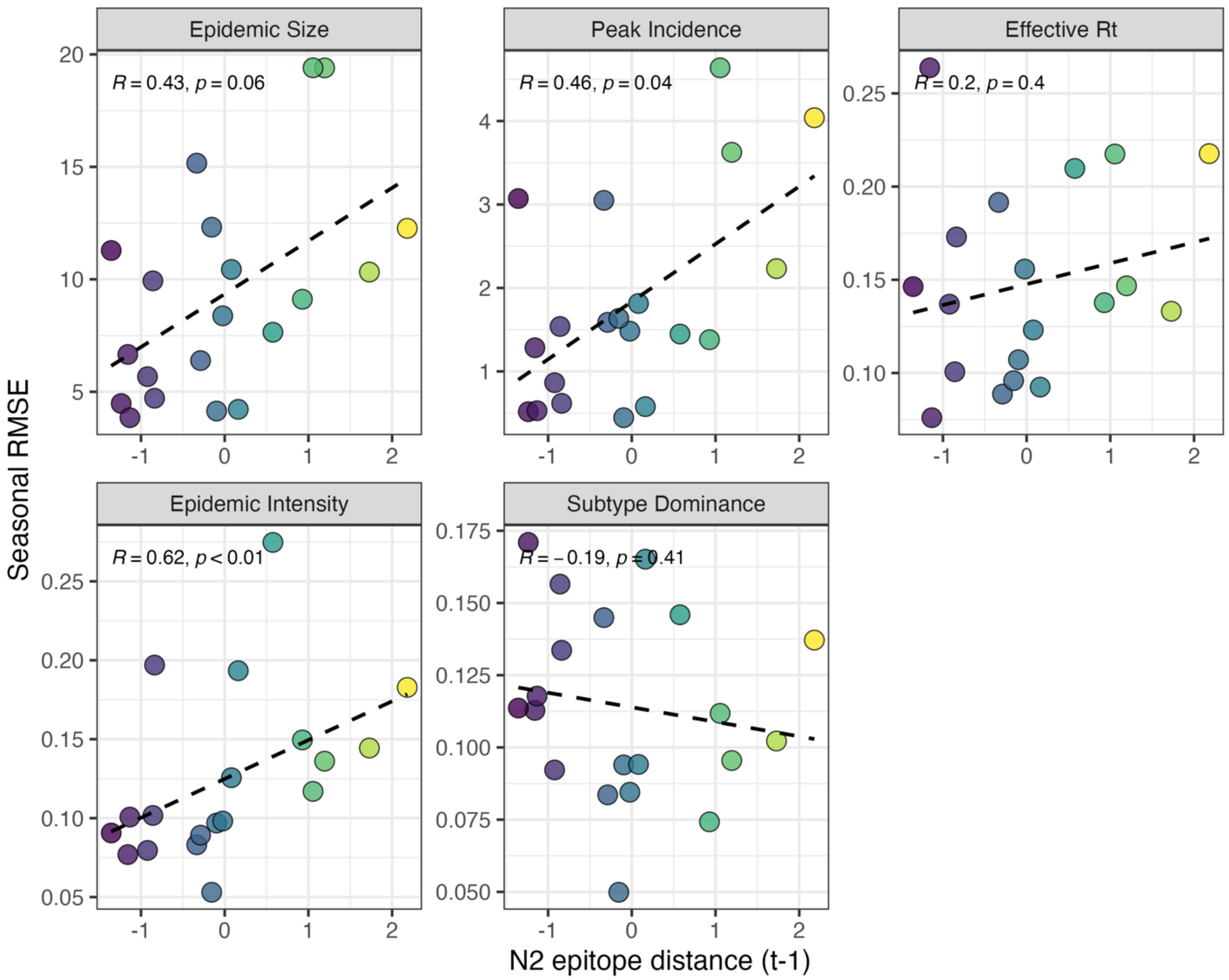

Figures S22. Relationships between the predictive accuracy of random forest models and N2 epitope distance Root mean squared errors between observed and model-predicted values were averaged across regions for each season, and results are facetted according to epidemic metric. Point color corresponds to the degree of N2 epitope distance in viruses circulating in season t relative to those circulating in the prior season ($t-1$), with bright yellow points indicating seasons with greater antigenic novelty. Spearman correlation coefficients and associated P-values are provided in the top left section of each facet.
