# Supplementary material for: Antigenic drift and subtype interference shape A(H3N2) epidemic dynamics in the United States
Source: medRxiv. 2023 Oct 3:2023.10.02.23296453. Preprint. [Version 1] doi: 10.1101/2023.10.02.23296453 (PMC10593063; doi:10.1101/2023.10.02.23296453)
Supplement: Supplementary file 2 [file media-2.pdf]

Appendix 1

Perofsky et al. "Antigenic drift and subtype interference shape A(H3N2) epidemic dynamics in the United States"

GISAID Acknowledgements

WHO Collaborating Centre for Reference and Research on Influenza, Victorian Infectious Diseases Reference Laboratory, Australia; WHO Collaborating Centre for Reference and Research on Influenza, Chinese National Influenza Center, China; WHO Collaborating Centre for Reference and Research on Influenza, National Institute of Infectious Diseases, Japan; The Crick Worldwide Influenza Centre, The Francis Crick Institute, United Kingdom; WHO Collaborating Centre for the Surveillance, Epidemiology and Control of Influenza, Centers for Disease Control and Prevention, United States; Aalesund Sjukehus, Norway; ADImmune Corporation, Taiwan; ADPH Bureau of Clinical Laboratories, United States; Aichi Prefectural Institute of Public Health, Japan; Akershus University Hospital, Norway; Akita Research Center for Public Health and Environment, Japan; Alabama State Laboratory, United States; Alaska State Public Health Laboratory, United States; Alaska State Virology Lab, United States; Alfred Hospital, Australia; Aomori Prefectural Institute of Public Health and Environment, Japan; Aristotelian University of Thessaloniki, Greece; Arizona Department of Health Services, United States; Arkansas Department of Health, United States; Atlanta VA Medical Center, United States; Auckland Healthcare, New Zealand; Auckland Hospital, New Zealand; Austin Health, Australia; Baylor College of Medicine, United States; Baylor Scott and White Health, United States; California Department of Health Services, United States; Canberra Hospital, Australia; Cantacuzino Institute, Romania; Canterbury Health Services, New Zealand; Caribbean Epidemiology Center, Trinidad and Tobago; CDC Central Asia Office; CDC GAP Nigeria, Nigeria; CDC Kenya, Kenya; CEMIC University Hospital, Argentina; CENETROP, Bolivia; Center For Medical Microbiology, College of Public Health, University of the Philippines, Philippines; Center for Public Health and Environment, Hiroshima Prefectural Technology Research Institute, Japan; Center of Hygiene And Epidemiology, Kirov Oblast, Russian Federation; Center of Hygiene And Epidemiology, Yamalo-Nenets Autonomous Okrug, Russian Federation; Center of Hygiene And Epidemiology, The Republic Of Dagestan, Russian Federation; Central Health Laboratory, Mauritius; Central Laboratory of Public Health, Paraguay; Central Public Health Laboratory, Ministry of Health, Oman; Central Public Health Laboratory, Palestinian Territory; Central Public Health Laboratory, Papua New Guinea; Central Public Health Reference Laboratory, Sierra Leone; Central Research Institute for Epidemiology, Russian Federation; Central Virology Laboratory, Israel; Centre de Recherche Médicale et Sanitaire, Niger; Centre for Diseases Control and Prevention, Armenia; Centre for Infections, Health Protection Agency, United Kingdom; Centre National de Référence des Virus des Infections Respiratoires, France; Centre National de Référence du Virus Influenza Région Sud, France; Centre Pasteur du Cameroun, Cameroon; Centro de Investigación Regional Dr. Hideyo Noguchi, Mexico; Chiba City Institute of Health and Environment, Japan; Chiba Prefectural Institute of Public Health, Japan; Children's Mercy Hospital, United States; Children's Hospital Westmead, Australia; Chuuk State Hospital, Micronesia, Federated States of; City of El Paso Dept of Public Health, United States; City of Milwaukee Health Department, United States; Clinical Virology Unit, CDIM, Australia; Colorado Department of Health Lab, United States; Connecticut Department of Public Health, United States; Contiguo a Hospital Rosales, El Salvador; CSL Ltd, United States; Dallas County Health and Human Services, United States; DC Public Health Lab, United States; Delaware Public Health Lab, United States; Departamento de Laboratorio de Salud Publica, Uruguay; Department of Clinical Virology, University College London Hospitals NHS Foundation Trust, United Kingdom; Department of Health, Hong Kong; Department of Public Health, Niigata City, Japan; Department of Virology, Medical University Vienna, Austria; Disease Investigation Centre Wates (BBVW), Australia; Dorevitch Pathology, Australia; Drammen Hospital/Vestreviken HF, Norway; Ehime Prefecture Institute of Public Health and Environmental Science, Japan; Erasmus Medical Center, Netherlands; Erasmus University of Rotterdam, Netherlands; Ethiopian Health and Nutrition Research Institute (EHNRI), Ethiopia; Ethiopian Public Health Institute, Ethiopia; Evandro Chagas Institute, Brazil; Facultad de Medicina, Spain; FBUZ Center for Hygiene and Epidemiology, Russian Federation; Florida Department of Health, United States; Fred Hutchinson Cancer Research Center, United States; Fukui Prefectural Institute of Public Health, Japan; Fukuoka City Institute for Hygiene and the Environment,

Japan; Fukuoka Institute of Public Health and Environmental Sciences, Japan; Fukushima Prefectural Institute of Public Health, Japan; Gart Naval General Hospital, United Kingdom; Georgia Public Health Laboratory, United States; Gifu Municipal Institute of Public Health, Japan; Gifu Prefectural Institute of Health and Environmental Sciences, Japan; Government Virus Unit, Hong Kong; Gunma Prefectural Institute of Public Health and Environmental Sciences, Japan; Hackensack University Medical Center, United States; Hamamatsu City Health Environment Research Center, Japan; Haukeland University Hospital, Dept. of Microbiology, Norway; Health Forde, Department of Microbiology, Norway; Health Protection Agency, United Kingdom; Health Protection Inspectorate, Estonia; Hellenic Pasteur Institute, Greece; Helsinki University Central Hospital, Finland; Hiroshima City Institute of Public Health, Japan; Hobart Pathology, Australia; Hokkaido Institute of Public Health, Japan; Hôpital Cantonal Universitaire de Geneves, Switzerland; Hôpital Charles Nicolle, Tunisia; Hôpital Georges L. Dumont, Canada; Hospital Clinic de Barcelona, Spain; Houston Department of Health and Human Services, United States; Hyogo Prefectural Institute of Public Health and Consumer Sciences, Japan; Ibaraki Prefectural Institute of Public Health, Japan; International Centre For Diarrhoeal Disease Research, Bangladesh; Illinois Department of Public Health, United States; Indiana State Department of Health Laboratories, United States; Infectology Center of Latvia, Latvia; Innlandet Hospital Trust, Division Lillehammer, Department for Microbiology, Norway; INRB Service De Virologie, Democratic Republic of the Congo; Institut Fédératif de Recherche Lyon, France; Institut Louis Malardé Clinical Laboratory, French Polynesia; Institut National d'Hygiène, Morocco; Instituto Nacional de Investigación en Salud Pública, Ecuador; Institut National de Recherches en Sante Publique, Mauritania;; Institut de Recherche en Sciences de la Santé, Burkina Faso; Institut Pasteur d'Algerie, Algeria; Institut Pasteur de Bangui, Central African Republic; Institut Pasteur de Dakar, Senegal; Institut Pasteur de Madagascar, Madagascar; Institut Pasteur in Cambodia, Cambodia; Institut Pasteur New Caledonia, New Caledonia; Institut Pasteur, France; Institut Penyelidikan Perubatan, Malaysia; Institute National D'Hygiene, Togo; Institute of Environmental Science and Research, New Zealand; Institute of Environmental Science and Research, Tonga; Institute For Biomedical Sciences, Suriname; Institute of Environmental Science & Research, New Zealand; Institute of Epidemiology and Infectious Diseases, Ukraine; Institute of Epidemiology Disease Control and Research, Bangladesh; Institute of Immunology and Virology Torlak, Serbia; Institute of Medical and Veterinary Science (IMVS), Australia; Institute of Public Health, Serbia; Institute of Public Health, Albania; Institute of Public Health, Montenegro; Institute Pasteur du Cambodia, Cambodia; Instituto Adolfo Lutz, Brazil; Instituto Conmemorativo Gorgas de Estudios de la Salud, Panama; Instituto De Diagnostico Y Referencia Epidemiologicos, Mexico;Instituto de Salud Carlos III, Spain; Instituto de Salud Publica de Chile, Chile; Instituto Nacional de Enfermedades Infecciosas, Argentina; Instituto Nacional de Higiene Rafael Rangel, Venezuela, Bolivia; Instituto Nacional de Laboratoriosde Salud (INLASA), Bolivia; Instituto Nacional de Salud de Columbia, Colombia; Instituto Nacional de Saude, Portugal; Iowa State Hygienic Laboratory, United States; IRSS, Burkina Faso; Ishikawa Prefectural Institute of Public Health and Environmental Science, Japan; ISS, Italy; Istanbul University, Turkey; Istituto Di Igiene, Italy; Istituto Superiore di Sanità, Italy; Ivanovsky Research Institute of Virology RAMS, Russian Federation; Jiangsu Provincial Center for Disease Control and Prevention, China; John Hunter Hospital, Australia; Kagawa Prefectural Research Institute for Environmental Sciences and Public Health, Japan; Kagoshima Prefectural Institute for Environmental Research and Public Health, Japan; Kanagawa Prefectural Institute of Public Health, Japan; Kansas Department of Health and Environment, United States; Kawasaki City Institute of Public Health, Japan; KEMRI Wellcome Trust Research Programme, Kenya; Kentucky Division of Laboratory Services, United States; Kitakyusyu City Institute of Environmental Sciences, Japan; Klinisk Mikrobiologi, Hallands Sjukhus Halmstad, Sweden; Klinisk Mikrobiologi, Karolinska Universitetslaboratoriet, Karolinska Universitetssjukhuset Solna, Sweden; Klinisk Mikrobiologi, Laboratoriemedicin, Norrlands Universitetssjukhus Umea, Sweden; Klinisk Mikrobiologi, Sahlgrenska Universitetssjukhuset Goteborg, Sweden; Kobe Institute of Health, Japan; Kochi Public Health and Sanitation Institute, Japan; Kumamoto City Environmental Research Center, Japan; Kumamoto Prefectural Institute of Public Health and Environmental Science, Japan; Kyoto City Institute of Health and Environmental Sciences, Japan; Kyoto Prefectural Institute of Public Health and Environment, Japan; Laboratoire De Santé Publique Du Québec, Canada; Laboratoire National de Sante Publique, Haiti; Laboratoire National de Sante, Luxembourg; Laboratório Central do Estado do Paraná, Brazil;

Laboratorio Central do Estado do Rio de Janeiro, Brazil; Laboratorio de Investigacion/Centro de Educacion Medica y Amistad Dominico Japones (CEMADOJA), Dominican Republic; Laboratorio De Isolamento Viral, Mozambique; Laboratorio De Referencia Nacional Virus Respiratorios, Instituto Nacional De Salud, Peru; Laboratorio De Saude Publico, Macao; Laboratorio de Virologia, Direccion de Microbiologia, Nicaragua; Laboratorio de Virus Respiratorio, Mexico; Laboratorio Di Virologia, Azienda Ospedaliero Universitaria Ospedali Riuniti Ancona, Italy; Laboratorio Nacional de Influenza, Costa Rica; Laboratorio Nacional De Salud Guatemala, Guatemala; Laboratorio Nacional de Virologia, Honduras; Laboratory Directorate, Jordan; Laboratory for Virology, National Institute of Public Health, Slovenia; Laboratory of Influenza and ILI, Belarus; LACEN/ES Laboratório Central de Saúde Pública do Estado do Espirito Santo, Brazil; LACEN/RS - Laboratório Central de Saúde Pública do Rio Grande do Sul, Brazil; LACEN-SC - Laboratório Central de Saúde Pública do Estado de Santa Catarina; Landspitali - University Hospital, Iceland; Lismore Base Hospital, Australia; Lithuanian AIDS Center Laboratory, Lithuania; Los Angeles Quarantine Station, CDC Quarantine Epidemiology and Surveillance Team, United States; Louisiana Department of Health and Hospitals, United States; Maine Health and Environmental Testing Laboratory, United States; Marshfield Clinic Research Foundation, United States; Maryland Department of Health and Mental Hygiene, United States; Massachusetts Department of Public Health, United States; Mater Dei Hospital, Malta; Medical Research Institute, Sri Lanka; Medical University Vienna, Austria; Melbourne Pathology, Australia; Michigan Department of Community Health, United States; Microbiology Services Colindale, Public Health England, United Kingdom; Mie Prefecture Health and Environment Research Institute, Japan; Mikrobiologisk laboratorium, Sykehuset i Vestfold, Norway; Ministry of Health and Population, Egypt; Ministry of Health of Ukraine, Ukraine; Ministry of Health, Bahrain; Ministry of Health, Kiribati; Ministry of Health, Lao, People's Democratic Republic; Ministry of Health, NIHRD, Indonesia; Ministry of Health, Maldives; Ministry of Health, Oman; Ministry of Health Riyadh, Saudi Arabia; Ministry of Health, Singapore; Ministry of Health, Thailand; Minnesota Department of Health, United States; Mississippi Public Health Laboratory, United States; Missouri Department of Health and Senior Services, United States; Miyagi Prefectural Institute of Public Health and Environment, Japan; Miyazaki Prefectural Institute for Public Health and Environment, Japan; Molde Hospital, Laboratory for Medical Microbiology, Norway; Monash Medical Centre, Australia; Montana Laboratory Services Bureau, United States; Montana Public Health Laboratory, United States; Nagano City Health Center, Japan; Nagano Environmental Conservation Research Institute, Japan; Nagasaki Prefectural Institute For Environment Research and Public Health, Japan; Nagoya City Public Health Research Institute, Japan; NAMRU-2 U.S. Naval Medical Research Unit-2, Cambodia; NAMRU-2 U.S. Naval Medical Research Unit-2, Indonesia; NAMRU-6 U.S. Naval Medical Research Unit-6, Peru; Nara Prefectural Institute for Hygiene and Environment, Japan; National Center for Communicable Diseases, Mongolia; National Center For Epidemiology, National Influenza Center, Hungary; National Center for Laboratory and Epidemiology, Laos; National Centre for Disease Control and Public Health, Georgia; National Centre for Preventive Medicine, Moldova, Republic of; National Centre for Scientific Services for Virology and Vector Borne Diseases, Fiji; National Health Laboratory, Japan; National Health Laboratory, Myanmar; National Influenza Center CVD-Mali, Mali; National Influenza Center French Guiana and French Indies, French Guiana; National Influenza Center, Brazil; National Influenza Center, Mongolia; National Influenza Centre for Northern Greece, Greece; National Influenza Centre of Iraq, Iraq; National Influenza Lab, Tanzania, United Republic of; National Influenza Reference Laboratory, Nigeria; National Institute for Communicable Disease, South Africa; National Institute for Health and Welfare, Finland; National Institute For Medical Research, United Kingdom; National Institute For Public Health and The Environment (RIVM), Netherlands; National Institute of Health, Korea, Republic of; National Institute of Health, Pakistan; National Institute of Hygiene and Epidemiology, Vietnam; National Institute of Infectious Diseases (NIID), Japan; National Institute of Public Health of Kosova, Kosovo; National Institute of Public Health - National Institute of Hygiene, Poland; National Institute of Public Health, Czech Republic; National Institute of Virology, India; National Microbiology Laboratory, Health Canada, Canada; National Public Health Institute of Slovakia, Slovakia; National Public Health Laboratory, Cambodia; National Public Health Laboratory, Ministry of Health, Singapore, Singapore; National Public Health Laboratory, Nepal; National Public Health Laboratory, Singapore; National Reference Laboratory, Kazakhstan; National Referral Hospital, Solomon Islands; National University Hospital, Singapore; National Virology

Laboratory, Center Microbiological Investigations, Kyrgyzstan; National Veterinary Institute, Sri Lanka; National Virus Reference Laboratory, Ireland; Naval Health Research Center, United States; NCDC Public Health Reference Laboratory, Nigeria; Nebraska Public Health Lab, United States; Nevada State Health Laboratory, United States; New Hampshire Public Health Laboratories, United States; New Jersey Department of Health and Senior Services, United States; New Mexico Department of Health, United States; New York City Department of Health, United States; New York Presbyterian Hospital Columbia University Medical Center, Microbiology Department, United States; New York State Department of Health, United States; Nicosia General Hospital, Cyprus; Niigata City Institute of Public Health and Environment, Japan; Niigata Prefectural Institute of Public Health and Environmental Sciences, Japan; Niigata University, Japan; N ingbo International Travel Healthcare Center, China; North Carolina State Laboratory of Public Health, United States; North Dakota Department of Health, United States; Norwegian Institute of Public Health, Norway; Ohio Department of Health Laboratories, United States; Oita Prefectural Institute of Health and Environment, Japan; Okayama Prefectural Institute for Environmental Science and Public Health, Japan; Okinawa Prefectural Institute of Health and Environment, Japan; Oklahoma State Department of Health, United States; Ontario Agency for Health Protection and Promotion (OAHPP), Canada; Oregon Public Health Laboratory, United States; Osaka City Institute of Public Health and Environmental Sciences, Japan; Osaka Prefectural Institute of Public Health, Japan; Oslo University Hospital, Ulleval Hospital, Dept. of Microbiology, Norway; Ostfold Hospital - Fredrikstad, Dept. of Microbiology, Norway; Oswaldo Cruz Institute - FIOCRUZ - Laboratory of Respiratory Viruses and Measles (LVRS), Brazil; Papua New Guinea Institute of Medical Research, Papua New Guinea; Pasteur Institut of Côte D'ivoire, Côte D'ivoire; Pasteur Institute of Ho Chi Minh City, Vietnam; Pasteur Institute, Influenza Laboratory, Vietnam; Pathwest QE II Medical Centre, Australia; Pennsylvania Department of Health, United States; Prince of Wales Hospital, Australia; Princess Margaret Hospital for Children, Australia; Provincial Laboratory For Public Health For Northern Alberta, Canada; Provincial Laboratory for Public Health, Alberta Health Services, Canada; Provincial Laboratory of Public Health For Southern Alberta, Canada; Public Health Agency of Sweden, Sweden; Public Health Laboratory Services Branch, Centre for Health Protection, Hong Kong; Public Health Laboratory, Barbados; Public Health Laboratory, Virology Unit, Kuwait; Public Health Ontario, Canada; Public Health Wales Microbiology, United Kingdom; Puerto Rico Department of Health, Puerto Rico; Queensland Health Forensic and Scientific Services, Australia; Queensland Health Scientific Services, Australia; Rafic Hariri University Hospital, Lebanon; Refik Saydam National Public Health Agency, Turkey; Regent Seven Seas Cruises, United States; Republic Institute For Health Protection, Macedonia; Republic of Nauru Hospital, Nauru; Republican Anti Plague Station, Azerbaijan, Republic of; Research Institute for Environmental Sciences and Public Health of Iwate Prefecture, Japan; Research Institute of Health Sciences (IRSS), Burkina Faso; Research Institute of Tropical Medicine, Philippines; Rhode Island Department of Health, United States; Robert-Koch-Institute, Germany; Roy Romanow Provincial Laboratory, Canada; Royal Centre For Disease Control, Bhutan; Royal Children's Hospital, Australia; Royal Darwin Hospital, Australia; Royal Hobart Hospital, Australia; Royal Melbourne Hospital, Australia; Russian Academy of Medical Sciences, Russian Federation; Rwanda Biomedical Center, National Reference Laboratory, Rwanda; Saga Prefectural Institute of Public Health and Pharmaceutical Research, Japan; Sagamihara City Laboratory of Public Health, Japan; Saitama City Institute of Health Science and Research, Japan; Saitama Institute of Public Health, Japan; Saitama Medical University, Japan; Sakai City Institute of Public Health, Japan; San Antonio Metropolitan Health, United States; Sandringham, National Institute for Communicable Disease, South Africa; Sapporo City Institute of Public Health, Japan; Sciensano, Scientific Institute of Public Health, Belgium; Scientific Institute of Public Health, Belgium; Seattle and King County Public Health Lab, United States; Sendai City Institute of Public Health, Japan; Servicio de Microbiología Complejo Hospitalario de Navarra, Spain; Servicio de Microbiología Hospital Donostia, Spain; Servicio de Microbiología Hospital Meixoeiro, Spain; Servicio de Microbiología Hospital Miguel Servet, Spain; Servicio de Microbiología Hospital Ramón y Cajal, Spain; Servicio de Microbiología Hospital San Pedro de Alcántara, Spain; Servicio de Microbiología Hospital Universitario de Gran Canaria Doctor Negrín, Spain; Servicio de Microbiología Hospital Universitario Son Espases, Spain; Servicio de Microbiología Hospital Virgen de las Nieves, Spain; Servicio de Virosis Respiratorias INEI-ANLIS Carlos G. Malbran, Argentina; Seychelles Public Health Laboratory, Seychelles; Sheikh Khalifa Medical City, United Arab Emirates;

Shanghai International Travel Healthcare Center, China; Shiga Prefectural Institute of Public Health, Japan; Shimane Prefectural Institute of Public Health and Environmental Science, Japan; Shizuoka City Institute of Environmental Sciences and Public Health , Japan; Shizuoka Institute of Environment and Hygiene, Japan; Singapore General Hospital, Singapore; Sorlandet Sykehus HF, Dept. of Medical Microbiology, Norway; South Carolina Department of Health, United States; South Dakota Public Health Lab, United States; Southern Nevada Public Health Lab, United States; Spokane Regional Health District, United States; St. Jude's Children's Research Hospital, United States; St. Olavs Hospital HF, Dept. of Medical Microbiology, Norway; State Agency, Infectology Center of Latvia, Latvia; State of Hawaii Department of Health, United States; State of Idaho Bureau of Laboratories, United States; State Research Center of Virology and Biotechnology Vector, Russian Federation; Statens Serum Institute, Denmark; Stavanger Universitetssykehus, Avd. for Medisinsk Mikrobiologi, Norway; Supreme Health Council, Qatar; Swedish Institute for Infectious Disease Control, Sweden; Taiwan CDC, Taiwan; Tan Tock Seng Hospital, Singapore; Tarrant County Public Health, Texas, United States; Tehran University of Medical Sciences, Iran; Tennessee Department of Health Laboratory-Nashville, United States; Texas Children's Hospital, United States; Texas Department of State Health Services, United States; Texas Department of State Health Services, South Texas Laboratory, United States; Thai National Influenza Center, Thailand; Thailand MOPH-U.S. CDC Collaboration (IEIP), Thailand; The Nebraska Medical Center, United States; The NIAID Influenza Genome Sequencing Consortium, United States; Thüringer Landesamt für Verbraucherschutz, Germany; Tochigi Prefectural Institute of Public Health and Environmental Science, Japan; Tokushima Prefectural Centre for Public Health and Environmental Sciences, Japan; Tokyo Metropolitan Institute of Public Health, Japan; Tottori Prefectural Institute of Public Health and Environmental Science, Japan; Toyama Institute of Health, Japan; U.S. Air Force School of Aerospace Medicine, United States; U.S. AMC AFRIMS Department of Virology, Thailand; U.S. Army Medical Research Unit, Kenya (USAMRU-K), Geis Human Influenza Program, Kenya; Uganda Virus Research Institute (UVRI), National Influenza Center, Uganda; Unilabs Laboratoriemedicin Stockholm Solna, Sweden; Unilabs Laboratoriemedicin Vastra Gotaland, Skaraborgs Sjukhus Skovde, Sweden; Universidad de Valladolid, Spain; Universitetssykehuset Nord-Norge HF, Norway; University Malaya, Malaysia; University of Genoa, Italy; University of Ghana, Ghana; University of Michigan SPH EPID, United States; University of The West Indies, Jamaica; University of Vienna, Austria; University of Virginia, Medical Labs/Microbiology, United States; University Teaching Hospital, Zambia; Uoc Policlinico Di Bari Dimo, Italy; UPMC-CLB Dept of Microbiology, United States; Utah Department of Health, United States; Utah Public Health Laboratory, United States; Utsunomiya City Institute of Public Health and Environment Science, Japan; VACSERA, Egypt; Vanderbilt University Medical Center, United States; Vefa Center, Tajikistan; Vermont Department of Health Laboratory, United States; Victorian Infectious Diseases Reference Laboratory, Australia; Virginia Division of Consolidated Laboratories, United States; Virus Research Center, Sendai Medical Center, Japan; Wakayama City Institute of Public Health, Japan; Wakayama Prefectural Research Center of Environment and Public Health, Japan; Walter Reed Army Institute of Research, United States; Washington State Public Health Laboratory, United States; West Virginia Office of Laboratory Services, United States; Westchester County Department of Laboratories and Research, United States; Westmead Hospital, Australia; National Influenza Centre Russian Federation, Russian Federation; WHO National Influenza Centre, National Institute of Medical Research (NIMR), Thailand; WHO National Influenza Centre, Norway; Wisconsin State Laboratory of Hygiene, United States; Wyoming Public Health Laboratory, United States; Yamagata Prefectural Institute of Public Health, Japan; Yamaguchi Prefectural Institute of Public Health and Environment, Japan; Yamanashi Institute for Public Health, Japan; Yap State Hospital, Micronesia; Yokohama City Institute of Health, Japan; Yokosuka Institute of Public Health, Japan
